# The Bug‐Network (BugNet): A Global Experimental Network Testing the Effects of Invertebrate Herbivores and Fungal Pathogens on Plant Communities and Ecosystem Function in Open Ecosystems

**DOI:** 10.1002/ece3.72111

**Published:** 2025-10-09

**Authors:** Anne Kempel, George C. Adamidis, José D. Anadón, Joe Atkinson, Harald Auge, Dimitrios Avtzis, Benedicte Bachelot, Maral Bashirzadeh, Julien L. Bota, Aimee Classen, Ioannis Constantinou, Mick Crawley, Tonia de Bellis, Petr Dostal, Anne Ebeling, Nico Eisenhauer, David J. Eldridge, Gustavo Encina, Catalina Estrada, Susan Everingham, Nicolas Fanin, Yanhao Feng, Mario Gaspar, Leana Gooriah, Pamela Graff, Elizabeth Gusmán Montalván, Pamela Gusmán Montalván, Tamara R. Hartke, Linjia Huang, Malte Jochum, Karin Kaljund, Ilias Karmiris, Kadri Koorem, Lotte Korell, Anna‐Liisa Laine, Gaëtane le Provost, Jean‐Philippe Lessard, Mu Liu, Xiang Liu, Yanjie Liu, Juan Llancabure, Sidonie Loïez, Alejandro Loydi, Hugo Marrero, Shelby Gockel, Adrián Montoya, Zuzana Münzbergová, Yujie Niu, David Ott, Mariano Oyarzabal, Maria Panitsa, Effimia Papatheodorou, Frida I. Piper, Kersti Püssa, Karin Rand, Hugo Saiz, Nathan J. Sanders, Martin Schädler, Christoph Scherber, Marina Semchenko, Siim‐Kaarel Sepp, Manzoor Ahmad Shah, Ishrat Shaheen, Claudia Stein, Jana Stewart, Zhuangsheng Tang, Georg Tschan, Saskya van Nouhuys, Martijn L. Vandegehuchte, Millie Vernon, Sonali V. R., Jianyong Wang, Yao Xiao, Fotios Xystrakis, Jie Yang, Siwei Yang, Konstantina Zografou, Eric Allan

**Affiliations:** ^1^ WSL Institute for Snow and Avalanche Research SLF Davos Switzerland; ^2^ Climate Change, Extremes and Natural Hazards in Alpine Regions Research Centre CERC Davos Switzerland; ^3^ Department of Biology University of Patras Rio Greece; ^4^ Pyrenean Institute of Ecology (IPE‐CSIC) Spanish National Research Council Zaragoza Spain; ^5^ School of Biological Sciences, Faculty of Science, Engineering and Technology University of Adelaide Adelaide South Australia Australia; ^6^ Helmholtz Centre for Environmental Research – UFZ Halle Germany; ^7^ German Centre for Integrative Biodiversity Research (iDiv) Halle‐Jena‐Leipzig Leipzig Germany; ^8^ Forest Research Institute – Hellenic Agricultural Organization Demeter Thessaloniki Greece; ^9^ Department of Biology Oklahoma State University Stillwater Oklahoma USA; ^10^ Department of Biology, Faculty of Science University of Mazandaran Babolsar Iran; ^11^ The University of Michigan Biological Station, University of Michigan Pellston Michigan USA; ^12^ Department of Ecology and Evolutionary Biology University of Michigan Ann Arbor Michigan USA; ^13^ Institute of Biology Leipzig University Leipzig Germany; ^14^ Department of Life Sciences Imperial College London Ascot UK; ^15^ Biology Department Dawson College Montreal Quebec Canada; ^16^ Department of Biology Concordia University Montreal Quebec Canada; ^17^ Institute of Botany of the Czech Academy of Sciences Pruhonice Czech Republic; ^18^ Institute of Biodiversity, Ecology and Evolution, Population Ecology University Jena Jena Germany; ^19^ Centre for Ecosystem Science, School of Biological, Earth and Environmental Sciences University of New South Wales Sydney New South Wales Australia; ^20^ Instituto de Ciencias Biológicas, Universidad de Talca Talca Chile; ^21^ Institute of Plant Sciences and Oeschger Centre for Climate Change Research University of Bern Bern Switzerland; ^22^ Hawkesbury Institute for the Environment Western Sydney University Penrith New South Wales Australia; ^23^ INRAE, Bordeaux Sciences Agro ISPA Villenave d'Ornon France; ^24^ State Key Laboratory of Herbage Improvement and Grassland Agro‐Ecosystems, College of Pastoral Agriculture Science and Technology Lanzhou University Lanzhou China; ^25^ INRAE, Bordeaux Science Agro, ISVV SAVE Villenave d'Ornon France; ^26^ CONICET and Agencia de Extensión Rural Coronel Suárez EEA Cesáreo Naredo, INTA Coronel Suárez Provincia de Buenos Aires Argentina; ^27^ Laboratorio de Ecología Tropical y Servicios Ecosistémicos—EcoSs Lab, Departamento de Ciencias Biológicas y Agropecuarias Universidad Técnica Particular de Loj Loja Ecuador; ^28^ Alumni Biología‐Universidad Técnica Particular de Loja, San Cayetano Loja Ecuador; ^29^ Leibniz‐Institute for the Analysis of Biodiversity Change Bonn Germany; ^30^ Department of Global Change Ecology, Biocenter University of Würzburg Würzburg Germany; ^31^ Department of Botany, Institute of Ecology and Earth Sciences University of Tartu Tartu Estonia; ^32^ Research Centre for Ecological Change, Organismal and Evolutionary Biology Research Programme, Faculty of Environmental and Biological Sciences University of Helsinki Helsinki Finland; ^33^ State Key Laboratory of Herbage Improvement and Grassland Agro‐Ecosystems, College of Ecology Lanzhou University Lanzhou China; ^34^ State Key Laboratory of Black Soils Conservation and Utilization, Northeast Institute of Geography and Agroecology, Chinese Academy of Sciences Changchun China; ^35^ Instituto de Ecología y Biodiversidad (IEB) Ñuñoa, Santiago Chile; ^36^ Norwegian University of Science and Technology Trondheim Norway; ^37^ Centro de Recursos Naturales Renovables de la Zona Semiárida (CERZOS, CONICET/UNS) Bahía Blanca Argentina; ^38^ Departamento de Biología, Bioquímica y Farmacia UNS Bahía Blanca Argentina; ^39^ Department of Biosystems & Agricultural Engineering Oklahoma State University Stillwater Oklahoma USA; ^40^ Department of Botany, Faculty of Science Charles University Prague Czech Republic; ^41^ Key Laboratory of Grassland Ecosystem of the Ministry of Education, College of Grassland Science Gansu Agricultural University Lanzhou Gansu China; ^42^ Disturbance Ecology and Vegetation Dynamics, Bayreuth Center of Ecology and Environmental Research (BayCEER) University of Bayreuth Bayreuth Germany; ^43^ Departamento de Agronomía Universidad Nacional del Sur Bahía Blanca Provincia de Buenos Aires Argentina; ^44^ Department of Ecology, School of Biology, Faculty of Sciences Aristotle University of Thessaloniki Thessaloniki Greece; ^45^ Instituto de Ecología y Biodiversidad (IEB) Santiago Chile; ^46^ Departamento de Ciencias Agrarias y Medio Natural, Escuela Politécnica Superior, Instituto Universitario de Investigación en Ciencias Ambientales de Aragón (IUCA) Universidad de Zaragoza Huesca Spain; ^47^ Netherlands Institute of Ecology (NIOO‐KNAW) Wageningen the Netherlands; ^48^ Department of Botany University of Kashmir Jammu & Kashmir India; ^49^ Department of Biology & Environmental Sciences Auburn University at Montgomery Montgomery Alabama USA; ^50^ Indian Institute of Science Bengaluru India; ^51^ School of Biosciences Cardiff University Cardiff UK; ^52^ Institute of Grassland Science, Key Laboratory of Vegetation Ecology of the Ministry of Education, Jilin Songnen Grassland Ecosystem National Observation and Research Station Northeast Normal University Changchun China; ^53^ Engineering Technology Research Center of Qinghai‐Tibet Plateau, Alpine Grassland Ecology Restoration, Seda Grassland Ecology Sichuan Field Scientific Observation and Research Station Sichuan Academy of Grassland Science Chengdu China; ^54^ Department of Biological Applications and Technology University of Ioannina Ioannina Greece; ^55^ Centre for Development and Environment University of Bern Bern Switzerland

**Keywords:** exclusion experiment, fungal pathogens, fungicide, globally coordinated experimental network, insect herbivores, insecticide, maintenance of biodiversity, molluscicide, mollusks

## Abstract

Plants are consumed by a variety of organisms, including herbivores and pathogens, which significantly impact plant biomass, diversity, community composition, and ecosystem functioning. While the impacts of vertebrate herbivores are well established, the effects of consumer groups such as insect herbivores, mollusks, and fungal pathogens on plant communities are less clear and remain understudied in many systems. Existing evidence of how they affect plant biomass, diversity, and community composition is mixed, and most studies have focused on individual consumer groups in isolation. However, different consumer groups interact with each other, directly or indirectly, in ways that alter their impacts on plants, and the consequences of these interactions for plant community structure and ecosystem function remain understudied. Further, consumer impacts vary across environmental gradients and likely depend on abiotic conditions such as climate, soil type, or elevation, and biotic conditions such as plant productivity, diversity, or community composition. Existing studies testing the impacts of invertebrate herbivores and fungal pathogens on plant communities differ substantially in methodology, making generalities across large scales difficult. This calls for experimental approaches that implement standardized protocols across many sites. Here, we introduce and report on the methodology of a novel global research network, The Bug‐Network (BugNet), that implements standardized consumer‐reduction experiments across 5 continents and 18 countries in diverse, herbaceous‐ or shrub‐dominated ecosystems to investigate: (1) the influence of fungal pathogens, insect herbivores, and mollusks on plant diversity and ecosystem functioning, (2) interactions among these consumer groups, and (3) the abiotic and biotic drivers of context‐dependent consumer impacts. BugNet aims to advance a predictive understanding of plant‐consumer interactions in order to test fundamental ecological hypotheses and improve predictions of global change impacts on biodiversity and ecosystem functioning.

## Graphical abstract

The Bug‐Network (BugNet) is a novel global collaborative research network that implements standardized consumer‐reduction experiments in herbaceous‐ or shrub‐dominated ecosystems to assess the impacts of invertebrate herbivores and fungal pathogens on plant communities and ecosystem functioning. By examining consumer effects across diverse ecosystems and environmental gradients, BugNet aims to uncover general patterns in plant–consumer interactions and improve predictions of global change impacts.

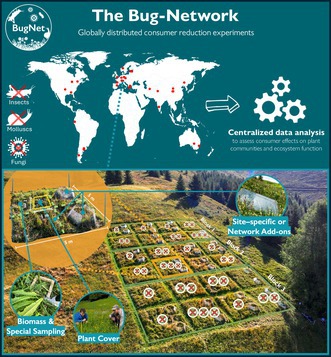

## 1 Introduction

Plants are the primary producers in terrestrial ecosystems, thus it is not surprising that they are fed on and are infested by a large range of consumers, such as herbivores and pathogens. Consumers can affect plant communities by reducing plant biomass and altering competitive interactions between plant species: promoting plant diversity when feeding on dominant species (Bever et al. 2015; Chesson 2000; Holt 1977; Terborgh 2015) and reducing it when targeting subordinate ones (Pacala and Crawley 1992; Peters and Shaw 1996). They may also shift plant community composition toward dominance by slower‐growing, better‐defended species if they prefer to consume fast‐growing, less‐defended plants (Coley et al. 1985). While it is widely appreciated that vertebrate herbivores can have strong impacts on primary production, plant species composition, plant diversity, and other ecosystem processes (e.g., Borer, Harpole, et al. 2014; Borer, Seabloom, et al. 2014; Duffy et al. 2003; Jia et al. 2018), the effects of other consumer groups, such as insect and mollusk herbivores and foliar pathogens, are less well understood (Borer et al. 2015). Of these, insects are probably best studied, but results from experiments excluding insects from plant communities in the field are less consistent than results from vertebrate exclusion experiments. Some experiments show insects strongly reduce plant biomass and alter plant community composition and diversity (Allan and Crawley 2011; Carson and Root 2000), while others show no effects or contradictory patterns (Coupe et al. 2009; Coupe and Cahill 2003). A meta‐analysis on insect suppression studies therefore found no overall effect on plant community characteristics (Jia et al. 2018). Studies reducing foliar pathogens and mollusks in plant communities are rarer, particularly in herbaceous and shrub‐dominated systems, although some have been conducted (e.g., Allan and Crawley 2011; Cappelli et al. 2020; Korell et al. 2016; Liu et al. 2022). In addition, only a few studies have directly compared the effects of different consumer groups in the same experiment (Allan and Crawley 2011; Stein et al. 2010). More general information on overall consumer impacts across sites is important to understand the mechanisms that generate and maintain plant diversity and ecosystem functioning. Such knowledge is also important to better understand global changes, including ongoing insect declines (Eisenhauer et al. 2023; van Klink et al. 2020), shifts in herbivore and pathogen dynamics under climate change (Anderson et al. 2004; Chaloner et al. 2021; Singh et al. 2023), and changing consumer effects on the global carbon budget (Couture et al. 2015; Silfver et al. 2020).

While understanding the effects of individual consumer groups is important, these effects may depend strongly on which other groups of consumers are abundant in the community. Different groups of plant consumers can interact in ways that amplify, dampen, or otherwise alter their impacts on plant communities, and these interactions can occur through a range of mechanisms. Consumers can directly affect each other, for example, through feeding on each other (Eberl et al. 2020; Tack and Dicke 2013). They can also indirectly affect each other by changing plant community composition, biomass, plant physiology (plant defense and nutritional quality) or microclimate (Borer et al. 2009; Li et al. 2024). These interactions can lead to dramatic changes in the impact of one consumer group depending on the presence of another (Allan and Crawley 2011; Duffy et al. 2003; van Ruijven et al. 2005) and could generate complex indirect effects on plant communities. Ignoring interactions might therefore substantially under‐ or overestimate consumer effects on plant communities. While the impact of such interactions has been studied at the level of individual plant performance (e.g., Hauser et al. 2013; Morris et al. 2007), their effects at the community level remain less well understood, particularly for interactions between invertebrate herbivores and pathogens. Factorial (crossed) exclusions of different consumer groups have been used to test for such interactions within individual sites; however, studies comparing the strength of interactions across sites have very rarely been undertaken (Agrawal and Maron 2022). This highlights the need for a standardized, coordinated approach to better understand whether combined consumer effects are additive, synergistic, or compensatory at the plant community level.

A large body of theoretical and empirical work suggests that plant‐consumer interactions vary substantially in space and time (Table 1). Hence, the impact of plant consumers on plant communities might differ depending on many abiotic and biotic factors (e.g., Dobzhansky 1950; Ford et al. 2014). Understanding how these impacts vary is a key current challenge and would provide essential information to address a range of problems, from improving global change forecasts to predicting the efficacy of weed biocontrol (HilleRisLambers et al. 2012; Louthan et al. 2015). However, there is currently no consensus on how factors like nutrient availability, climate, or plant diversity drive variation in impact (Jia et al. 2018; Kempel et al. 2023; Maron, Klironomos, et al. 2014). Thus, to develop an understanding of the importance of different plant consumers for the diversity of plant communities and ecosystem functioning, we require replicated exclusion experiments across large environmental gradients across the globe. While there is a precedent for replicated global studies in other research questions (Bebout and Fox 2024; Borer, Harpole, et al. 2014; Wall et al. 2008), this scale of experimental manipulation has never been attempted for invertebrate herbivores and fungal pathogens.

**TABLE 1:** Overview of the key hypotheses and predictions to be tested within the Bug‐Network.

Hypotheses	Predictions
Top‐down control	Plant consumers affect the diversity of plant communities by altering competitive interactions between plant species (Bever et al. 2015; Chesson 2000; Holt 1977; Terborgh 2015). Consumers promote diversity if they preferentially feed on the more abundant plant species within a community and reduce diversity if they consume subordinate species (Pacala and Crawley 1992; Peters and Shaw 1996). *In BugNet, we will assess plant consumer effects on biodiversity, and whether they typically attack dominant or subordinate species*.
Growth‐defense trade‐off	The growth‐defense trade‐off hypothesis predicts that fast growing plant species are less defended and hence more preferred by consumers, which equalizes fitness between plant species (Coley et al. 1985). *In BugNet we will assess whether changes in the plant community due to consumer removal follows patterns predicted by the growth‐defense trade‐off and whether this shift is stronger for certain consumer groups*.
Indirect consumer effects	Different consumers are likely to interact with each other to generate indirect effects on plant communities (Wootton 1994). Interactions can emerge from effects of one consumer group on another, for example, if one consumer group reduces or increases the performance of another group (Eberl et al. 2020; Hatcher 1995), or through different effects of the consumers on the plant community (Biere and Bennett 2013; Dobson and Crawley 1994). The combined effects of interacting consumers can be compensatory if they inhibit each other, or consume different species or functional groups; in this case, the loss of one consumer group can have strong effects on plant community composition whereas their combined effects balance each other out. Their effect can also be superadditive (synergistic) if consumers benefit each other or if they consume the same plant species (Ritchie and Olff 1999); in this case, multiple consumer groups may alter plant communities more strongly than a single consumer group. *In BugNet, we will assess whether and how different consumer groups interact, to affect plant community structure. We will also test for competitive or facilitative interaction between groups by testing whether reductions in one group alter the abundance or activity of another group*.
Biodiversity‐functioning theory	A more diverse consumer community would have stronger impacts on plant productivity due to complementary resource use (Deraison et al. 2015; Duffy et al. 2003). Alternatively, a more diverse consumer community might also decrease impact if consumers compete and suppress the emergence of virulent consumer strains or species (Al‐Naimi et al. 2005; Becker et al. 2012). *In BugNet, we will assess the relationship between consumer diversity (group diversity) and consumer impact on plant biomass production or other functions*.
Biotic interaction	Consumers have a greater impact on plant productivity and diversity, as well as a higher specialization, at low latitudes compared to high latitudes (Dobzhansky 1950; Schemske et al. 2009). The mechanisms driving this latitudinal gradient are likely to emerge over long time scales and involve increased coevolution and speciation in more predictable and benign climates. For insects, there are currently supporting and contradicting studies (e.g., Liu et al. 2024; Moles and Ollerton 2016; Schemske et al. 2009), for pathogens there is one contradicting study (Nguyen et al. 2016). *In BugNet, we will assess how consumer impact relates to latitude and climatic variables*.
Resource availability	Plants from resource‐poor (nutrient poor) habitats are better defended, thus infection and consumer impact should be higher at high soil fertility and for species with acquisitive growth strategies (Coley et al. 1985; Wardle et al. 1997). However, acquisitive species might also be more tolerant (Cronin et al. 2010). For insects, there are inconsistent results from studies regarding herbivore abundance and herbivore biomass, meta‐analyses reveal no relation of impact with soil fertility (Haddad et al. 2000; Jia et al. 2018; Kempel et al. 2023; Maron, Baer, and Angert 2014; Perner et al. 2005). For pathogens, there are supporting studies regarding infection, but impact on plant biomass has been rarely assessed (Seabloom et al. 2018; Veresoglou et al. 2013). *In BugNet, we will assess how consumer damage, but also consumer impact is influenced by soil fertility*.
Host concentration/host regulation	Consumer impact is larger at high host plant abundance, leading to negative density dependence, which stabilizes diversity. The host concentration and host regulation hypothesis therefore predicts that specialist consumer impact is higher at low plant diversity (Keesing et al. 2006; Root 1973). Extending to the community level, these hypotheses predicts that high plant diversity would reduce consumer impact and abundance. There are several supporting studies for pathogens (Mitchell et al. 2002; Rottstock et al. 2014), however, evidence for negative effects of plant diversity on insect herbivore abundance and impact is more mixed in grasslands (Hertzog et al. 2016; Seabloom et al. 2017), suggesting that insect communities may be dominated by generalists. In forest, negative effects of tree diversity on pest attack are consistent for specialists but effects are more mixed for generalists (Jactel and Brockerhoff 2007). *In BugNet, we will test whether consumer impact is higher at low plant diversity and whether the strength of host concentration effects differs between consumer groups*.

Here we describe the key questions, objectives, and methodology of a novel global research network, the Bug‐Network (BugNet). BugNet aims to set up identical invertebrate herbivore and fungal reduction experiments to quantify plant community and ecosystem responses to insects, mollusks, and fungal pathogens in a wide range of herbaceous or shrub‐dominated ecosystems, from desert grasslands to arctic tundra and from heathlands to Mediterranean shrublands (Figure S1). BugNet aims to answer the following overarching questions:

1. How do different plant consumer groups influence plant biomass production, the diversity and composition of plant communities, and ecosystem functioning? Do their effects on plant communities vary among consumer types?
2. How do different plant consumer groups interact? For example, does reducing one group change the abundance, activity, or impact of another? How do these interactions affect plant communities?
3. What abiotic and biotic factors drive context‐dependency in consumer effects and interactions? Does consumer impact vary geographically with latitude, and how are consumer impacts shaped by factors such as climate, soil fertility, or plant diversity?

Addressing these questions will enable us to (i) answer open questions about the impact of understudied aboveground consumer groups on plant communities and ecosystem functioning, (ii) identify interacting effects of multiple consumer groups, (iii) test long‐standing ecological hypotheses about the abiotic and biotic factors driving variation in antagonistic interactions, and (iv) develop a predictive understanding of why and how antagonistic interactions vary in space. These insights will advance our understanding and provide a rigorous test of several long‐standing ecological hypotheses (Table 1). This will offer critical knowledge to address various urgent issues, such as enhancing predictions of global environmental changes and improving the accuracy of future forecasts. BugNet therefore tackles questions of fundamental and applied importance about the processes shaping species interactions and increases our ability to mechanistically understand and predict the maintenance of diversity and ecosystem functioning.

## 2 Network History and Experimental Design

BugNet was established in 2021 as a grassroots initiative by Anne Kempel and Eric Allan, following the development of its experimental design and methodology. After gauging the interest of colleagues and researchers in the field of plant‐consumer interactions, we found significant enthusiasm for the network and recruited our first collaborators. We then began seeking additional collaborators and disseminated information through social media, ecological societies, and online meetings, where we presented our methodology and addressed potential questions.

BugNet has two main components: a comparative component (introduced in a separate manuscript and not covered in this paper) and an experimental component, which forms the focus of this methods paper.

### 2.1 Site Selection and Number of Sites

Experimental sites are selected to be relatively homogeneous and dominated by herbaceous or shrubby vegetation. Relatively light natural disturbances, such as fire or grazing by vertebrates, do not need to be excluded from the site, but a record of the disturbance regime, and ideally a quantification of vertebrate herbivory, is required. The criterion for site inclusion is based on the intensity and ecological impact of vertebrate herbivory rather than its origin (i.e., wild vs. domestic). Low‐intensity or extensive grazing, regardless of whether it is anthropogenic (e.g., seasonally from free‐ranging domestic animals) or natural, is acceptable, provided it does not significantly alter vegetation structure or confound the effects of the experimental treatments. Sites where a large fraction of the annual productivity is frequently removed by grazing or management (mowing) are excluded as it is unlikely that removing invertebrate herbivores or pathogens could have an impact on productivity at these sites. However, sites heavily grazed by livestock can be included if the plots are fenced. In this case, sites probably need to be mown from time to time to avoid the establishment of woody species. Further, sites with annual soil disturbance are excluded as plant community dynamics are likely to be dominated by the disturbance regime at such sites. However, old field sites recovering from past soil disturbance can be included. During the experiment, the sites are managed to maintain previous management or according to practice in the surrounding areas, that is, if the surrounding grassland is mown once or twice a year, then the experimental site should also be mown.

**FIGURE 1:**
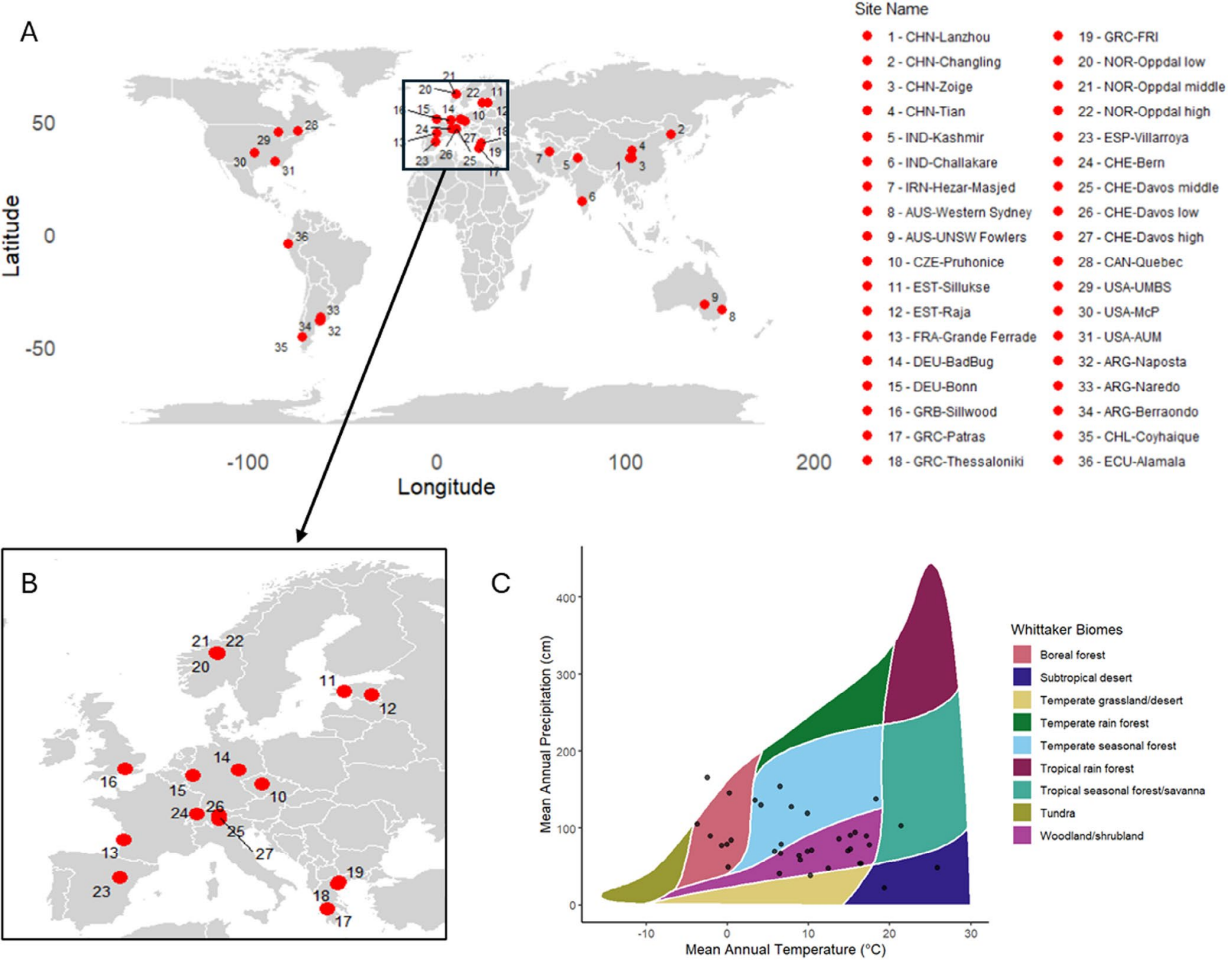
(A) Location of the current 36 BugNet experimental sites worldwide, and in (B) Europe. (C) Whittaker biome plot of site locations, showing the mean annual temperature (°C) and mean annual precipitation (cm) for all experimental sites. Climate data are taken from CHELSA and represent the average over 1980–2010 (Karger et al. 2017). The Whittaker biome plot was plotted using the plotbiomes package in R (Stefan and Levin 2018).

Currently, 36 BugNet experimental sites have been established (Figure 1A,B, see picture Gallery BugNet sites, Appendix S4). They are located in 18 different countries, in all continents except Africa and Antarctica (Asia, Europe, North America, South America and Australia), and cover a wide range of environmental conditions (Figure 1C). Of these, 20 are grasslands, six are shrublands or oldfields, nine are alpine grasslands or tundra, and one is a tropical savannah. The sites also vary in land management, ranging from entirely unmanaged areas (never mown or grazed) to sites that are lightly but regularly grazed or mown twice per year (see Table S1).

### 2.2 Treatments

**FIGURE 2:**
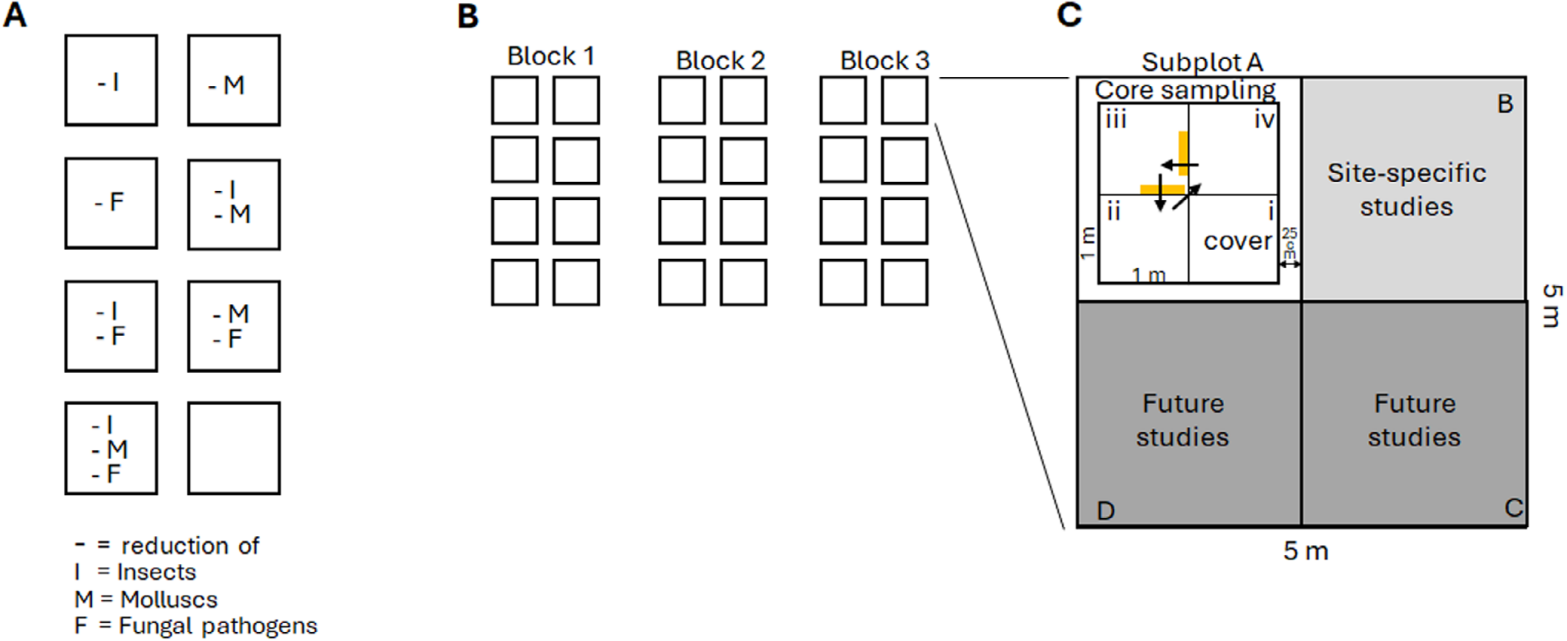
(A) Eight consumer reduction treatments have been be established in large plots (5 m × 5 m). Insects (I), mollusks (M), and fungi (F) are each reduced (−) using biocides, and the experiment contains all two‐way reductions of consumers, together with reductions of all consumer groups together and a control with no consumer reduction. (B) These eight treatment combinations are replicated across three blocks. (C) Each experimental plot is subdivided into four 2.5 m × 2.5 m subplots (A, B, C, D), one dedicated to the long‐term core sampling, one for site‐specific projects, and two for future network studies (add‐ons). The central area of the core sampling subplot is further divided into four 1 m × 1 m small plots (i, ii, iii, iv), with the one located closest to the plot centre is designated for the assessment of species composition (cover, i). The other three are designated for the biomass harvest (orange rectangles) and herbivore and pathogen damage assessment and will rotate every year.

At each experimental site, a consumer‐reduction experiment was set up to quantify the impact of three different plant consumer groups, insects, mollusks, and fungal foliar pathogens, alone and in all possible combinations (Figure 2). Each reduction experiment consists of a randomized block design with three blocks and eight treatments per block, corresponding to a full cross of the reduction of the three consumer groups, that is, each group reduced alone, all three combinations of two groups reduced, all three groups reduced, as well as a control with no reduction (*N* = 24 experimental plots per site). Each experimental plot is 5 m × 5 m in size, separated from the other plots by a 1 m walkway. Each 25 m^2^ plot is subdivided into four 2.5 m × 2.5 m subplots (A, B, C, D), with one dedicated to the core sampling, one to additional site‐specific studies, and two for future network‐level research. The subplot dedicated to the core sampling is further divided into four 1 m × 1 m small plots (i, ii, iii, iv), with the one located closest to the center designated for the assessment of species composition (cover, i). The other three small plots are designated for destructive sampling, such as the assessment of plant biomass or herbivore and pathogen damage (Figure 2).

To quantify the impact of different consumer groups, their abundance is experimentally reduced using biocides. To control insect herbivores, Lambda‐Cyhalothrin is used (e.g., Karate Zeon (active ingredient 9.43%), Syngenta). Lambda‐Cyhalothrin is a broad‐spectrum, nonsystemic insecticide frequently used in herbivore exclusion studies, which disrupts the functioning of the nervous system in insects and may cause paralysis or death. To control foliar fungi, a combination of azoxystrobin and difenoconazole is used (e.g., a mix of Score Profi (active ingredient 24.8%) and Ortiva (active ingredient 22.9%), Syngenta). Azoxystrobin inhibits fungal mitochondrial respiration, while difenoconazole interrupts the synthesis of ergosterol, a fungal cell membrane component. To control mollusks, molluscicide pellets based on ferric phosphate are applied (e.g., Limax Ferro (active ingredient ca. 1%), Syngenta). Ferric phosphate damages the digestive tissue of mollusks. In Chile and Argentina, molluscicide based on ferric phosphate was not available, and molluscicide based on metaldehyde was used. As BugNet aims to expand globally, we are aware that similar regional restrictions or product availability issues may arise. We therefore work closely with local researchers to evaluate biocide availability in each region and, when necessary, identify comparable alternatives that maintain the integrity of the experimental design. All deviations are documented to ensure transparency and facilitate future assessments of potential effects on cross‐site comparability. All biocides are applied every 4–6 weeks during the growing period (ca. four times per year on average, less frequently in areas with a shorter growing period, more often in areas with a longer growing period). While biocides may not wipe out infestation, they do significantly reduce consumer attack on plants and are the most effective experimental approach to assess the importance of invertebrate herbivores and the only approach to assess the importance of pathogens in natural plant communities (Paul et al. 1989). Insect and mollusk herbivores can be reduced using cages and fine mesh netting (e.g., Risch et al. 2018) but nets and cages are impractical for this type of experiment. Nets are difficult to construct for large plots and, in addition, a full cross of insect and mollusk exclusion would likely be impossible as no cage could be built to exclude insects but allow mollusks access. Finally, pathogens cannot be excluded using cages, and only biocides are feasible to reduce pathogen abundance in outdoor settings. All collaborators secured any permits required to use biocides and do research in their respective study locations. The biocides have all been used in previous reduction studies and have been shown to have few detectable nontarget effects (Allan et al. 2010; Allan and Crawley 2011; Bell et al. 2006; Borer et al. 2015; Cappelli et al. 2020; Maron et al. 2011; Seabloom et al. 2017). Nevertheless, nontarget effects are possible (Gandara et al. 2024; Meidl et al. 2024), for example, insecticides may affect nontarget pollinators, potentially influencing plant reproductive success in systems where plant reproduction is pollinator‐limited (Wan et al. 2025). Similarly, fungicides may reduce beneficial mycorrhizal fungi, perhaps particularly in nutrient‐limited systems, potentially confounding the interpretation of plant responses to fungal pathogen suppression (Wan et al. 2025). All biocides could also affect plant growth directly; most studies testing for such effects have not found them, but most of these have explored plants in temperate grasslands (Cappelli et al. 2020; Hector et al. 2004). We will therefore explore nontarget effects of biocides on plants, other organisms such as decomposers, and soil ecosystem functions in greenhouse experiments.

### 2.3 Measurements of Core Variables

#### 2.3.1 Baseline Measurement

To characterize the different experimental sites around the globe, soil cores are collected to assess a range of soil characteristics prior to the application of the treatments. This allows us to link consumer impact to several drivers (latitude, elevation, soil nutrient content), and to shed light on the context dependency of biotic interactions. In each of the 24 plots, two soil cores (soil corer 2.5‐cm diameter, 10‐cm depth) are collected and homogenized into a single sample per site. Soils are sieved through a 2 mm mesh, air‐dried, and sent to the project coordinators where a few key soil characteristics are measured (total organic C, total N and P stocks, and pH).

#### 2.3.2 Annual Measurements per Plot

Every year, at peak biomass, several measurements are taken per plot. The timing of peak biomass varies between sites and is defined by local researchers for their system. *Cover for each plant species* rooted within the plot is visually estimated in each of the 24 plots, in the small plot “i” (the one closest to the centre) in the core sampling subplot (see Figure 2), to the nearest 1% (up to 20% cover) and the nearest 5% for cover 20%–100%. For very rare species with less than 1% cover, 0.1% or 0.5% is assigned. In addition, the percentage cover of woody over‐storey, bryophytes, lichens, litter, bare soil, and bare rocks, if present, is estimated. Total cover typically exceeds 100% because species cover is estimated independently for each species and plant parts can overlap. In systems in which species composition shifts strongly within the year or which have a two‐times mowing regime, species composition is assessed twice, that is, before each mowing event. This allows us to account for differences in phenology and to capture the maximum cover of each species. *Aboveground biomass* is assessed by clipping the aboveground plant material to 2 cm aboveground level, in two 10 cm × 50 cm strips in one of the small plots ii, iii, or iv, of the core sampling subplot (Figure 2). The biomass is harvested in a different small plot every year, which is important to reduce disturbance to the vegetation in slow‐growing systems without regular mowing or grazing (see Figure 2). If a site has a two‐times mowing regime, biomass is collected twice per year (before each mowing event) to better estimate site productivity. Biomass is dried and weighed and is sent to the coordinators where it will be archived and used for future analysis of changes in plant nutrient and chemical composition over time. A specific protocol for shrubs is provided on the website (bug‐net.org) and in the Appendix S1.

#### 2.3.3 One‐Time Measurements

At each site, several *plant traits* are measured once, at the species level, to characterize the plant communities. These are plant height, specific leaf area (SLA) and leaf dry matter content (LDMC), which are closely associated with two major axes of plant functional variation: the size of plants and their resource economics spectrum (Díaz et al. 2016; Wright et al. 2004). Traits are measured according to protocols in Garnier et al. (2001). For each plant species present at a site, five individuals per site (without herbivore or pathogen damage) are randomly sampled in or outside experimental plots, and their height, SLA, and LDMC are assessed. Information on species traits will allow us to test whether the response of plants to consumer exclusion follows patterns predicted by defense‐deployment strategies (e.g., growth defense‐trade‐off, Table 1). At year three or four of the experiment, *herbivore and pathogen damage* (i.e., disease symptoms) is measured in each plot to assess the effectiveness of the treatments, assess interacting effects, and identify drivers of variation in damage. Damage is measured on five selected plant species per site. These species are common at a site and, ideally, present in every plot. They should also have a high cover within each plot so that the community‐weighted mean damage calculated from these five species represents approximately 80% of the total relative plant cover per plot. This approach allows us to measure community‐weighted mean damage but also to assess variation in damage at the species level. Optionally, collaborators can measure damage on more than five species and also include species that are less abundant and rarer at a site. Per species, five individuals will be randomly selected in each plot. The selection of individuals (or branches, stems or shoots in clonal plants) follows a detailed protocol to assure that individuals are indeed selected randomly and that there is no bias toward particularly damaged or undamaged individuals (described in Appendix S2). On the five individuals per species and plot, percentage damage, that is, the leaf area (%) that has been damaged, will be estimated. Percentage damage is either directly assessed in the field, or plants are brought in a cooler to a lab, and percentage damage is assessed there. In any case, five randomly selected, mature, and nonsenescing leaves per individual are visually surveyed for damage or disease symptoms. On each of the five leaves, we estimate the leaf area (%) that has been removed by chewing herbivores, showed signs of mining, galling, and sucking or rasping herbivores, and the leaf area that is covered by pathogenic disease symptoms of the categories downy mildews, powdery mildews, rusts, and leaf spots. Some plant species have fewer than five leaves, and in these cases, all available leaves are surveyed. Detailed information on how to estimate percentage damage is explained in Appendix S2. Several *other measurements* are scheduled to be taken in later years of the experiment, such as below‐ground biomass (root biomass) or measurements to characterize the invertebrate communities per plot.

### 2.4 Data Storage

Collaborators collect data and send datasheets to the project coordinators on an annual basis. In addition, collaborators send their plot biomass to the coordinators, where it is archived and used for future analysis to track potential changes in plant chemical and nutritional composition in response to aboveground consumer suppression.

### 2.5 Outline of Planned Analyses

To answer the main questions of BugNet, we will run two main linear (mixed effect) models. To assess the main and interacting effects of aboveground consumers on plant biomass, plant diversity, and functional groups, we will use plant aboveground biomass, plant species richness (or other diversity index such as Shannon diversity) and measures of plant community composition (e.g., percentage of herbs, grasses or legumes) as response variables. Below, we outline the basic models that we will run in order to test the main hypotheses; this is to ensure that different analyses, either with data from single sites or data across sites, use a standardized approach. We will run models for each site (A) and models across sites and years (B) to test for site‐specific and general patterns. Below is the R code for a normally distributed response variable; the same structure applies for nonnormal data (e.g., percentage damage), with appropriate model types (e.g., glmmtmb) and distributions:

- M_site ← lm(response~block + insecticide × molluscicide × fungicide, data = site_data)
  or M_site ← lmer(response~block + year_since_start × insecticide × fungicide × molluscicide + (1|calendar_year) + (1|plot), data = site_data)
- M_overall ← lmer(response~year_since_start × insecticide × fungicide × molluscicide + (1|site_id) + (1|site_block) + (1|site_plot) + (1|calendar_year:site), data = all_data)

Years_since_start is coded as a continuous fixed effect (years since the start of the experiment, that is, starting with 0 for the baseline data), to test for continuous changes in effects over time and to correct for the fact that experiments in different sites started in different years and have been running for different amounts of time. Calendar year is included as a categorical random effect, in Model B in interaction with site, to correct for year specific effects (e.g., a drought) which may occur within a site. We also add random effects for site and block within site (to correct for overall variation between sites and blocks) and plot within site (in Models with year_since_start, to correct for multiple measures per plot over time).

We will also run alternative models using the number of excluded groups as an explanatory variable (0, 1, 2, 3 consumer groups reduced) instead of fitting the three‐way interaction between the reduction of each consumer group.

To test for context‐dependency in consumer impact on plant biomass, plant diversity, and functional groups, we will include variables that can explain variation in consumer effects between sites. We will include hypothesized drivers of context‐dependency (Table 1): c*limatic variables* (e.g., mean annual temperature, mean annual precipitation, mean temperature during the growing season of a region, mean precipitation during the growing season of a region) averaged across standard periods (e.g., 1981–2010), s*oil parameters* (e.g., organic C, total N and P stock, C:N ratio, pH at a site level), *plant species richness* (mean species richness per m^2^ of control plots per site across years, as an indicator of species richness in the respective site) and *plant productivity* (mean biomass per m^2^ of control plots per site across years, as an indicator of plant productivity in the respective site). We will run models where the different drivers of context dependency interact with the treatments (or the number of excluded consumer groups) as follows:

C M_context ← lmer (response ~ (driver of context dependency + year_since_start) × insecticide × fungicide × molluscicide + (1|site_id) + (1|site_block) + (1|site_plot) + (1|calender_year:site), data = alldata)

We will simplify models by first removing the highest order interaction and testing whether this significantly reduces model likelihood by means of a log‐likelihood‐ratio test (comparing a model with and without the highest order interaction), if not we will stepwise delete further nonsignificant interactions until only significant interactions remain. This approach allows us to clearly evaluate interactions, however, we will also report full models (with scaled predictor variables). We will always keep the main factors in the model.

We will also include grazing intensity by vertebrates as a covariate in our models to account for potential confounding effects on plant community responses. This will help us disentangle the relative contributions of invertebrate herbivores, fungal pathogens, and vertebrate grazers to vegetation dynamics. Furthermore, we plan to expand the analyses to consider a wider range of consumer effects. We will consider other impacts on plant communities, for example, analyses testing whether community‐weighted mean traits shift in response to treatments, whether and how individual plant species or species groups, their traits or damage patterns respond to plant consumer reduction. In addition, the experiments provide an ideal platform to test for consumer impacts on ecosystem functions, such as soil biogeochemical cycling.

In order to further explore mechanisms underlying the treatment effects, we will fit structural equation models (Grace et al. 2010). These will contain the treatments as exogenous binary variables and the above‐mentioned response variables (plant species richness, plant productivity, etc.) as endogenous variables. We can then include additional variables to explain the mechanism behind impacts. For instance, we could test whether the effects of consumers on decomposition rates are mediated via changes in plant diversity, litter quality traits (e.g., specific leaf area, leaf dry matter content) or leaf nutrients (e.g., nitrogen, phosphorus).

A structural equation model could also be used to explore mechanisms underlying interactions. Interactions between consumers could arise because one group responds to another; for instance, insect herbivores may prefer to eat pathogen‐infected leaves and might therefore be reduced by pathogen exclusion, leading to an indirect effect of pathogens on the plant community by increasing insect abundance. This could be tested by including the fungicide effect on insect abundance in the structural equation model. Interactions can also be tested using multigroup structural equation models to explicitly test for variation in effects between groups, for example, variation in the effect of mollusks on herb cover in the presence or absence of insects. We will use the linear mixed models to first test for all potential interactions and will then explore those interactions that are significant in the structural equation models. We will aim to fit covariance‐based structural equation models where possible (e.g., using lavaan in R); however, if the models require complex random effect structures, the piecewise structural equation models can be used.

### 2.6 Power Analysis

To assess the power of our experimental design to detect significant treatment effects, we created a simulated dataset that mirrors the structure of our experimental setup. This simulation included 35 sites, each containing 24 plots grouped into 3 blocks, with insecticide, fungicide, and molluscicide treatments applied in a full factorial manner. We created a simulated dataset for aboveground plant biomass, with true biological variation between sites (standard deviation 372.7 g), between blocks within a site (7.82 g), and between plots within a block (15.33 g), which was based on baseline biomass data. We then simulated biomass data to evaluate the power of the experiment to detect (1) main treatment effects, (2) superadditive (synergistic) interaction effects, and (3) compensatory (antagonistic) interaction effects of different magnitudes, helping us understand which effect sizes are likely to be detected with our experimental setup (Code see Appendix S3).

1. *Main effects*. First, we simulated main effects only, testing the power to detect a 5%, 7.5%, 10%, 15%, or 20% increase in biomass for each individual treatment (insecticide, fungicide, and molluscicide). These simulations were designed to assess the sensitivity of the model to detect increases in biomass resulting from the application of insecticide, fungicide, or molluscicide in isolation.
2. *Superadditive interaction effects*. Next, we simulated superadditive (synergistic) interaction effects, where the combined application of two treatments (e.g., fungicide and molluscicide) results in a biomass increase that exceeds the individual main effects. We simulated main effects of 5%, 7.5%, 10%, 15%, and 20%, along with additional increases of 5%, 7.5%, 10%, 15% and 20%, respectively, in biomass for the combined application.
3. *Compensatory interaction effects*. Finally, we simulated compensatory (antagonistic) interaction effects, where the application of two biocides together results in no increase in biomass despite each biocide individually increasing biomass. We simulated compensatory effects with main effects of 5%, 10%, and 20%, while assuming that the combined application of two biocides would show no increase in biomass, that is, perfect compensation for each consumer group by the other.

We do not attempt to simulate changes in effects over time as well as instead assume that data from a single time point, for example, 3 years from the start at each site. Biomass for each plot was simulated using the following model:

$$\begin{array}{c} \text{biomass}=\beta_{0}+\beta_{i}\times \text{insecticide}+\beta_{f}\times \text{fungicide}\\ +\beta_{m}\times \text{molluscicide}+\beta_{f:m}\times \left(\text{fungicide}\times \text{molluscicide}\right)\\ +\beta_{i:m}\times \left(\text{insecticide}\times \text{molluscicide}\right)+\beta_{i:f}\times \left(\text{insecticide}\times \text{fungicide}\right)\\ +\beta_{i:f:m}\times \left(\text{insecticide}\times \text{fungicide}\times \text{molluscicide}\right)+\text{site effect}\\ +\text{block effect}+\text{plot effect} \end{array}$$

where *β*_0_ is a baseline biomass (100 g), *β*_i_, *β*_f_, and *β*_m_ are the main effects of insecticide (which vary depending on the effect size simulated), fungicide, and molluscicide treatments (coded as 0, 1), respectively, *β*_f:m_, *β*_i:m_, *β*_i:f_, and *β*_i:f:m_ are the interaction effects, and site, block, and plot effects account for random variation at the site, block, and plot levels, based on their respective standard deviations.

### 2.7 Power Analysis Test

The biomass response for each plot was modeled as a function of the fixed treatment effects (insecticide, fungicide, and molluscicide) and random effects for site and block. To assess the statistical power of the models, we conducted different power analyses using the powerSim function from the simr package (Green and MacLeod 2016), and 300 simulations. We tested the different scenarios described above, varying the effect sizes, to estimate the power to detect each treatment effect.

The power to detect *main effects* of a 5% increase in biomass following treatment application was low (33.7%); however, main effects of 7.5%–20% reached a power of 73.3%–100%, indicating a high likelihood of detecting a statistically significant effect. *Superadditive effects*, where the combined impact of treatments exceeds the sum of their individual effects, are likely to be detected if they reach a magnitude of at least 10% (10% increase in biomass of main effects plus an additional increase of 10% when treatments are applied in combination). *Compensatory effects*, where the combined effect of two treatments is less than the sum of their individual effects, will be detected with a high certainty even if the increase in biomass of main effects is only 5% (see Table 2).

**TABLE 2:** Summary of the statistical power to detect treatment effects across different scenarios: Main effects, superadditive effects, and compensatory effects. Power estimates are reported for 5 effect sizes (20%, 15%, 10%, 7.5%, and 5% increases in biomass). In the superadditive effects scenario, an increase of, for example, 20% refers to a 20% increase from each individual treatment plus an additional 20% due to the superadditive effect, resulting in a total 60% increase when both treatments are applied together compared to the control. For compensatory effects, an increase of 20% indicates a 20% increase from each individual treatment, but no additional increase when both treatments are combined. Each power estimate is presented with its corresponding 95% confidence interval.

Effect type	Effect size	Power estimate (%)	95% Confidence interval
Main effects	20% increase	100	98.78, 100.00
	15% increase	100	98.78, 100.00
	10% increase	97.33	94.81, 98.84
	7.5% increase	73.33	67.95, 78.25
	5% increase	33.67	28.34, 39.32
Superadditive interaction	20% increase	100	98.78% 100.00
	15% increase	99.67	98.16, 99.99
	10% increase	72.00	66.55, 77.01
	7.5% increase	41.00	35.38, 46.80
	5% increase	13.67	9.99, 18.08
Compensatory interaction	20% increase	100	98.78, 100.00
	15% increase	100	98.78, 100.00
	10% increase	100	98.78, 100.00
	7.5% increase	100	98.78, 100.00
	5% increase	97.67	95.25, 99.06

### 2.8 Analysis of False Positives

In complex factorial experiments, multiple hypothesis tests increase the risk of detecting spurious significant effects due to random variation rather than true biological patterns. To quantify this risk and assess the robustness of our statistical approach, we conducted a permutation‐based false positive analysis. By shuffling biomass values within blocks while preserving the experimental structure, we estimated how often main effects and interactions would be significant purely by chance (*p* < 0.05). This allows us to evaluate the reliability of our statistical results and provides a benchmark against which to interpret observed significant effects in the real dataset. Specifically, we randomized the most recent biomass data per site (data from 29 sites) within blocks, ensuring that the experimental design remained intact. For each iteration, we fitted the following linear mixed‐effects model in R:

$$\begin{array}{c} M\leftarrow \text{lmer}(\log\_\text{transformed biomass}\sim i^{*}f^{*}m\\ \left.+\left(1|\text{site}\_\mathrm{id}\right)+\left(1|\text{site}\_\text{block}\right),\text{data}=\text{shuffled}\_\text{data}\right) \end{array}$$

where i (insecticide), m (molluscicide), and f (fungicide) represent the experimental treatments, and *site_id* and *site_block* were included as a random intercept to account for site‐level variation, respectively variation between blocks within a site. The biocide treatments were coded as −1 and +1, following Schielzeth (2010), to facilitate interpretation of main effects from the model summary. This process was repeated 10,000 times, each time shuffling biomass values within blocks without replacement and refitting the model. We then recorded the number of times each main effect and interaction term was found to be statistically significant (*p* < 0.05).

Our false positive analysis, based on 10,000 permutations, detected significant effects (*p* < 0.05) in 448 to 542 cases, depending on the specific term (Table 3). This corresponds to a false positive rate of approximately 4.48%–5.42%, which is at the expected 5% significance threshold under the null hypothesis. The false positive analysis confirmed that our statistical approach is well‐calibrated and that any significant findings will not be overly influenced by random chance, increasing confidence that most observed significant effects in the real dataset reflect meaningful biological patterns rather than statistical artifacts.

**TABLE 3:** Results of a false positive analysis. In 10,000 iterations, significant effects for interactions and main effects (*p* < 0.05) were detected in 448 to 542 cases.

Term	Total iterations	Significant count	Proportion of significant effects
Intercept	10,000	10,000	1.00
Fungicide (F)	10,000	503	0.0503
Insecticide (I)	10,000	500	0.05
Molluscicide (M)	10,000	522	0.0522
F × M	10,000	522	0.0522
I × M	10,000	524	0.0524
I × F	10,000	484	0.0484
I × F × M	10,000	542	0.0542

## 3 Network Characteristics and Outlook

### 3.1 Network Structure and Collaborators

Currently, the experimental part of BugNet includes 36 experimental sites and involves the participation of 77 researchers from 18 countries. There are still areas with no or only low coverage of experimental sites, such as Africa, Central Asia, Western North America, Western Oceania, Eastern South America, Russia, and the Middle East (Figure 1A), and we invite researchers from these regions in particular to participate in the network by establishing an experimental site.

**FIGURE 3:**
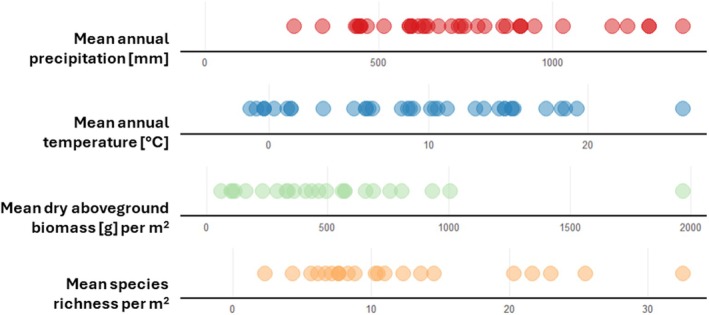
Spread of the mean annual precipitation, mean annual temperature, mean aboveground dry biomass per m^2^, and mean plant species richness of control plots (size 1 m^2^) per experimental site.

BugNet sites differ in climatic variables, soil nutrient content, and also in characteristics of their plant communities (Figure 3). For example, mean aboveground dry biomass per m^2^ varies from 62.1–1966.4 g, and mean species richness per plot (1 m^2^) varies from 2.33 species to 32.55 species. This substantial variation across sites is promising and indicates that the network is well positioned to detect context‐dependent consumer effects and to address broad‐scale ecological questions once experimental treatments progress.

BugNet is guided by an *advisory board comprising* experts in plant‐pathogen and plant‐herbivore interactions, with extensive experience in both experimental and observational ecology (Anna‐Lisa Laine—University of Helsinki (Finnland), Anne Ebeling—University of Jena (Germany), Harald Auge—Helmholtz Centre for Environmental Research (Germany), Michael Crawley—Imperial College London (United Kingdom), Nathan Sanders—University of Michigan (USA)).

### 3.2 Network Related Add‐Ons

One strength of global collaborative research projects such as BugNet is that collaborators have the opportunity to propose new measurements and lead additional studies beyond the core ideas of BugNet (Measurement add‐ons). In addition, the network offers the possibility to address additional questions through the application of additional treatments (Treatment add‐ons). In network meetings, collaborators decided on several add‐on treatments, which will be applied to the subplots dedicated to future network‐related activities. The network is currently running or has run several additional sampling campaigns of measurement add‐ons, and one treatment add‐on, summarized in Table 4.

**TABLE 4:** Summary of ongoing or past measurement add‐ons (colored icons), and the treatment add‐on (black) within BugNet.

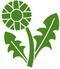	Phytometer add‐on	Phytometers (standard plants) were planted into experimental plots to assess large‐scale variation and interacting effects in consumer impact and damage on common plant species (same seed material of *Trifolium pratense, Dactylis glomerata, Taraxacum officinale*). *Performed in 2023/2024*.
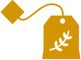	Teabag add‐on	By conducting decomposition experiments within each experimental site, where teabags are placed in the soil, we can evaluate the influence of various aboveground consumers on decomposition processes. *Performed in 2024/2025*.
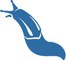	Mollusk add‐on	Mollusk abundance, biomass and identity were surveyed in experimental plots to assess the efficiency of the treatments and potential effects of insects and foliar pathogens on mollusk abundance. *Performed in 2024*.
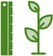	Allometry add‐on	Height and biomass of individual plants of several species and light intensity are measured in experimental plots to test whether the removal of consumers changes plant allometry by decreasing light. *Ongoing*.
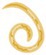	Nematode add‐on	Nematode communities will be investigated in this add‐on to assess the effects of aboveground consumer removal on nematode communities, which are important indicators of soil functioning and health. *Will start in 2025*.
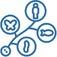	Evolution add‐on	Leaves and seeds of a few plant species are collected at the start of the experiment, prior treatment application. In the future, this allows us to test whether plants undergo evolutionary changes and allocate resources away from defense. It might also allow us to test whether the strength of evolution is context dependent. *Ongoing*.
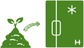	Soil storing add‐on	Prior to treatment application, several collaborators collected soil per plot and stored it in freezers. This will allow us to test for changes in the microbial communities in response to the consumer removal treatments. *Ongoing*.
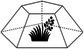	Warming treatment add‐on	Open‐top‐chambers ‐ small greenhouse‐like structures that warm the vegetation—are installed within the experimental plots to test how warming affects the impact of plant consumers. *Ongoing*.

## 4 Conclusion

The Bug‐Network has a large potential to uncover general patterns in the role of various plant consumers in shaping plant communities and ecosystem functioning. It provides an opportunity to test long‐standing ecological theory about the importance of consumers, their interacting effects, and the factors driving variation in biotic interactions. BugNet is designed as a long‐term experiment that will continue for many years, as the effects of consumer reduction on plant communities require time to manifest (Agrawal and Maron 2022). We therefore welcome researchers worldwide to join BugNet by setting up an experimental site in their region. Ultimately, BugNet aims to develop a predictive understanding of how consumers influence plant communities, and why and how antagonistic interactions vary across spatial scales—which is a pressing priority given current global change scenarios.

## Author Contributions

**Anne Kempel:** conceptualization (lead), data curation (lead), formal analysis (lead), funding acquisition (lead), investigation (lead), methodology (lead), project administration (lead), visualization (lead), writing – original draft (lead), writing – review and editing (lead). **George C. Adamidis:** data curation (equal), investigation (equal), writing – review and editing (equal). **José D. Anadón:** data curation (equal), investigation (equal), writing – review and editing (equal). **Joe Atkinson:** data curation (equal), investigation (equal), writing – review and editing (equal). **Harald Auge:** investigation (supporting), methodology (supporting), writing – review and editing (equal). **Dimitrios Avtzis:** data curation (equal), investigation (equal), writing – review and editing (equal). **Benedicte Bachelot:** data curation (equal), investigation (equal), writing – review and editing (equal). **Maral Bashirzadeh:** data curation (equal), investigation (equal), writing – review and editing (equal). **Julien L. Bota:** data curation (equal), investigation (equal), project administration (supporting), visualization (supporting), writing – review and editing (equal). **Aimee Classen:** data curation (equal), investigation (equal), writing – review and editing (equal). **Ioannis Constantinou:** data curation (equal), investigation (equal), writing – review and editing (equal). **Mick Crawley:** data curation (equal), investigation (equal), methodology (supporting), writing – review and editing (equal). **Tonia de Bellis:** data curation (equal), investigation (equal), writing – review and editing (equal). **Petr Dostal:** data curation (equal), investigation (equal), writing – review and editing (equal). **Anne Ebeling:** methodology (supporting), writing – review and editing (equal). **Nico Eisenhauer:** data curation (supporting), investigation (supporting), supervision (equal), writing – review and editing (equal). **David J. Eldridge:** data curation (equal), investigation (equal), writing – review and editing (equal). **Gustavo Encina:** data curation (equal), investigation (equal), writing – review and editing (equal). **Catalina Estrada:** data curation (equal), investigation (equal), writing – review and editing (equal). **Susan Everingham:** data curation (equal), formal analysis (supporting), investigation (equal), methodology (supporting), project administration (supporting), visualization (supporting), writing – review and editing (supporting). **Nicolas Fanin:** data curation (equal), investigation (equal), writing – review and editing (equal). **Yanhao Feng:** data curation (equal), investigation (equal), writing – review and editing (equal). **Mario Gaspar:** data curation (equal), investigation (equal), writing – review and editing (equal). **Leana Gooriah:** data curation (equal), investigation (equal), writing – review and editing (equal). **Pamela Graff:** data curation (equal), investigation (equal), writing – review and editing (equal). **Elizabeth Gusmán Montalván:** data curation (equal), investigation (equal), writing – review and editing (equal). **Pamela Gusmán Montalván:** data curation (equal), investigation (equal), writing – review and editing (equal). **Tamara R. Hartke:** data curation (equal), investigation (equal), writing – review and editing (equal). **Linjia Huang:** data curation (equal), investigation (equal), writing – review and editing (equal). **Malte Jochum:** data curation (equal), investigation (equal), writing – review and editing (equal). **Karin Kaljund:** data curation (equal), investigation (equal), writing – review and editing (equal). **Ilias Karmiris:** data curation (equal), investigation (equal), writing – review and editing (equal). **Kadri Koorem:** data curation (equal), investigation (equal), writing – review and editing (equal). **Lotte Korell:** data curation (equal), investigation (equal), writing – review and editing (equal). **Anna‐Liisa Laine:** methodology (supporting), writing – review and editing (equal). **Gaëtane le Provost:** data curation (equal), investigation (equal), writing – review and editing (equal). **Jean‐Philippe Lessard:** data curation (equal), investigation (equal), writing – review and editing (equal). **Mu Liu:** data curation (equal), investigation (equal), writing – review and editing (equal). **Yanjie Liu:** data curation (equal), investigation (equal), writing – review and editing (equal). **Juan Llancabure:** data curation (equal), investigation (equal), writing – review and editing (equal). **Sidonie Loïez:** data curation (equal), investigation (equal), writing – review and editing (equal). **Alejandro Loydi:** data curation (equal), investigation (equal), writing – review and editing (equal). **Hugo Marrero:** data curation (equal), investigation (equal), writing – review and editing (equal). **Shelby Gockel McMahan:** data curation (equal), investigation (equal). **Adrián Montoya:** data curation (equal), investigation (equal). **Zuzana Münzbergová:** data curation (equal), investigation (equal), writing – review and editing (equal). **Yujie Niu:** data curation (equal), investigation (equal), writing – review and editing (equal). **David Ott:** data curation (equal), investigation (equal), writing – review and editing (equal). **Mariano Oyarzabal:** data curation (equal), investigation (equal), writing – review and editing (equal). **Maria Panitsa:** data curation (equal), investigation (equal), writing – review and editing (equal). **Effimia Papatheodorou:** data curation (equal), investigation (equal), writing – review and editing (equal). **Frida I. Piper:** data curation (equal), investigation (equal), writing – review and editing (equal). **Kersti Püssa:** data curation (equal), investigation (equal), writing – review and editing (equal). **Karin Rand:** data curation (equal), methodology (equal), writing – review and editing (equal). **Hugo Saiz:** data curation (equal), investigation (equal), writing – review and editing (equal). **Nathan J. Sanders:** data curation (equal), investigation (equal), methodology (supporting), writing – review and editing (equal). **Martin Schädler:** data curation (equal), investigation (equal), writing – review and editing (equal). **Christoph Scherber:** data curation (equal), investigation (equal), writing – review and editing (equal). **Marina Semchenko:** data curation (equal), investigation (equal), writing – review and editing (equal). **Siim‐Kaarel Sepp:** data curation (equal), investigation (equal), writing – review and editing (equal). **Manzoor Ahmad Shah:** data curation (equal), investigation (equal), writing – review and editing (equal). **Ishrat Shaheen:** data curation (equal), investigation (equal), writing – review and editing (equal). **Claudia Stein:** data curation (equal), investigation (equal), writing – review and editing (equal). **Jana Stewart:** data curation (equal), investigation (equal), writing – review and editing (equal). **Zhuangsheng Tang:** data curation (equal), investigation (equal), writing – review and editing (equal). **Georg Tschan:** data curation (equal), investigation (equal), writing – review and editing (equal). **Saskya van Nouhuys:** data curation (equal), investigation (equal), writing – review and editing (equal). **Martijn L. Vandegehuchte:** data curation (equal), investigation (equal), writing – review and editing (equal). **Millie Vernon:** data curation (equal), investigation (equal), writing – review and editing (equal). **Sonali V. R.:** data curation (equal), investigation (equal), writing – review and editing (equal). **Jianyong Wang:** data curation (equal), investigation (equal), writing – review and editing (equal). **Yao Xiao:** data curation (equal), investigation (equal), writing – review and editing (equal). **Fotios Xystrakis:** data curation (equal), investigation (equal), writing – review and editing (equal). **Jie Yang:** data curation (equal), investigation (equal), writing – review and editing (equal). **Siwei Yang:** data curation (equal), investigation (equal), writing – review and editing (equal). **Konstantina Zografou:** data curation (equal), investigation (equal), writing – review and editing (equal). **Xiang Liu:** data curation (equal), investigation (equal), writing – review and editing (equal). **Eric Allan:** conceptualization (lead), data curation (equal), formal analysis (supporting), funding acquisition (lead), investigation (lead), methodology (lead), project administration (lead), validation (supporting), visualization (supporting), writing – original draft (equal), writing – review and editing (equal).

## Conflicts of Interest

The authors declare no conflicts of interest.

## Supporting information


**Appendix S1:** ece372111‐sup‐0001‐AppendixS1.pdf.

**Appendix S2:** ece372111‐sup‐0002‐AppendixS2.pdf.

**Appendix S3:** ece372111‐sup‐0003‐AppendixS3.pdf.

**Appendix S4:** ece372111‐sup‐0004‐AppendixS4.docx.

## Data Availability

Data sharing not applicable to this article as no datasets were generated or analyzed during the current study.
