# Supplementary material for: The Bug‐Network (BugNet): A Global Experimental Network Testing the Effects of Invertebrate Herbivores and Fungal Pathogens on Plant Communities and Ecosystem Function in Open Ecosystems
Source: Ecol Evol. 2025 Oct 9;15(10):e72111. doi: 10.1002/ece3.72111 (PMC12509180; doi:10.1002/ece3.72111)
Supplement: Supplementary file 1 — Appendix S1: ece372111‐sup‐0001‐AppendixS1.pdf. [file ECE3-15-e72111-s004.pdf]

# The “Bug-Network” – Experimental part

## A global assessment of the impact of invertebrate herbivores and pathogenic fungi on plant communities and ecosystems

***Focal research questions***

1. Do insect herbivores, molluscs and fungal pathogens differ in their impact on plant communities? And do they interact with each other?
2. When do invertebrate herbivores and fungal pathogens have the strongest effects on plant communities (productivity, community composition, diversity and functioning)?

***BugNet Goals***

Global research networks can rigorously test for general patterns and mechanisms and several, such as the NutNet or Drought-Net, have led to important advances. The goal of BugNet is to survey consumer and plant communities across sites and set-up identical insect herbivore, mollusc and fungal exclusion experiments in many parts of the world.

BugNet aims to implement a cross-site study requiring minimal investment of time and resources by each investigator. It involves an experimental study to quantify plant community and ecosystem responses to invertebrate herbivores and fungal pathogens in a wide range of herbaceous-dominated ecosystems, such as desert grasslands to arctic tundra, but also heathlands or Mediterranean shrublands.

## *Protocol*

***1.1. Selection of the site***

The site should be relatively homogeneous, dominated by herbaceous or shrub vegetation. Natural disturbances, such as fire or browsing by vertebrates, do not need to be excluded from the site, but a record of the disturbance regime, and ideally a quantification of vertebrate herbivory, is required. It is preferable that the site is not heavily grazed by livestock. Grazed sites can be included if the plots are fenced, though. In this case, the site should be mown from time to time to avoid the establishment of woody species. Light grazing by livestock is fine and can remain so.

During the experiment, the site should be managed as it is common in the area, i.e. if the grassland is mown once or twice a year, then the experimental site should also be mown. In this case make sure to plan your measurements to take place at peak plant biomass (usually before the mowing event).

***1.2. Setting up the experiment***

The experiment will be a randomized block design with three blocks, 8 treatments, and three replicates per treatment (N = 24 total experimental plots, Fig. 1). Each experimental plot will be 5 x 5 m in size, separated from the other plots by a 1m walkway. Each 25m$^2$ plot will be subdivided into four 2.5 x 2.5 m subplots (A, B, C, D), with one dedicated to the core sampling, one to additional site-specific studies and two for future network-level research (e.g. exclusion of oomycetes, warming treatment, drought treatment…). The position of the treatments in the three blocks should be randomly assigned, and also the subplots should be randomly assigned to the different uses. The subplot dedicated to the core sampling will further be divided into four 1m x 1m small plots (i, ii, iii, iv), with the one located closest to the centre designated for the assessment of species composition

(cover, i). The other three small plots will be designated for destructive sampling such as the assessment of plant biomass, or herbivore and pathogen damage (only in year three, see Fig. 1).

To quantify the impact of different consumer groups, they will be excluded (reduced) using biocides. Treatments will involve the removal of consumer groups alone, i.e. insects, mollusc, and fungi, in all possible two-way combinations, all consumer groups together and a control, giving a total of 8 treatments (Fig. 1).

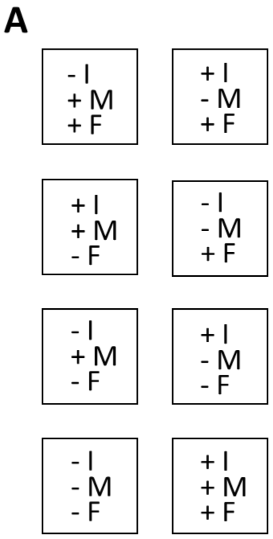


*Fig. 1: A) In 8 large plots (5 x 5m) eight consumer exclusion treatments will be established. Insects (I), molluscs (M), and fungi (F) will be excluded using biocides, as well as all two-way combinations, all consumer groups together or no consumers. B) These 8 treatment combinations will be replicated in three blocks. C) One experimental plot will be subdivided into four 2.5 x 2.5 m subplots (A, B, C, D), one dedicated to the long-term core sampling, one for site-specific projects, and two for future net-work studies. The core sampling subplot will be further divided into four 1 x 1m small plots (i, ii, iii, iv), with the one located closest to the centre designated for the assessment of species composition (cover, i). The other three will be designated for the biomass harvest (orange rectangles) and in year three for the assessment of herbivore and pathogen damage and will rotate every year.*

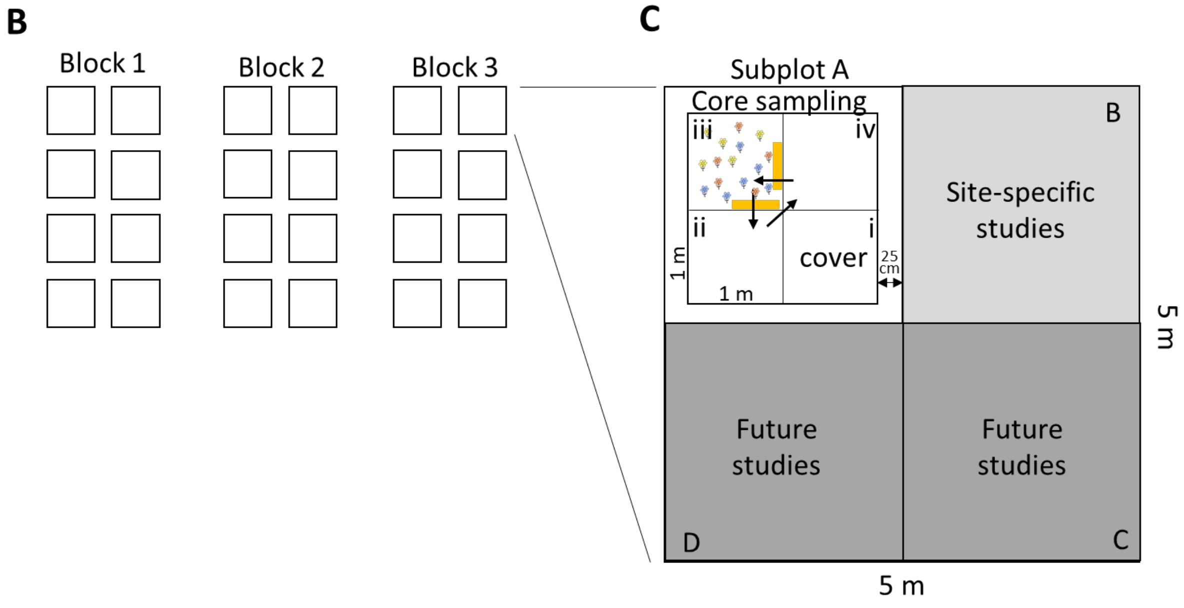


### *1.3. Treatment applications*

To control insect herbivores, we will use Lambda-Cyhalotrhin (e.g. Karate Zeon, Syngenta), which is a broad spectrum, non-systemic insecticide frequently used in herbivore exclusion studies and with few non-target effects. To control foliar fungi a combination of azoxystrobin and difenoconazole (azoxystrobin inhibits fungal mitochondrial respiration, Difenoconazole interrupts the synthesis of ergosterol, a fungal cell membrane component, e.g. a mix of Score Profi and Ortiva, Syngenta), will be used. Both pesticides will be spayed at the beginning of the growing season, and then every 4-6 weeks until the end of the growing season. To control molluscs, molluscicide pellets based on ferric

phosphate (e.g. Limax Ferro, Maag, or any other product based on ferric phosphate (e.g. Sluggo), sometimes also called iron phosphate) will be applied at the same times as the other pesticides. The number of applications will be larger in regions with a very long growing season, but this is appropriate given that the consumers are active for longer and that we want to ensure that they are effectively reduced in abundance. However, do check the local regulations in your area and make sure you do not apply pesticides more than is allowed. It might also be that in some countries some biocides are not approved. If this is the case, please contact us and we will discuss alternative products that can be used. Biocides may not wipe out infestation, but they do significantly reduce enemy attack on plants and are so far the only experimental approach to assess the importance of invertebrate herbivores and pathogens in natural plant communities. Check out our detailed protocol on pesticide application.

The current recommended dosage is for low (< 50 cm) vegetation. If you are in a productive site you should increase the dosage. Make sure to report this in your datasheet.

### *1.4. Measurements per site – Baseline data (prior to treatment application)*

To characterize the different sites around the globe, several measurements of soil conditions should be taken prior to the application of the treatments. This allows us to link consumer impact to several drivers (latitude, altitude, soil fertility), and to shed light at the context dependency of biotic interactions.

#### *Soil samples*

Soil cores will be collected to assess a range of soil characteristics. In each of the 24 plots, collect two soil cores (soil corer 2.5 x 10 cm) and homogenize the soil into a single sample per site. Please sieve the soil through a 2 mm mesh. Soils should be air-dried and send to the project coordinators (see soil processing, labelling and mailing protocol for more details). There, total organic C, total N and P stocks, as well as pH will be measured and will give information on soil characteristics at the site level.

#### *Long term soil storage (not obligatory)*

In addition, we would like to obtain information of the soil microbial community at the plot level. The soil microbial community is likely to change in response to the pesticide treatments. To be able to analyse this change at some point in the future, it is necessary to have information on pre-treatment conditions. We therefore encourage you to store soil samples **per plot** prior to the application of the treatments in a freezer. However, this is not obligatory and you can also participate in the experimental part without long term soil storage.
Please take **five soil** cores (e.g. 2.5 x 10 cm) from random locations per plot with a soil corer, and homogenize the soil into a single sample per plot. Make sure to carefully clean your soil corer between plots. We ask you to store **50g** of soil per plot in a labelled zip block bag or any other container at -20°C, and 5g of soil per plot at -80°C (if you have access to such a freezer). Don't forget to label your bags well, as they might sit in the freezer for a few years before we analyse the microbial community at some point in the future.

### *1.5. Annual measurements per plot*

***These measurements should be taken every year, at peak biomass.***

#### *Plant species composition*

Once annually, estimate the percent plant cover per plant species in each of the 24 plots, in the subplot dedicated to the core sampling, in the small plot located closest to the centre (see Fig. 1, i, cover).

Cover for each plant species rooted within the plot will be estimated to the nearest 1% (up to 20% cover) and the nearest 5% for cover 20-100%. Assign 0.1% or 0.5% to very rare species with less than 1% cover. Estimate also the percent cover for woody over storey, bryophytes, lichens, litter, bare soil, and rocks if present (see datasheet). Total cover will typically exceed 100% because species cover is estimated independently for each species.
To reduce bias in cover assessment, it is helpful to train yourself by placing differently sized pieces of paper on the plot: e.g. 10cm x 30cm = 3 %, 10cm x 10cm = 1 %, 3.1cm  x 3.2cm = 0.1%, 31cm x 32cm = 10 % …

In systems in which species composition shifts strongly within the year or which have a two-times mowing regime, we recommend that species composition is assessed twice, once in spring/early summer and once in late summer. This allows us to account for differences in phenology and to capture the maximum cover of each species.

### *Aboveground biomass*

To quantify consumer impact on productivity (top-down control), clip the aboveground plant material to 2 cm above ground level, in two 10cm x 50 cm strips of each core sampling subplot, in one of the small plots that are used for destructive sampling (ii, iii, iv). If your site is now mown, each year, the biomass harvest should be done in a different small plot (see Fig. 1).

If you work in very unproductive systems which are not mown, and in which the removal of biomass might still be visible after three years, then try to not overlap the stripes for biomass harvest for as long as possible (see example of harvest positions, Fig. 2).

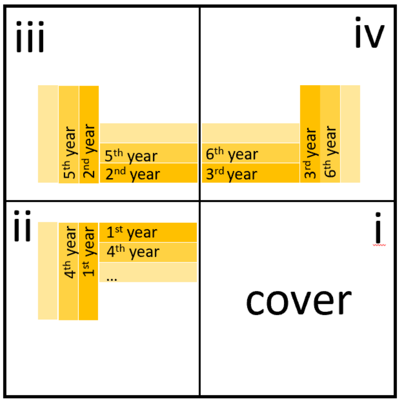


*Fig. 2: In very unproductive systems, in which plants grow very slowly and the removal of biomass might still be visible after a few years try to not overlap the stripes for biomass harvests for as much time as possible.*

Sampling should be done at peak biomass production (the timing of peak biomass will vary between sites and will be defined by local researchers for their system). If your site has a two-times mowing regime, biomass should be collected twice per year to better estimate site productivity (before the cuts). Collect the total aboveground biomass, dry and weigh it (the two subsamples per plot can be combined). Send a subsample (ca. 20 g of dry weight per plot, the two strips per plot can be combined) of the dry biomass samples to the project coordinators (see sample processing and labelling protocol) every year. If you can grind the biomass to powder, that would be ideal, but if not you can cut the biomass sample in pieces and send us a well-mixed subsample.

We will then measure several leaf characteristics (leaf N and P, fibre content etc.), and to track plant quality in response to the treatments. We might also look at the phyllosphere microbiome at some point.

If you work in shrublands the biomass of the shrub species will be estimated using allometric equations. For each shrub species, measure the height and canopy diameter of 20 individuals of contrasting sizes outside of your experimental plots. Clip, dry and weigh them. If your shrubs form big patches and individuals are difficult to isolate, just take 20 "sampling units" with known height and canopy diameter, and collect the biomass of this sampling unit instead (see datasheet). To be able to obtain a measure of shrub green vs brown biomass, please separate the leaves from the woody biomass. For some species this might work better once the leaves are dry. Now measure the height and canopy diameter of all shrubs in the subplot dedicated to the core sampling, in the small plot in which you also assess the plant cover (Fig. 1, cover, i).

### *1.6 One-time measurement of plant traits*

At each site**,** several plant traits - plant height, specific leaf area (SLA) and leaf dry matter content (LDMC) - will be measured to characterize the plant communities. These traits are closely associated to two major axis of plant functional variation, the size of plants and their parts, and the resource economics spectrum (Wright et al. 2004, Díaz et al. 2016). You will measure the traits according to protocols in Garnier et al. 2001.

Height, SLA and LDMC of all plant species at a site should be measured. This is important to test whether the response of plants to enemy exclusions follow patterns predicted by defense-deployment strategies (e.g. growth defense-tradeoff). For each plant species present at a site, five individuals per site should be randomly sampled, and their height, SLA and LDMC assessed. The height can be directly measured in the field, for SLA and LDMC pick the five randomly selected individuals, bag them in a labelled plastic bags and place them in a cooler. If possible, your individuals have > 5 leaves without any damage symptoms, as ideally the leaf traits are measured on undamaged leaves (see detailed protocol on how to measure SLA and LDMC). These measurements can be done at any point up until the fourth year.

### *1.7. Special sampling campaigns per plot and additional measurements (measurement Add-ons)*

At some point we will ask you to measure a few additional variables. For example, in year three of your experiment (third year of treatment application) we ask you to measure herbivore damage and pathogen infection in all plots (see below). In year three or four we ask you to measure standing root biomass (see below) and we plan at some point to assess the invertebrate communities per plot. Also, a few measurement-addons have already started or will do so in the future (mollusc addon, decomposition addon).

There is also the possibility to propose and lead additional measurement Add-ons yourself and we encourage you to do so (see Add-on proposal). In addition, we will hold several workshops and discussion groups where we can develop ideas together. Below is a short description of the measurements, and a link to detailed protocols.

#### *Herbivore damage and fungal infection*

In year three (third year of treatment application) we ask you to measure herbivore damage and fungal infection per plot. If your system is very productive and will be mown regularly, damage and disease will be measured in the small plot dedicated for the plant cover (small plot i). If your system is very unproductive and removing individuals for damage assessment would have strong influences on the vegetation, then measure the herbivore damage and fungal infection in one of the other small plots dedicated for destructive sampling (ii, iii, iv), or alternatively, assess damage in the cover small plot without removing individuals. You will find the detailed protocol on how to assess herbivore damage and pathogen infection here.

## Root biomass

In year three or four of the experiment we ask you to measure the standing root biomass. To do so, take a soil core of 5cm diameter, 30 cm deep, per plot, and sort to separate roots. Dry and weigh the root biomass and enter the data in your annual datasheet (see datasheet).

## Decomposition Add-on

Soon there will be a protocol to participate in a decomposition add-on using the tea-bag approach.

## Mollusc sampling Add-on

Soon there will be a protocol to participate in a mollusc survey addon.

***References***


Allan, E., and M. J. Crawley. 2011. Contrasting effects of insect and molluscan herbivores on plant diversity in a long-term field experiment. Ecology Letters 14:1246–1253.

Borer, E. T., E. M. Lind, E. J. Ogdahl, E. W. Seabloom, D. Tilman, R. A. Montgomery, and L. L. Kinkel. 2015. Food-web composition and plant diversity control foliar nutrient content and stoichiometry. Journal of Ecology 103:1432–1441.

Borer, E. T., E. W. Seabloom, D. S. Gruner, W. S. Harpole, H. Hillebrand, E. M. Lind, P. B. Adler, J. Alberti, T. M. Anderson, J. D. Bakker, L. Biederman, D. Blumenthal, C. S. Brown, L. A. Brudvig, Y. M. Buckley, M. Cadotte, C. Chu, E. E. Cleland, M. J. Crawley, P. Daleo, E. I. Damschen, K. F. Davies, N. M. DeCrappeo, G. Du, J. Firn, Y. Hautier, R. W. Heckman, A. Hector, J. HilleRisLambers, O. Iribarne, J. A. Klein, J. M. H. Knops, K. J. La Pierre, A. D. B. Leakey, W. Li, A. S. MacDougall, R. L. McCulley, B. A. Melbourne, C. E. Mitchell, J. L. Moore, B. Mortensen, L. R. O'Halloran, J. L. Orrock, J. Pascual, S. M. Prober, D. A. Pyke, A. C. Risch, M. Schuetz, M. D. Smith, C. J. Stevens, L. L. Sullivan, R. J. Williams, P. D. Wragg, J. P. Wright, and L. H. Yang. 2014. Herbivores and nutrients control grassland plant diversity via light limitation. Nature 508:517.

Díaz, S., J. Kattge, J. H. C. Cornelissen, I. J. Wright, S. Lavorel, S. Dray, B. Reu, M. Kleyer, C. Wirth, I. Colin Prentice, E. Garnier, G. Bönisch, M. Westoby, H. Poorter, P. B. Reich, A. T. Moles, J. Dickie, A. N. Gillison, A. E. Zanne, J. Chave, S. Joseph Wright, S. N. Sheremet'ev, H. Jactel, C. Baraloto, B. Cerabolini, S. Pierce, B. Shipley, D. Kirkup, F. Casanoves, J. S. Joswig, A. Günther, V. Falczuk, N. Rüger, M. D. Mahecha, and L. D. Gorné. 2016. The global spectrum of plant form and function. Nature 529:167–171.

Duffy, J. E., B. J. Cardinale, K. E. France, P. B. McIntyre, E. Thébault, and M. Loreau. 2007. The functional role of biodiversity in ecosystems: incorporating trophic complexity. Ecology Letters 10:522–538.

Garnier, E., B. Shipley, C. Roumet, and G. Laurent. 2001. A standardized protocol for the determination of specific leaf area and leaf dry matter content. Functional Ecology 15:688–695.

Jia, S., X. Wang, Z. Yuan, F. Lin, J. Ye, Z. Hao, and M. S. Luskin. 2018. Global signal of top-down control of terrestrial plant communities by herbivores. Proceedings of the National Academy of Sciences 115:6237.

Lavorel, S., K. Grigulis, S. McIntyre, N. S. G. Williams, D. Garden, J. Dorrough, S. Berman, F. Quétier, A. Thébault, and A. Bonis. 2008. Assessing functional diversity in the field – methodology matters! Functional Ecology 22:134–147.

Moles, A. T., and J. Ollerton. 2016. Is the notion that species interactions are stronger and more specialized in the tropics a zombie idea? Biotropica 48:141–145.

Seabloom, E. W., E. T. Borer, and L. L. Kinkel. 2018. No evidence for trade-offs in plant responses to consumer food web manipulations. Ecology 99:1953–1963.

Terborgh, J. W. 2015. Toward a trophic theory of species diversity. Proceedings of the National Academy of Sciences 112:11415.

Weisser, W. W., and E. Siemann. 2004. Insects and Ecosystem Function. Springer-Verlag, Berlin.

Wright, I. J., P. B. Reich, M. Westoby, D. D. Ackerly, Z. Baruch, F. Bongers, J. Cavender-Bares, T. Chapin, J. H. C. Cornelissen, M. Diemer, J. Flexas, E. Garnier, P. K. Groom, J. Gulias, K. Hikosaka, B. B. Lamont, T. Lee, W. Lee, C. Lusk, J. J. Midgley, M.-L. Navas, Ü. Niinemets, J. Oleksyn, N. Osada, H. Poorter, P. Poot, L. Prior, V. I. Pyankov, C. Roumet, S. C. Thomas, M. G. Tjoelker, E. J. Veneklaas, and R. Villar. 2004. The worldwide leaf economics spectrum. Nature 428:821–827.
