# Supplementary material for: The Bug‐Network (BugNet): A Global Experimental Network Testing the Effects of Invertebrate Herbivores and Fungal Pathogens on Plant Communities and Ecosystem Function in Open Ecosystems
Source: Ecol Evol. 2025 Oct 9;15(10):e72111. doi: 10.1002/ece3.72111 (PMC12509180; doi:10.1002/ece3.72111)
Supplement: Supplementary file 2 — Appendix S2: ece372111‐sup‐0002‐AppendixS2.pdf. [file ECE3-15-e72111-s002.pdf]

Protocol on estimating leaf herbivore damage and pathogen infection

Version 2.0, 06.04.2025 (by Anne Kempel)

This is an updated protocol on how to assess herbivore and pathogen damage in your BugNet Experimental plots. We ask you to conduct damage assessments in the third year of your experiment (i.e., the third year of pesticide application). This updated version allows for the assessment of both the community-weighted mean damage per plot and the intraspecific variation in damage. We only assess % damage and will not assess incidence anymore. At the bottom of the page, you'll find more detailed tips and tricks for assessing damage, along with a picture gallery of the most common damage types.

Where to assess damage?

If your system is very productive and will be mown regularly, damage and disease will be measured in the small plot dedicated for the plant cover (small plot i). If your system is very unproductive and removing individuals for damage assessment would have strong influences on the vegetation, then measure herbivore damage and disease in one of the other small plots dedicated for destructive sampling (ii, iii, iv), or alternatively, assess damage in the cover small plot without removing individuals.

When to assess damage?

Assess herbivore and pathogen damage at peak biomass, when you also collect your biomass samples and assess the cover of plant community.

On which species and individuals to assess damage?

Selection of species: Select ***five*** plant species per site for damage assessment. These species should be common at your site and, ideally, present in every plot. They should also have a high cover within each plot, so that the community-weighted mean damage calculated from these five species represents approximately 80% of the total relative plant cover per plot. This approach allows us to measure community weighted mean damage but also to assess variation in damage at the species level. If you cannot handle the assessment of five species per plot, get in touch with us.

Addon – rare species (optional): If you're highly motivated or have help from students, you may select additional plant species beyond the five most common ones. However, these extra species should ideally occur in all—or at least a large number of—plots, but with a lower cover. It would be interesting to explore whether subdominant plant species show similar responses to consumer reduction.

Selection of individuals per species: Randomly select five individuals per species and plot. To assure that individual selection is random we came up with the following procedure. If your species has five or less individuals, select all individuals. If your species has between 5-10 individuals per plot, mark all individuals in the 1m^2 with grill sticks numbered from 1-10. Use a random number generator (or quickly select 5 numbers in your head) to select 5 individuals (it is important that the individuals are selected randomly, and that there is no bias towards particularly damaged or undamaged ones). If your species has more than 10 individuals, divide your 1m^2 plot into four quadrants, and estimate the proportion of individuals in each of the four quadrants. Randomly select individuals per quadrant in proportion to their numbers of individuals, e.g., if quadrant 1 contains 80% of all individuals, and

quadrant 3 20%, then randomly select four individuals of quadrant 1 and one of quadrant 3. Particularly, if the distribution of your species is very patchy (e.g. one large patch with many individuals, and 3 isolated individuals) this method prevents that you will select isolated individuals with a higher likelihood (see Fig. 1).

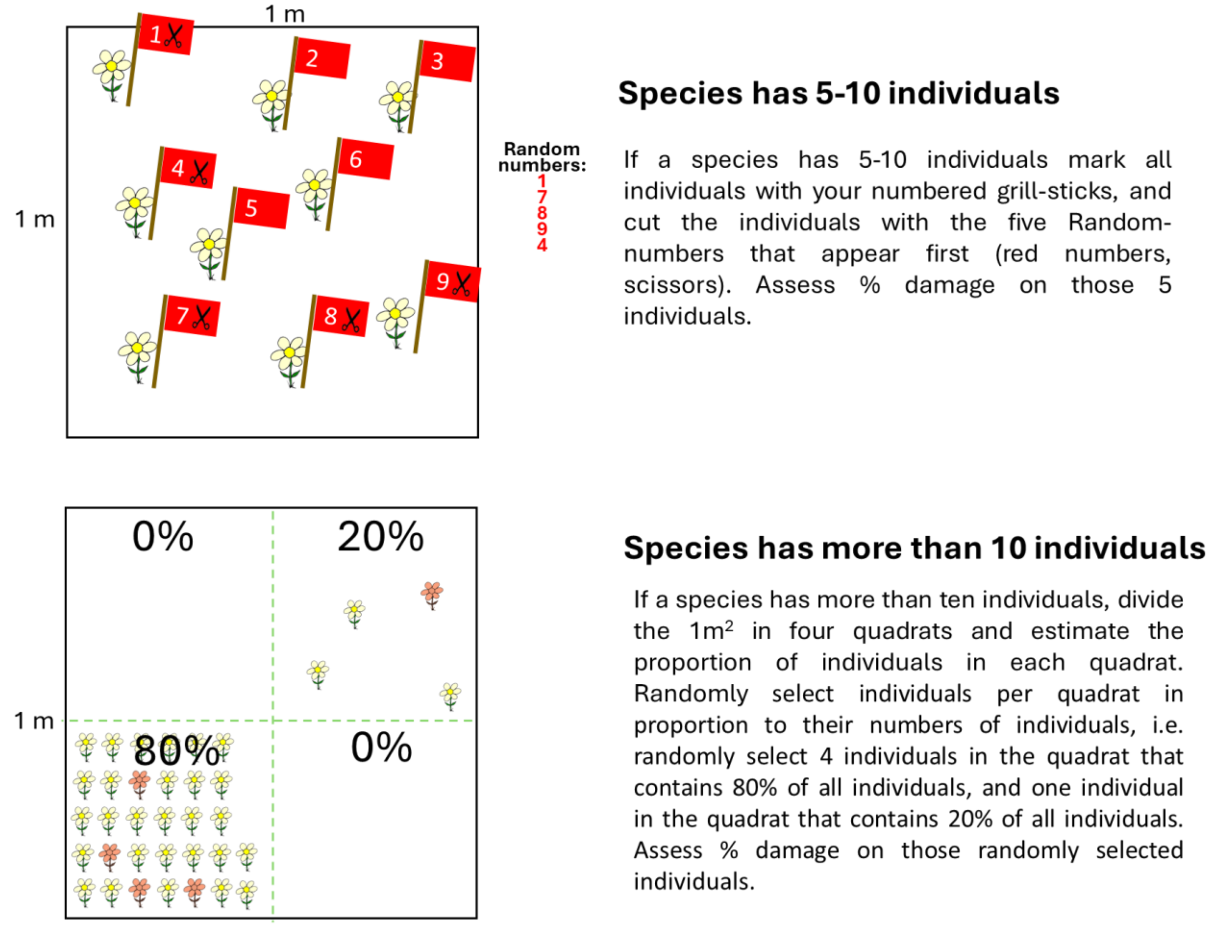

Fig. 1: *Selection of individuals per plant species. For each selected plant species,* choose five individuals on which you will assess the percentage of damage across different damage categories. You can either cut the individuals at ground level (or a subset of them), bag them, and bring them to the lab for assessment of leaf damage percentage—or carry out the damage assessment directly in the field.

How to measure?

Measurements: On each of the selected five individuals per species, estimate the **leaf area (%)** that has been **damaged.** You can either do this directly in the field, or you can bag the plants in labelled plastic bags, place them in a cooler, bring them to the lab, and do the % damage assessment there. In any case, visually survey **five *random***, mature, and non-senescing leaves (or leaflets if your leaves are very small) per individual for damage and disease symptoms. For easier inspection you can use hand lenses to better assign damage types or use a stereomicroscope. On each of the five leaves estimate the leaf area (%) that has been removed by chewing herbivores, mining, galling and sucking/rasping herbivores, and the leaf area that is covered by pathogenic disease symptoms of the categories downy mildews, powdery mildews, rusts and leaf spots (see damage gallery below). Some plant individuals

will have fewer than 5 leaves, and for these all leaves should be surveyed (but leave out senescent leaves). Note that in some cases, damage is present on only the underside of leaves, so remember to check both sides of the leaf for damage. Below you will find more tips and tricks on how to assess damage and also check our photo gallery of the most common damage types. We also highly recommend that you train your damage assessment with the ZAX Herbivory Trainer app by Zoe Xirocostas and Angela Moles!

Shrublands: If you work in shrublands, you may not find five individuals of a shrub species in your 1m^2 plot but very likely will have only one or two individuals. You may also have spreading shrubs where it can be difficult to distinguish individuals. In this case, randomly pick 25 leaves from 5 different branches per shrub species throughout the plot (5 leaves per branch). Make sure that the 5 branches are from either as many shrub individuals as possible, or else take branches from different positions and orientations within the shrub patch. Assess the % leaf area damaged per shrub species.

If your shrub species is leafless and instead has photosynthetic stems (e.g. *Retama ssp*.), instead of picking leaves, randomly cut 5 cm pieces from 5 different branches throughout the plot. On these, assess % damage either in the field or collect the pieces to assess damage in the lab.

More tips and tricks on how to assess damage

1. How can I distinguish the different damage types?

We will differentiate between the following damage types, see also our damage type gallery:

Herbivores

- *Chewing damage*: any missing area of a leaf blade, such as holes within the leaf, or cuttings at the edges, that go all the way through the leaf, including tissue removal at leaf margins. Exceptions are holes surrounded by dead, brown tissues, often fading into yellow. These are the symptoms of leaf spot disease, caused by bacterial or fungal infections which results in necrosis where dry dead tissue can break off leaving holes.
- *Sap-sucking or rasping damage:* this is a bit more difficult to identify. Sucking herbivore damage is often visible as regularly shaped, circular punctures, surrounded by lighter colored tissue. Against the light it looks like yellow, discolored spots. Irregularly shaped discolored spots or areas instead are likely to be caused by pathogens. Rasping damage leaves at least the epidermis on top or bottom intact, and causes no hole (often caused by molluscs).
- *Leaf-mining damage:* transparent traces or feeding corridors on a leaf (due to missing mesophyll tissue), where the two epidermal layers are still intact. You can often see the excrement of the mining insects in the mine. Please also count any brownish area next to the mines, as this damaged area is attributed to the mining. Mostly caused by moth (Lepidoptera), sawflies (Symphyta), flies (Diptera), sometimes beetles (Coleoptera).
- *Galling:* swelling growth on the external tissues of plants. They can be caused by various organisms, from viruses, fungi, insects and mites.

Pathogens

- *Rust fungi (Pucciniomycotina):* They are easily recognized with the yellow, red, or brown urediniospores released from little “volcanos” or “pillows”, where most spores can be brushed

away by your finger except some which look black and not easily brushed away. Rusts can be found on both sides of the leaves, so don't forget to check both sides. Often the presence of a yellow spot on the top of a leaf may indicate the presence of a rust underneath.

- *Downy mildews (Oomycetes):* obligate parasites of plants. Initial symptoms are large, angular, pale green to yellow areas which are visible on the upper surface of leaves, and later turn brown. On the undersides, these areas are covered with white to grayish, cotton-like (downy) structures, which appears watersoaked.
- *Powdery mildews (Erysiphales): white-greyish powdery* circles (mycelium) on the upper leaf surface which can easily be brushed off. Powdery mildew can also be on the backside of the leaf. When ripe, little dots can be seen at the underside of the leaf. Powerdery mildews are obligate biotroph and relies on living substrate to survive.
- *Leaf spots:* Leaf spot symptoms are discolored, sometimes reddish to brown circles of necrotic plant tissue, which often have a center of necrosis or cell death, and sometimes several rings around the center. They are caused by a different fungal pathogens, bacteria or viruses.

2. How to estimate damage?

We largely follow the protocols for damage estimation from "The Herbivory Variability Network" (HerbVar, herbvar.org), who kindly allowed us to use their description here – so most of the text has been written by members of herbvar. Thus, we recommend using visual estimation because digital methods are slower and more laborious. Also, careful visual estimations have been shown to do a surprisingly good job, especially after some practice. To increase precision in visual estimation of percentage damage we recommend that you train your damage assessment with the ZAX Herbivory Trainer app by Zoe Xirocostas and Angela Moles!

How does visual estimation work?

- *Visual estimation* is very simple. Look at a leaf and guess what percent was removed or damaged or shows pathogen symptoms. Try to estimate at a high resolution of at least 2.5 % (see Table 1). Even if your estimate has an error, a high resolution estimates will still be closer to the true value than estimates that are reported in coarse categories.
- When you first look at a leaf, do a quick mental calibration before estimating damage. For this, imagine to cut the leaf into a range of proportion. For example, think about what half the leaf would look like (50%), then imagine a quarter (25%) of the leaf. Do the same for a tenth of the leaf (10%) by mentally cutting the leaf into 10 equally-sized pieces. Then mentally cut one tenth in half to get an idea of 5% leaf area. Half of that unit would then be 2.5 %.
- When it is time to do the actual herbivory estimate, one strategy that works well for contiguous blocks of damage is to use fractional thinking, starting with larger fractions and gradually working your way down to smaller fractions – honing from a coarse estimate to a precise estimate. For example,
    - If ~12.5% of a leaf were damaged, then...
        - Mentally cut the leaf into quarters
        - See that less than a quarter (25%) is damaged
        - Mentally cut the quarter with damage in half, yielding eighths (12.5%)
        - See that the area damaged is equal to an eighth and record 12.5%
    - If ~30% of a leaf were missing, then…
        - Mentally cut the leaf in half

        - See that less than half is damaged
        - Mentally cut the leaf into quarters
        - See that more than a quarter (25%) is damaged
        - Take mental note of the 25% damaged, and then focus on estimating how much more than that 25% is damaged
        - Mentally halve the quarter of the leaf with the excess damage above 25%, yielding eighths (12.5%)
        - See that the damage above 25% is a little less than half of one of those eighths, which means it's a little less than a sixteenth or 6.25%
        - 25% plus a little less than 6.25% comes close to 30%, record it!
    - If your leaf has more than one area of damage, try mentally consolidating each area of damage into one area and then estimate the size of that using the method above. Alternatively, if mental consolidation isn't working well, you can mentally divide the leaf into fractions that are as small as the smallest patch of herbivore damage. Then simply mentally tally the number of patches of that size that would be damaged.
    - For complexly pinnate leaves (e.g., Apiaceae), it is probably best to divide the leaf into leaflets or pairs of leaflets, then follow the methods above.
    - If damage by herbivores is very high and very little leaf tissue remains, take a large and small leaf and compare the leaf base width, petiole and midrib size to compare. Use these comparisons to visually reconstruct the leaf, and deduce % damage from there
    - Through all of this, make sure you are correctly identifying what is herbivore damage versus disease versus physical damage. Please have a look at our ***Picture Gallery of plant damage***. We are trying to avoid damage caused by abiotic stress. E.g. wind damage can manifest as browning.
- Mines should be included in percent damage.
- Galls should only be counted, and not included in percent damage because galls are actually extra tissue. The removed tissue is internal and can't be seen.

Table 1: Recommended resolution for recording percent damage. Adopted from herbvar.org.

Percent	Meaning
0%	No damage
0.50%	A trace amount of damage
1%	≈ 1% damage
2.50%	≈ 2.5 % damage
5%	≈ 5% damage
7.50%	≈ 7.5% damage
So forth	Up to 100% (e.g. everything removed or infested except base of leaf petiole)

We thank again the Herbivore Variability Network (herbvar.org) for using part of their protocols!

Picture gallery of damage types

Chewing herbivore damage

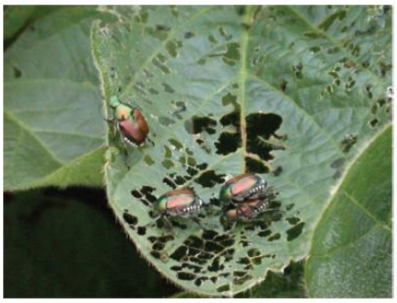

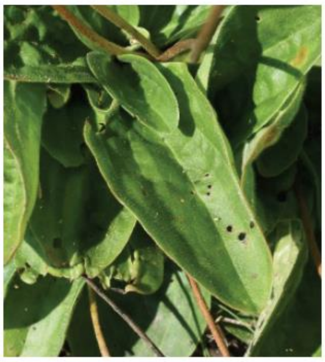

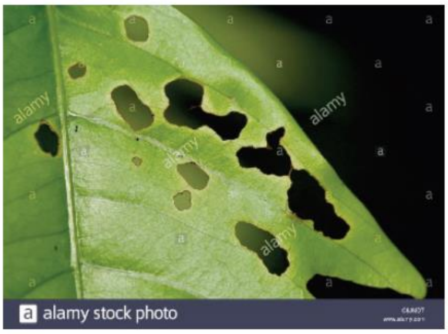

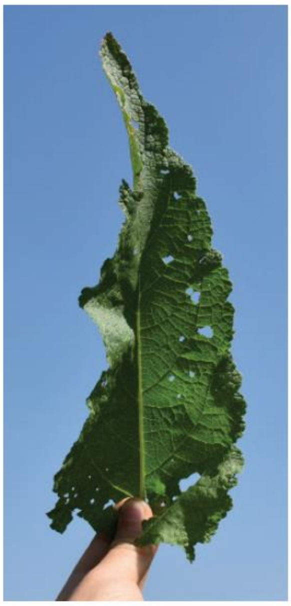

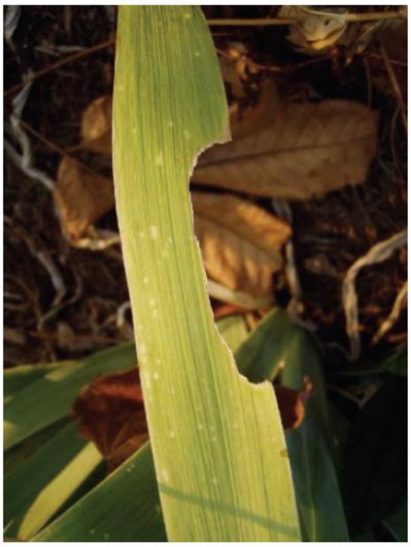

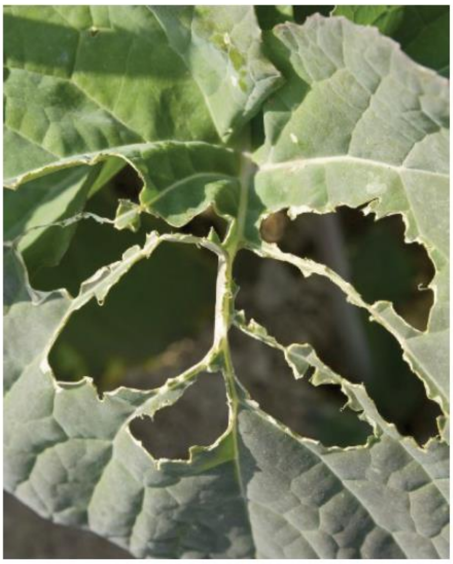

Sap-sucking or rasping herbivore damage

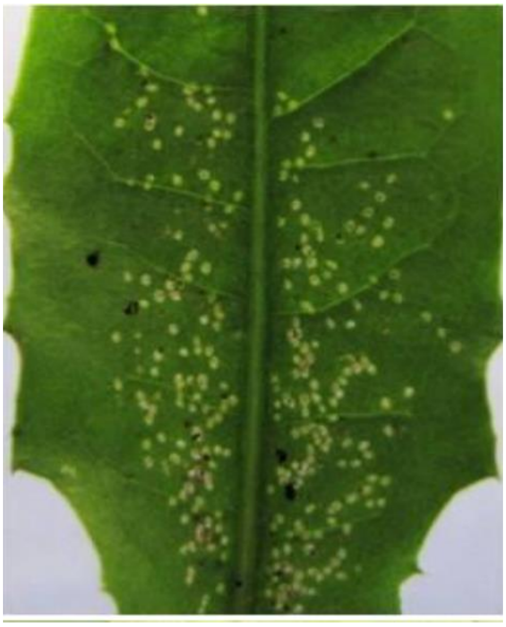

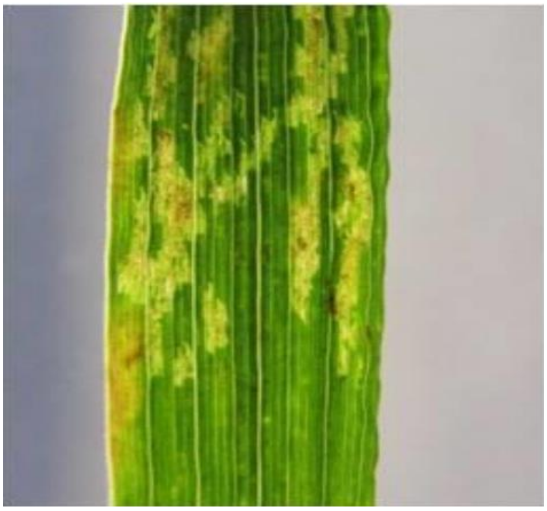

Leaf-mining damage

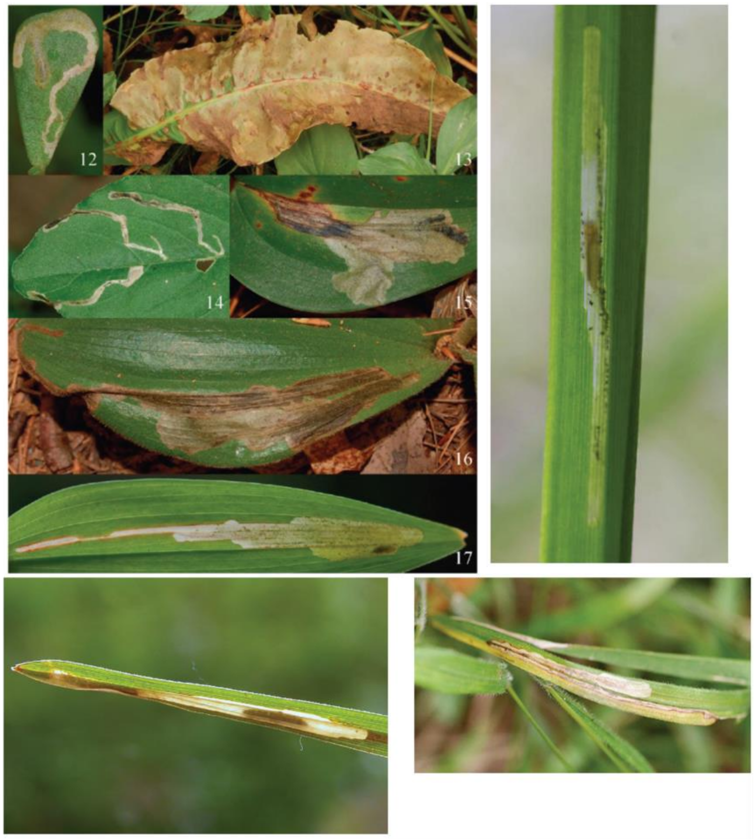

5: Pannel on leaf-mining

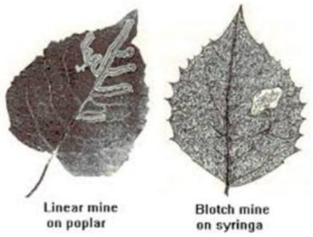

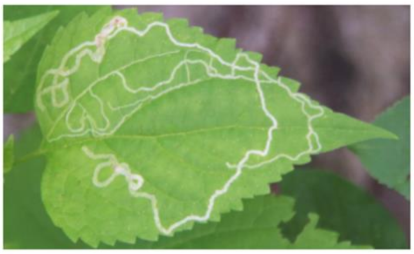

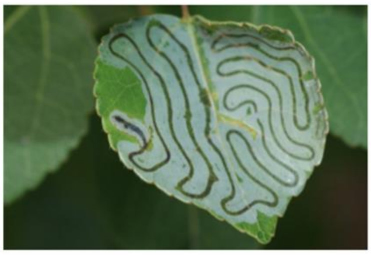

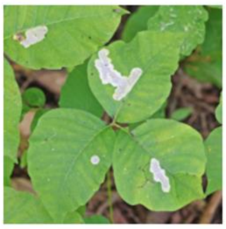

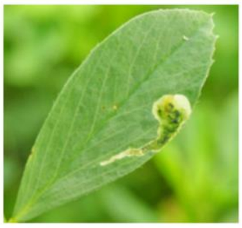

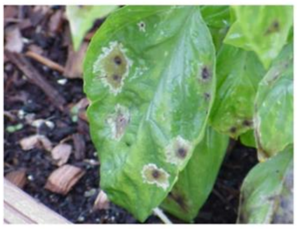

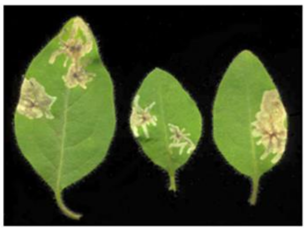

Galling

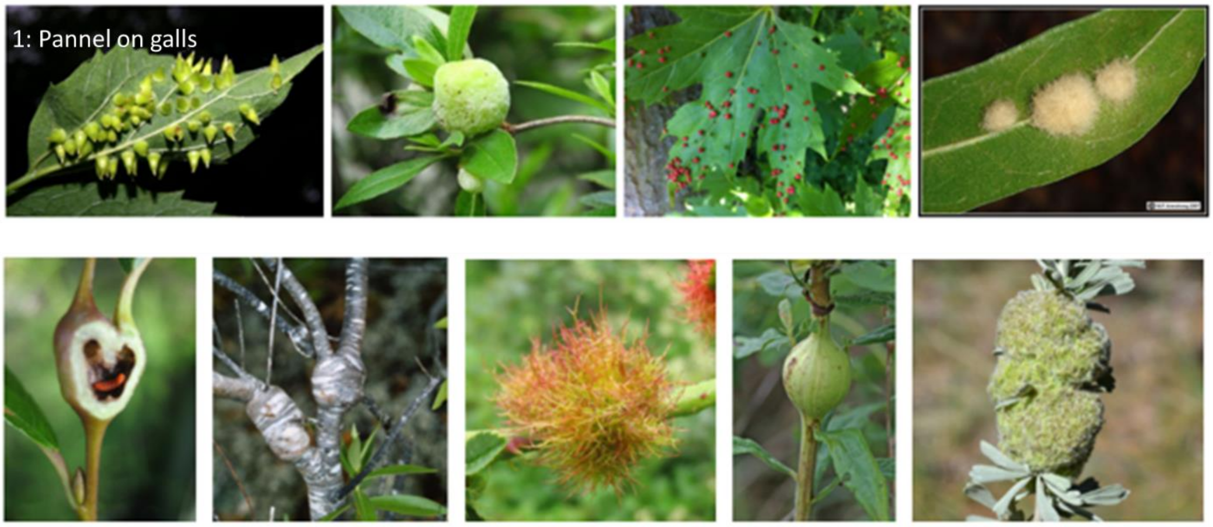

1: Pannel on galls

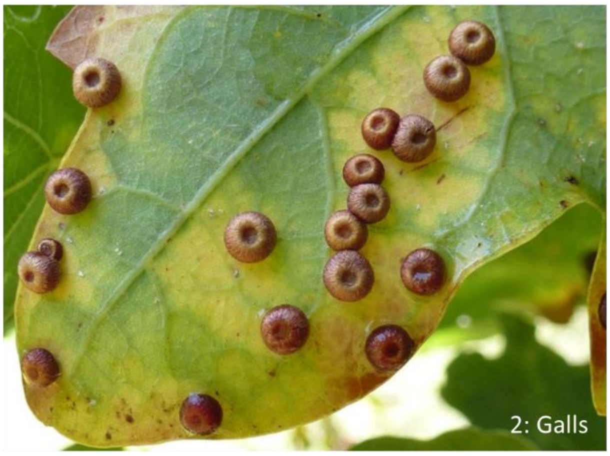

2: Galls

Rust fungi

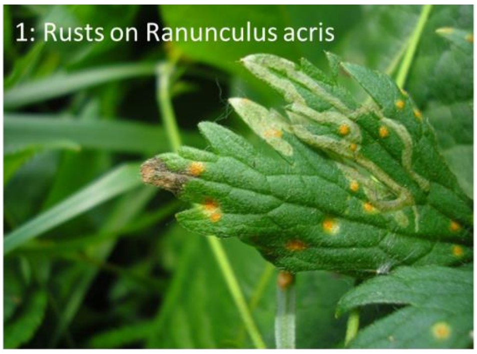
1: Rusts on Ranunculus acris

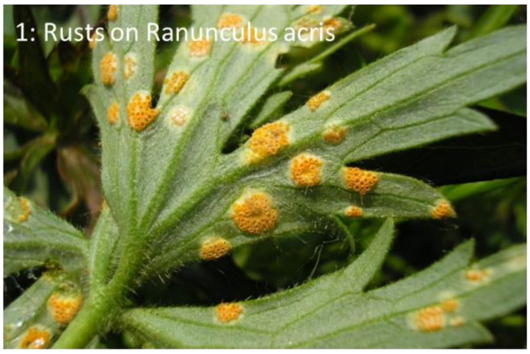
1: Rusts on Ranunculus acris

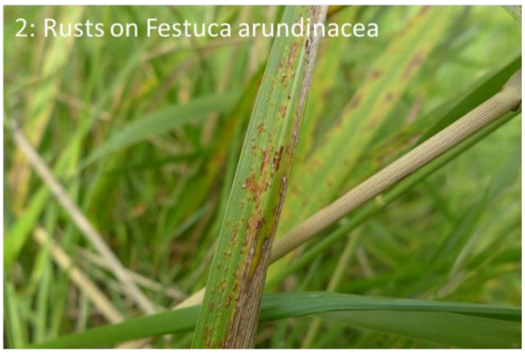
2: Rusts on Festuca arundinacea

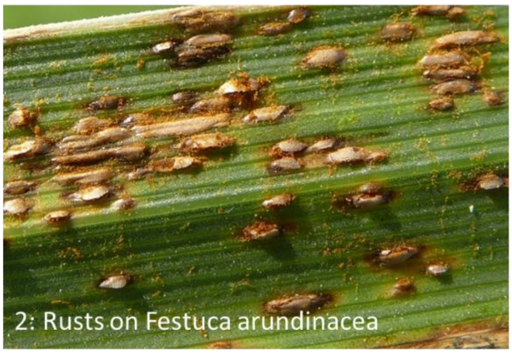
2: Rusts on Festuca arundinacea

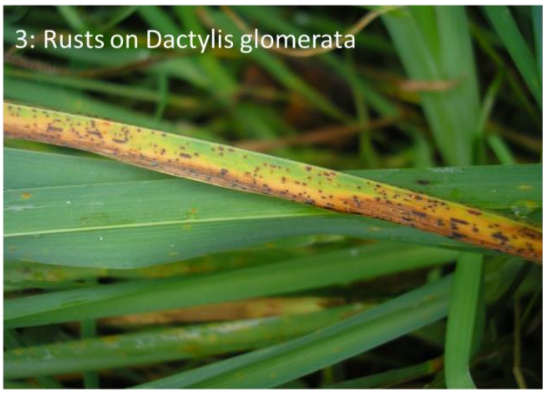
3: Rusts on Dactylis glomerata

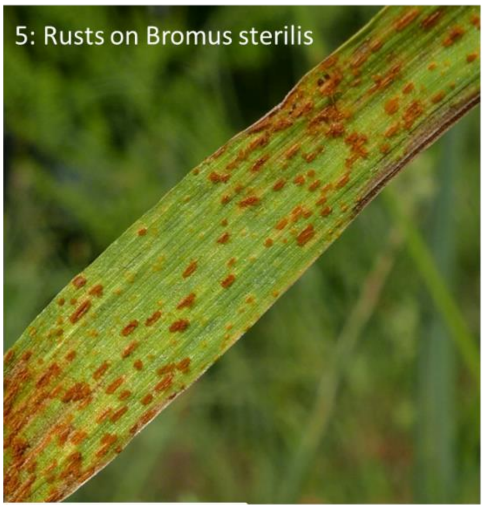
5: Rusts on Bromus sterilis

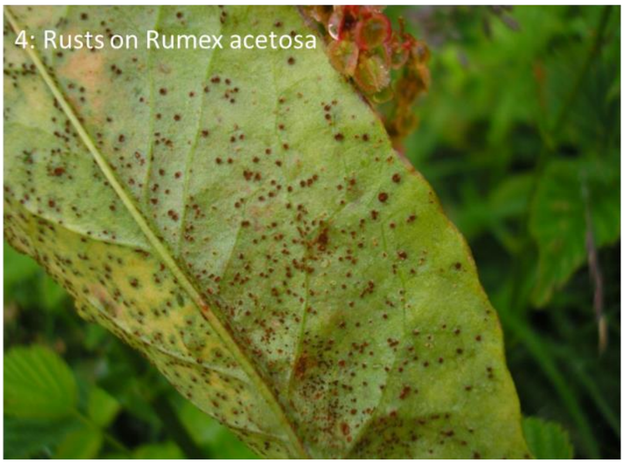
4: Rusts on Rumex acetosa

Downy mildews

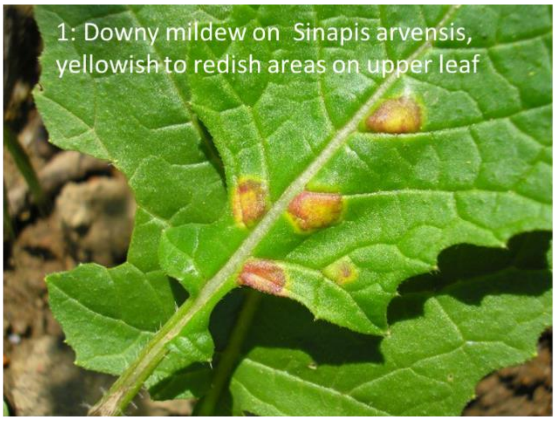
1: Downy mildew on Sinapis arvensis, yellowish to redish areas on upper leaf

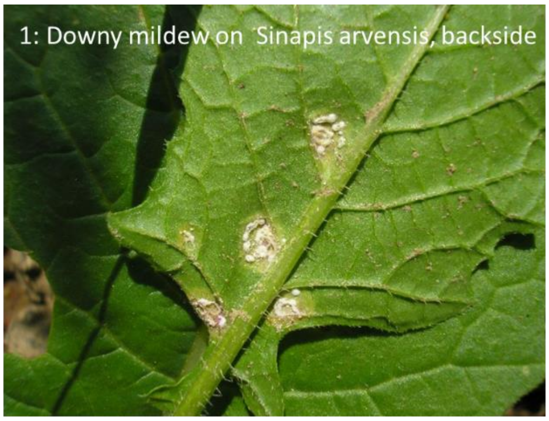
1: Downy mildew on Sinapis arvensis, backside

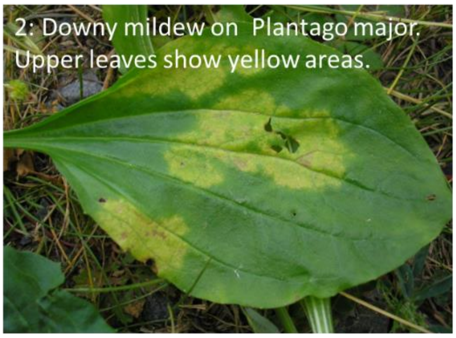
2: Downy mildew on Plantago major. Upper leaves show yellow areas.

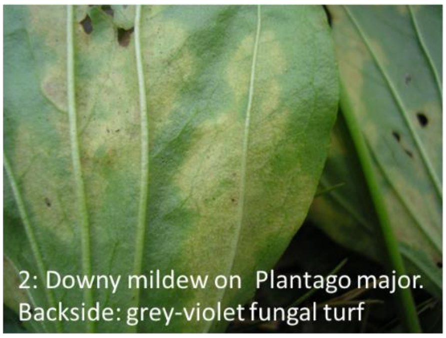
2: Downy mildew on Plantago major. Backside: grey-violet fungal turf

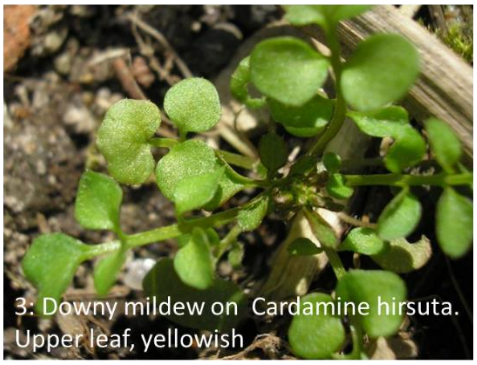
3: Downy mildew on Cardamine hirsuta. Upper leaf, yellowish

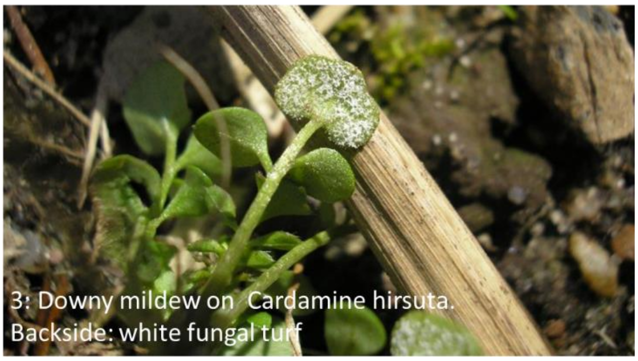
3: Downy mildew on Cardamine hirsuta. Backside: white fungal turf

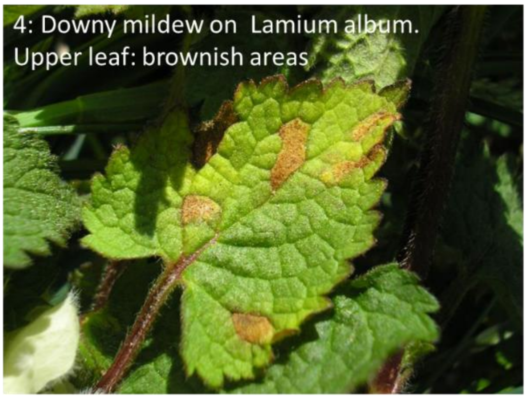
4: Downy mildew on Lamium album. Upper leaf: brownish areas

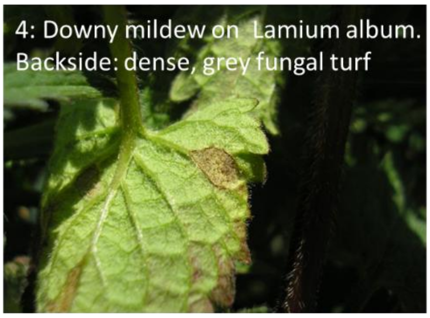
4: Downy mildew on Lamium album. Backside: dense, grey fungal turf

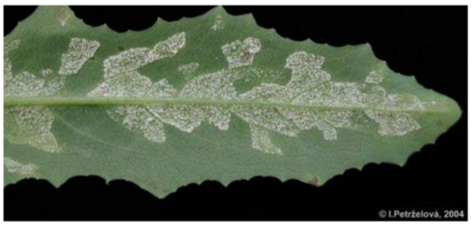

Powdery mildews

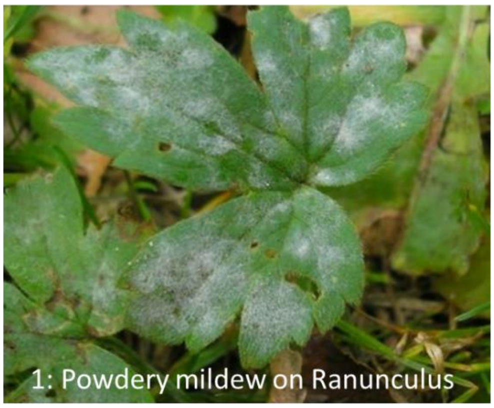
1: Powdery mildew on Ranunculus

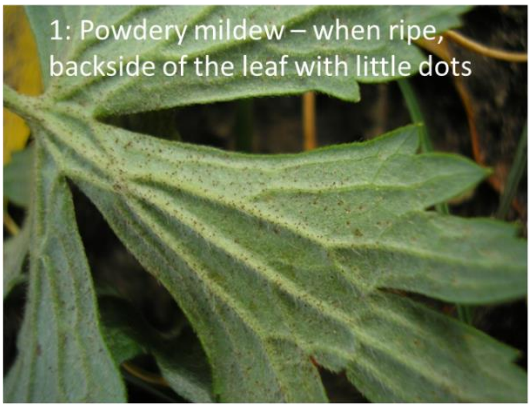
1: Powdery mildew – when ripe, backside of the leaf with little dots

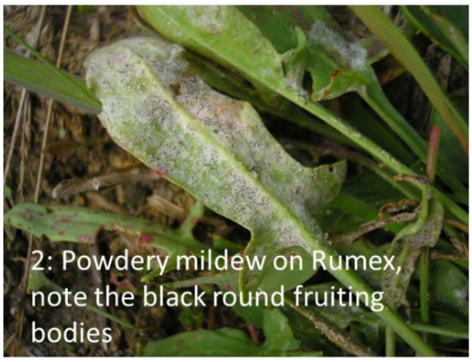
2: Powdery mildew on Rumex, note the black round fruiting bodies

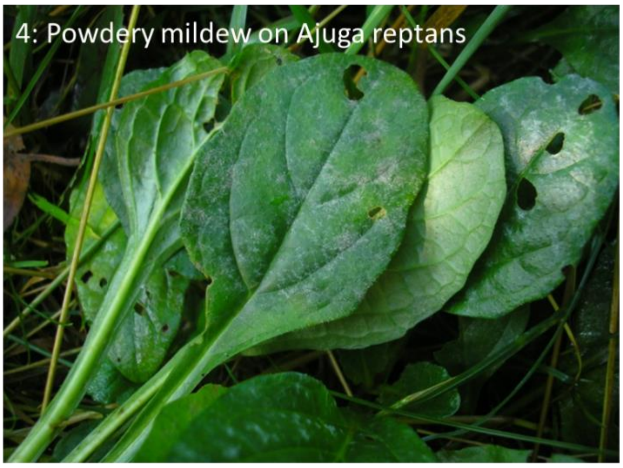
4: Powdery mildew on Ajuga reptans

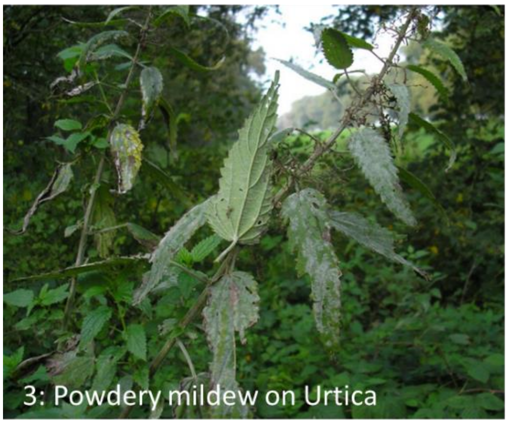
3: Powdery mildew on Urtica

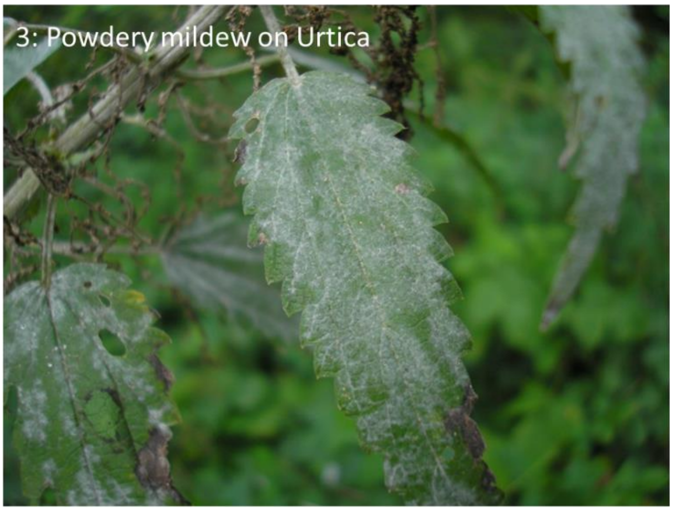
3: Powdery mildew on Urtica

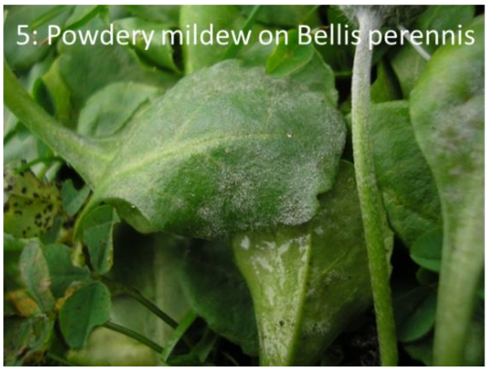
5: Powdery mildew on Bellis perennis

Leaf spots

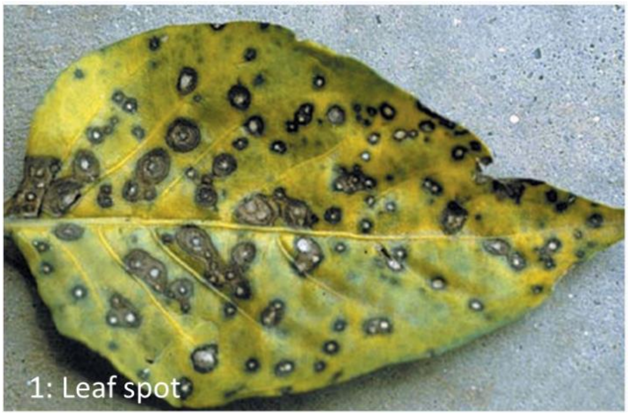

1: Leaf spot

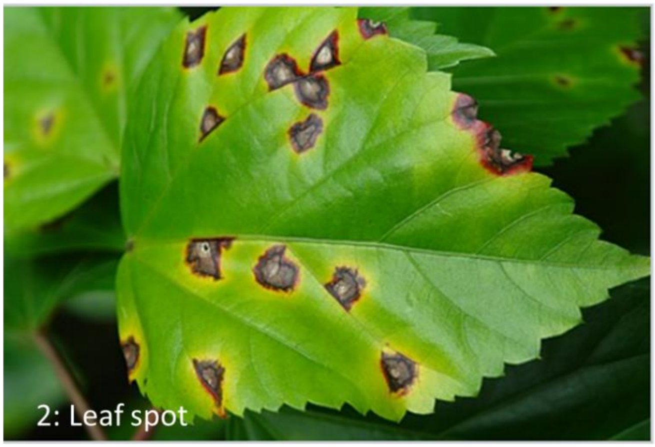

2: Leaf spot

References for damage pictures from other sources

1-4 Downy mildews: http://jule.pflanzenbestimmung.de/pflanzen-und-pilze/phytoparasitische-kleinpilze/

1-5 Powdery mildews: http://jule.pflanzenbestimmung.de/echte-mehltaupilze/

1-5 Rusts: http://jule.pflanzenbestimmung.de/rostpilze-pucciniomycotina/

1: Leaf spot: Leaf spot – Wikipedia

5: Pannel Leaf mining: from the HerbVar Network (herbvar.org)

1: Galls: Pannel on galls from the HerbVar Network (herbvar.org)

2: Galls : https://www.sunnysports.com/blog/remarkable-homemaking-skill-gall-wasp/
