# Supplementary material for: The Bug‐Network (BugNet): A Global Experimental Network Testing the Effects of Invertebrate Herbivores and Fungal Pathogens on Plant Communities and Ecosystem Function in Open Ecosystems
Source: Ecol Evol. 2025 Oct 9;15(10):e72111. doi: 10.1002/ece3.72111 (PMC12509180; doi:10.1002/ece3.72111)
Supplement: Supplementary file 3 — Appendix S3: ece372111‐sup‐0003‐AppendixS3.pdf. [file ECE3-15-e72111-s001.pdf]

Power Analysis_skript

Anne Kempel

#Load libraries

```
library(dplyr)
library(lme4)
library(broom.mixed)
library(simr)
```

Approach 1 - Power analysis

We simulate a simple dataset with the variation from the baseline biomass data per site. We simulate a dataset with 35 sites, each with 24 plots, and 8 treatment combinations which are replicated three times, according to the design of BugNet. We then simulate different effects of different magnitude, according to the methods described in Kempel et al. 2025.

Simulate a simple dataset

We use site variation, block variation and plot variation from our real dataset, from the first year of treatment application from 35 BugNet sites

- Variation between sites: 372.69
- Variation between plots within a site, based on mean and SD across blocks per site and then averaged across sites: 7.82
- Plot variation within blocks: 15.33418

```
# Set seed for reproducibility
set.seed(123)

# Define study design
n_sites <- 35              # Number of sites
n_plots <- 24              # Plots per site
n_blocks <- 3              # Blocks per site
plots_per_block <- n_plots / n_blocks  # 8 plots per block
n_treatments <- 8          # Number of treatment combinations
n_unique_blocks <- 105

# Define treatment structure (fixed order within blocks)
treatments <- expand.grid(
  insecticide = c(0, 1),
  fungicide = c(0, 1),
  molluscicide = c(0, 1)
) %>%
  arrange(insecticide, fungicide, molluscicide)  # Ensure consistent order

# Expand to match study design
data <- expand.grid(
  site_id = 1:n_sites,  # Site names A-Z etc.
  block = 1:n_blocks,                    # Blocks 1 to 3
  plot_id = 1:plots_per_block             # Temporary numbering (1-8 per block)
) %>%
  arrange(site_id, block, plot_id) %>%    # Ensure correct order
  mutate(
    plot_id = plot_id + (block - 1) * plots_per_block  # Shift plot numbers for blocks 2 &
        3
  ) %>%
  group_by(site_id, block) %>%
  mutate(
    insecticide = rep(treatments$insecticide, length.out = n()),
    fungicide = rep(treatments$fungicide, length.out = n()),
    molluscicide = rep(treatments$molluscicide, length.out = n())
  ) %>%
  ungroup() %>%
  arrange(site_id, plot_id)  # Ensure final ordering by site & plot

data <- data %>%
  arrange(site_id, block) %>%
  mutate(
    unique_block = (site_id - 1) * n_blocks + block
  )

# Simulate random effects (site, and plot variation)
data <- data %>%
  mutate(
    site_effect = rnorm(n_sites, mean = 0, sd = 372.69)[site_id],  # Site-level variance
    unique_block_effect = rnorm (n_unique_blocks, mean = 0, sd = 7.82)[unique_block],  #in
        dividual block variance
    plot_effect = rnorm(nrow(.), mean = 0, sd = 15.33)  # Individual plot variance
  )

print(data)
```

```
# Convert some variables to factors
data$site_id<- as.factor(data$site_id)
data$plot_id<-as.factor(data$plot_id)
data$unique_block<-as.factor(data$unique_block)
head(data)
```

Model different scenarios

No interaction

This is the code to model a main effect of 5 %, but no interaction. The power analysis tells me the power to detect such an effect.

```
# Define fixed effects (assumed effect sizes)
beta0 <- 100  # Baseline biomass
beta_insecticide <- 5 # Effect of insect exclusion
beta_fungicide <- 5  # Effect of fungi exclusion
beta_molluscicide <- 5  # Effect of mollusc exclusion

# Interaction effects
beta_fung_moll_int <- 0  # No interaction between fungicide and molluscicide
beta_insect_moll_int <- 0  # No effect (set to zero)
beta_insect_fung_int<- 0   # No fungicide insecticide interaction
beta_three_way_int <- 0  # No three-way interaction

# Simulate biomass response
data_sim <- data %>%
  mutate(
    biomass = beta0 +
      beta_insecticide * insecticide +
      beta_fungicide * fungicide +
      beta_molluscicide * molluscicide +
      beta_fung_moll_int * fungicide * molluscicide +  # No effect
      beta_insect_moll_int * insecticide * molluscicide +  # No effect
      beta_insect_fung_int * insecticide * fungicide + # No effect
      beta_three_way_int * insecticide * fungicide * molluscicide +  # No effect
      site_effect + unique_block_effect + plot_effect )

# Fit the mixed model
M_sim <- lmer(biomass ~ insecticide * fungicide * molluscicide +
              (1 | site_id) + (1|unique_block) , data = data_sim)

summary(M_sim)
car::Anova(M_sim)
```

Run Power Analysis from the simr package

```
# Power for the insecticide effect
powerSim(M_sim, fixed("insecticide"), nsim = 300)

#Record results in table
```

Compensatory effects

This is the code to simulate a compensatory effect. E.g. a main effect of 5 % but the effects cancel each other out in an interaction. E.g. a main effect of +5 will then have a -10 in the interaction.

```
# Define fixed effects (assumed effect sizes)
beta0 <- 100  # Baseline biomass
beta_insecticide <- 5 # Effect of insect exclusion
beta_fungicide <- 5  # Effect of fungi exclusion
beta_molluscicide <- 5  # Effect of mollusc exclusion

# Interaction effects
beta_fung_moll_int <- -10  # Compensatory effect, cancel main effect out
beta_insect_moll_int <- 0  # No effect (set to zero)
beta_insect_fung_int<- 0   # No fungicide insecticide interaction
beta_three_way_int <- 0  # No three-way interaction

# Simulate biomass response
data_sim <- data %>%
  mutate(
    biomass = beta0 +
      beta_insecticide * insecticide +
      beta_fungicide * fungicide +
      beta_molluscicide * molluscicide +
      beta_fung_moll_int * fungicide * molluscicide +  # compensatory effect
      beta_insect_moll_int * insecticide * molluscicide +  # No effect
      beta_insect_fung_int * insecticide * fungicide + # No effect
      beta_three_way_int * insecticide * fungicide * molluscicide +  # No effect
      site_effect + unique_block_effect + plot_effect)

# Fit the mixed model
M_sim <- lmer(biomass ~ insecticide * fungicide * molluscicide +
              (1 | site_id) + (1|unique_block), data = data_sim)

summary(M_sim)
car::Anova(M_sim)
```

Run Power Analysis - compensatory effects

```
# Power for the insecticide effect
powerSim(M_sim, fixed("insecticide"), nsim = 300)
powerSim(M_sim, fixed("fungicide:molluscicide"), nsim =300)
```

Additive effects

This is the code to model an additive effect, e,g, a main effects 5 but effects of interaction are even stronger than what one would expect based on their individual effect

```
# Define fixed effects (assumed effect sizes)
beta0 <- 100  # Baseline biomass
beta_insecticide <- 5 # Effect of insect exclusion
beta_fungicide <- 5  # Effect of fungi exclusion
beta_molluscicide <- 5  # Effect of mollusc exclusion

# Interaction effects
beta_fung_moll_int <- 5  # additive effect, 5 more when both together
beta_insect_moll_int <- 0  # No effect (set to zero)
beta_insect_fung_int<- 0   # No fungicide insecticide interaction
beta_three_way_int <- 0  # No three-way interaction

# Simulate biomass response
data_sim <- data %>%
  mutate(
    biomass = beta0 +
      beta_insecticide * insecticide +
      beta_fungicide * fungicide +
      beta_molluscicide * molluscicide +
      beta_fung_moll_int * fungicide * molluscicide +  #additive
      beta_insect_moll_int * insecticide * molluscicide +  # No effect
      beta_insect_fung_int * insecticide * fungicide + # No effect
      beta_three_way_int * insecticide * fungicide * molluscicide +  # No effect
      site_effect + plot_effect +
      rnorm(n(), mean = 0, sd = 5)  # Random error
  )

# Fit the mixed model
M_sim <- lmer(biomass ~ insecticide * fungicide * molluscicide +
              (1 | site_id) + (1|unique_block), data = data_sim)

summary(M_sim)
```

Run Power Analysis - compensatory effects

```
# Power for the insecticide effect
powerSim(M_sim, fixed("insecticide"), nsim = 300)
powerSim(M_sim, fixed("fungicide:molluscicide"), nsim =300)
```

Create a function

To use different effect sizes it is easier to create function. We do this with the following code.

```
#Create the function
run_simulation <- function(data,
                           beta0 = 100,
                           beta_insecticide = 0,
                           beta_fungicide = 0,
                           beta_molluscicide = 0,
                           beta_insect_fung = 0,
                           beta_insect_moll = 0,
                           beta_fung_moll = 0,
                           beta_three_way = 0,
                           test_term = "insecticide",  # Default test term (can be overrid
         den)
                           nsim = 300) {

  # Simulate biomass values with absolute effects
  data_sim <- data %>%
    mutate(
      biomass = beta0 +
        beta_insecticide * insecticide +
        beta_fungicide * fungicide +
        beta_molluscicide * molluscicide +
        beta_fung_moll * fungicide * molluscicide +
        beta_insect_moll * insecticide * molluscicide +
        beta_insect_fung * insecticide * fungicide +
        beta_three_way * insecticide * fungicide * molluscicide +
        site_effect + unique_block_effect + plot_effect
    )

  # Fit the mixed model
  model <- lmer(
    biomass ~ insecticide * fungicide * molluscicide +
      (1 | site_id) + (1 | unique_block),
    data = data_sim
  )

  # Run power analysis
  power_result <- powerSim(model, fixed(test_term), nsim = nsim)

  return(list(test = test_term, result = power_result))
}
```

No interaction

```
# Now we can run the function for the different effect sizes
# 1) no interaction
simulation_5 <- run_simulation(
  data = data,
  beta_insecticide = 5,  # 5% effect for insecticide
  beta_fungicide = 5,    # 5% effect for fungicide
  beta_molluscicide = 5, # 5% effect for molluscicide
  test_term = "insecticide"
)

simulation_7_5 <- run_simulation(
  data = data,
  beta_insecticide = 7.5,  # 7.5% effect for insecticide
  beta_fungicide = 7.5,    # 7.5% effect for fungicide
  beta_molluscicide = 7.5, # 7.5% effect for molluscicide
  test_term = "insecticide"
)

simulation_10 <- run_simulation(
  data = data,
  beta_insecticide = 10,  # 10% effect for insecticide
  beta_fungicide = 10,    # 10% effect for fungicide
  beta_molluscicide = 10, # 10% effect for molluscicide
  test_term = "insecticide"
)

simulation_15 <- run_simulation(
  data = data,
  beta_insecticide = 15,  # 15% effect for insecticide
  beta_fungicide = 15,    # 15% effect for fungicide
  beta_molluscicide = 15, # 15% effect for molluscicide
  test_term = "insecticide"
)

simulation_20 <- run_simulation(
  data = data,
  beta_insecticide = 20,  # 20% effect for insecticide
  beta_fungicide = 20,    # 20% effect for fungicide
  beta_molluscicide = 20, # 20% effect for molluscicide
  test_term = "insecticide"
)

simulation_5
simulation_7_5
simulation_10
simulation_15
simulation_20
```

Compensatory effects

```
# 2) Compensatory effects
comp_simulation_5 <- run_simulation(
  data = data,
  beta_insecticide = 5,  # 5% effect for insecticide
  beta_fungicide = 5,    # 5% effect for fungicide
  beta_molluscicide = 5, # 5% effect for molluscicide
  beta_fung_moll = -10,
  test_term = "fungicide:molluscicide"
)

comp_simulation_7_5 <- run_simulation(
  data = data,
  beta_insecticide = 7.5,  # 7.5% effect for insecticide
  beta_fungicide = 7.5,    # 7.5% effect for fungicide
  beta_molluscicide = 7.5, # 7.5% effect for molluscicide
  beta_fung_moll = -15,
  test_term = "fungicide:molluscicide"
)

comp_simulation_10 <- run_simulation(
  data = data,
  beta_insecticide = 10,  # 10% effect for insecticide
  beta_fungicide = 10,    # 10% effect for fungicide
  beta_molluscicide = 10, # 10% effect for molluscicide
  beta_fung_moll = -20,
  test_term = "fungicide:molluscicide"
)

comp_simulation_15 <- run_simulation(
  data = data,
  beta_insecticide = 15,  # 15% effect for insecticide
  beta_fungicide = 15,    # 15% effect for fungicide
  beta_molluscicide = 15, # 15% effect for molluscicide
  beta_fung_moll = -30,
  test_term = "fungicide:molluscicide"
)

comp_simulation_20 <- run_simulation(
  data = data,
  beta_insecticide = 20,  # 20% effect for insecticide
  beta_fungicide = 20,    # 20% effect for fungicide
  beta_molluscicide = 20, # 20% effect for molluscicide
  beta_fung_moll = -40,
  test_term = "fungicide:molluscicide"
)

comp_simulation_5
comp_simulation_7_5
comp_simulation_10
comp_simulation_15
comp_simulation_20
```

Superadditive effects

```
# 3) Superadditive effects
add_simulation_5 <- run_simulation(
  data = data,
  beta_insecticide = 5,  # 5% effect for insecticide
  beta_fungicide = 5,    # 5% effect for fungicide
  beta_molluscicide = 5, # 5% effect for molluscicide
  beta_fung_moll = 5,
  test_term = "fungicide:molluscicide"
)

add_simulation_7_5 <- run_simulation(
  data = data,
  beta_insecticide = 7.5,  # 7.5% effect for insecticide
  beta_fungicide = 7.5,    # 7.5% effect for fungicide
  beta_molluscicide = 7.5, # 7.5% effect for molluscicide
  beta_fung_moll = 7.5,
  test_term = "fungicide:molluscicide"
)

add_simulation_10 <- run_simulation(
  data = data,
  beta_insecticide = 10,  # 10% effect for insecticide
  beta_fungicide = 10,    # 10% effect for fungicide
  beta_molluscicide = 10, # 10% effect for molluscicide
  beta_fung_moll = 10,
  test_term = "fungicide:molluscicide"
)

add_simulation_15 <- run_simulation(
  data = data,
  beta_insecticide = 15,  # 15% effect for insecticide
  beta_fungicide = 15,    # 15% effect for fungicide
  beta_molluscicide = 15, # 15% effect for molluscicide
  beta_fung_moll = 15,
  test_term = "fungicide:molluscicide"
)

add_simulation_20 <- run_simulation(
  data = data,
  beta_insecticide = 20,  # 20% effect for insecticide
  beta_fungicide = 20,    # 20% effect for fungicide
  beta_molluscicide = 20, # 20% effect for molluscicide
  beta_fung_moll = 20,
  test_term = "fungicide:molluscicide"
)

add_simulation_5
add_simulation_7_5
add_simulation_10
add_simulation_15
add_simulation_20
```
