# Supplementary material for: The Bug‐Network (BugNet): A Global Experimental Network Testing the Effects of Invertebrate Herbivores and Fungal Pathogens on Plant Communities and Ecosystem Function in Open Ecosystems
Source: Ecol Evol. 2025 Oct 9;15(10):e72111. doi: 10.1002/ece3.72111 (PMC12509180; doi:10.1002/ece3.72111)
Supplement: Supplementary file 4 — Appendix S4: ece372111‐sup‐0004‐AppendixS4.docx. [file ECE3-15-e72111-s003.docx]

**Supporting Material**

**Content:**

- **Table S1: Information on site characteristics, including vegetation type and land management**
- **Figure S1: Schematic figure of the Bug-Network**
- **SM 1: Experimental protocol provided to collaborators (pdf)**
- **SM 2: Detailed protocol on damage assessment and the selection of individuals that was provided to collaborators (pdf)**
- **SM 3: Code to produce the Power Analysis**
- **SM 4: Picture Gallery of some BugNet Sites**

**Table S1: Information on site characteristics of all existing BugNet experimental sites, including vegetation type and land management**

| **Site Name** | **Country** | **Continent** | **Latidude** | **Longitude** | **Vegetation type** | **Land management** |
| --- | --- | --- | --- | --- | --- | --- |
| Changling | China | Asia | 44.59 | 123.51 | grassland | mown once a year |
| Lanzhou | China | Asia | 33.67333 | 101.864167 | alpine meadow | moderate grazing in winter by yaks |
| Tian | China | Asia | 37.2 | 102.79 | alpine meadow | grazing excluded |
| Zoige | China | Asia | 33.67071677 | 103.0653608 | alpine grassland | light grazing with ~1.89 yak per hectare |
| Challakere | India | Asia | 14.42853945 | 76.58137002 | tropical savannah | occasionally mown |
| Kashmir | India | Asia | 34.049953 | 74.398083 | grassland with forest community | mown once a year |
| Hezar-Masjed | Iran | Asia | 36.73 | 59.46 | complex of shrubs and herbaceous plants | unmanaged |
| UNSW Fowlers | Australia | Australia | -31.072763 | 141.765116 | arid shrubland on plains | ungrazed by sheep since 2023; moderate grazing by goats and kangaroos |
| Western Sydney | Australia | Australia | -33.720404 | 150.753276 | subhumid grassy woodland | conservation reserve grazed by low levels of kangaroos |
| Pruhonice | Czech Republic | Europe | 49.990668 | 14.568589 | grassland | mown once a year |
| Raja | Estonia | Europe | 58.362629 | 26.679525 | grassland | mown once a year |
| Sillukse | Estonia | Europe | 58.622019 | 23.54637 | grassland | mown once a year |
| Grande Ferrade | France | Europe | 44.791028 | -0.575867 | temperate grassland | mown once a year |
| BadBug | Germany | Europe | 51.39151 | 11.878728 | grassland | mown twice a year |
| Bonn | Germany | Europe | 50.61353 | 6.995998 | grassland | mown three times per year |
| Silwood | Great Britain | Europe | 51.41441 | -0.65226 | grassland | mown once a year |
| FRI | Greece | Europe | 40.50955296 | 23.08134522 | dry grassland | unmanaged |
| Patras | Greece | Europe | 38.298178 | 21.800925 | grassland | mown once a year |
| Thessaloniki | Greece | Europe | 40.505387 | 22.958145 | abandoned grassland | unmanaged |
| Oppdal high | Norway | Europe | 62.30759 | 9.67548 | alpine tundra | light grazing, 25-50 sheep per km2; 0.8 wild reindeer per km2 |
| Oppdal low | Norway | Europe | 62.29746 | 9.62976 | alpine tundra | light grazing, 25-50 sheep per km2; 0.8 wild reindeer per km2 |
| Oppdal middle | Norway | Europe | 62.30553 | 9.65095 | alpine tundra | light grazing, 25-50 sheep per km2; 0.8 wild reindeer per km2 |
| Villarroya | Spain | Europe | 41.15811888 | -1.331593382 | mediterranean shrubland / grassland | unmanaged, light herbivory by wild rabbits |
| Bern | Switzerland | Europe | 47.025224 | 7.459026 | grassland | mown twice a year |
| Davos high | Switzerland | Europe | 46.76053 | 9.8578 | alpine tundra | unmanaged |
| Davos low | Switzerland | Europe | 46.77506 | 9.81216 | mountain meadow | mown twice a year |
| Davos middle | Switzerland | Europe | 46.76994 | 9.83265 | alpine meadow | mown once a year |
| Quebec | Canada | North America | 45.89251 | -74.404149 | oldfield | mown every 3-4 years |
| AUM | USA | North America | 32.365 | -86.186972 | temperate grassland | mown once a year |
| McP | USA | North America | 36.09583 | -97.19917 | grassland | fire every 5-8 years |
| UMBS | USA | North America | 45.5587 | -84.6775 | temperate grassland | mown every few years |
| Berraondo | Argentina | South America | -38.416552 | -62.823034 | temperate grassland | light grazing, 25-75 livestock unit per km2 |
| Naposta | Argentina | South America | -38.4239389 | -62.28791667 | temperate grassland | unmanaged |
| Naredo | Argentina | South America | -36.841139 | -62.450944 | temperate grassland | grazing excluded, mown once a year |
| Coyhaique | Chile | South America | -45.559381 | -71.974604 | temperate grassland | occacionally mown |
| Alamala | Ecuador | South America | -3.999137 | -79.446909 | dry inter-andean shrubland | unmanaged |

####
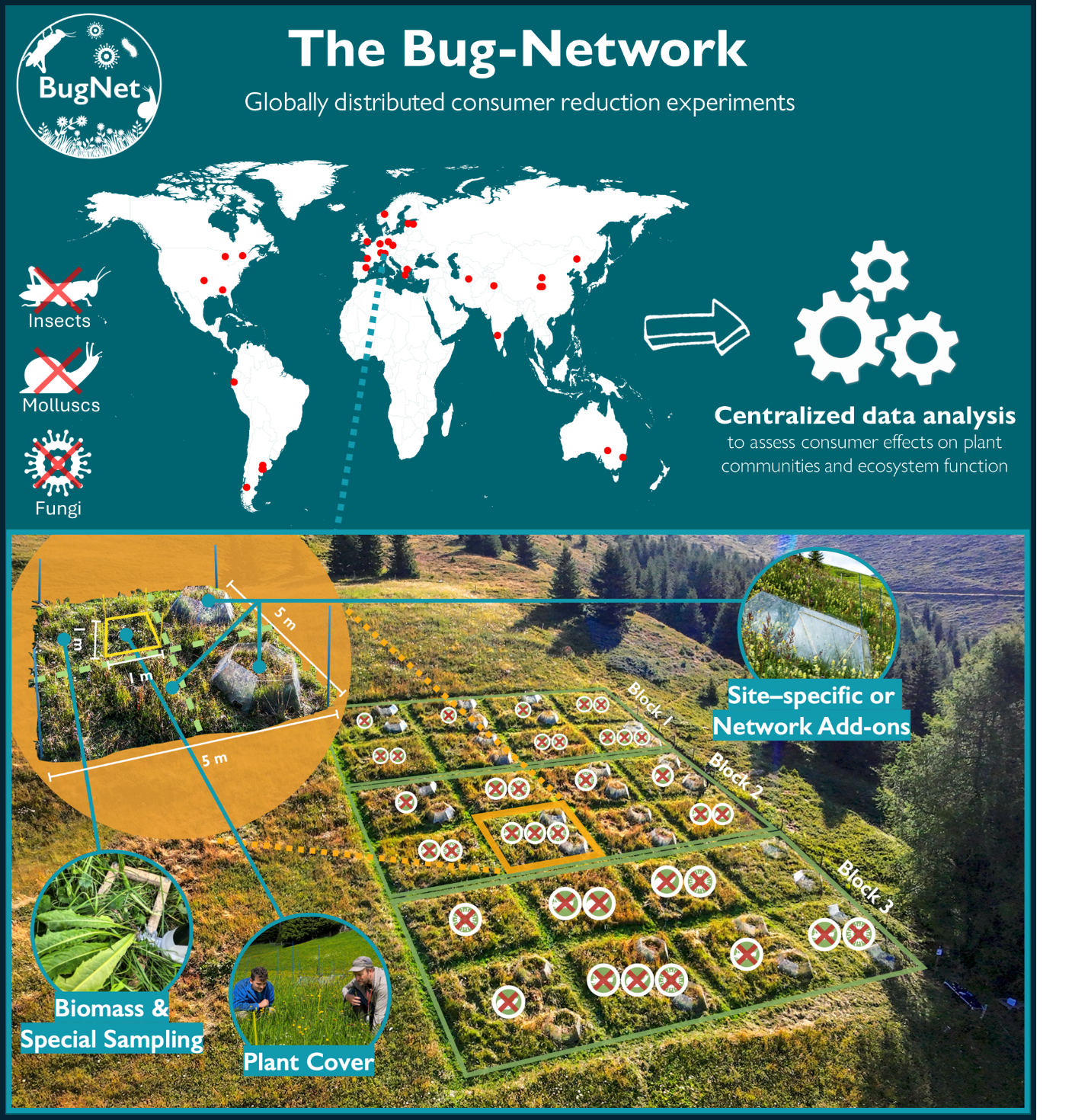


**Figure S1.** *Schematic overview of the Bug-Network.* Across multiple sites worldwide, collaborators conduct consumer exclusion experiments in which insects, molluscs, and fungal pathogens are removed individually and in combination using biocides, following a standardized experimental design. Core measurements—such as plant cover and biomass—are collected, along with optional site-specific or network-wide add-ons. All data are analyzed centrally to quantify the general impacts of consumers on plant communities and ecosystem functioning, evaluate interactive effects, and assess the context dependency of plant–consumer interactions. Depicted is an image of a BugNet site in the Swiss Alps, which participates in the network Warming Add-on.

#### Supporting Material 4: Picture Gallery of BugNet sites


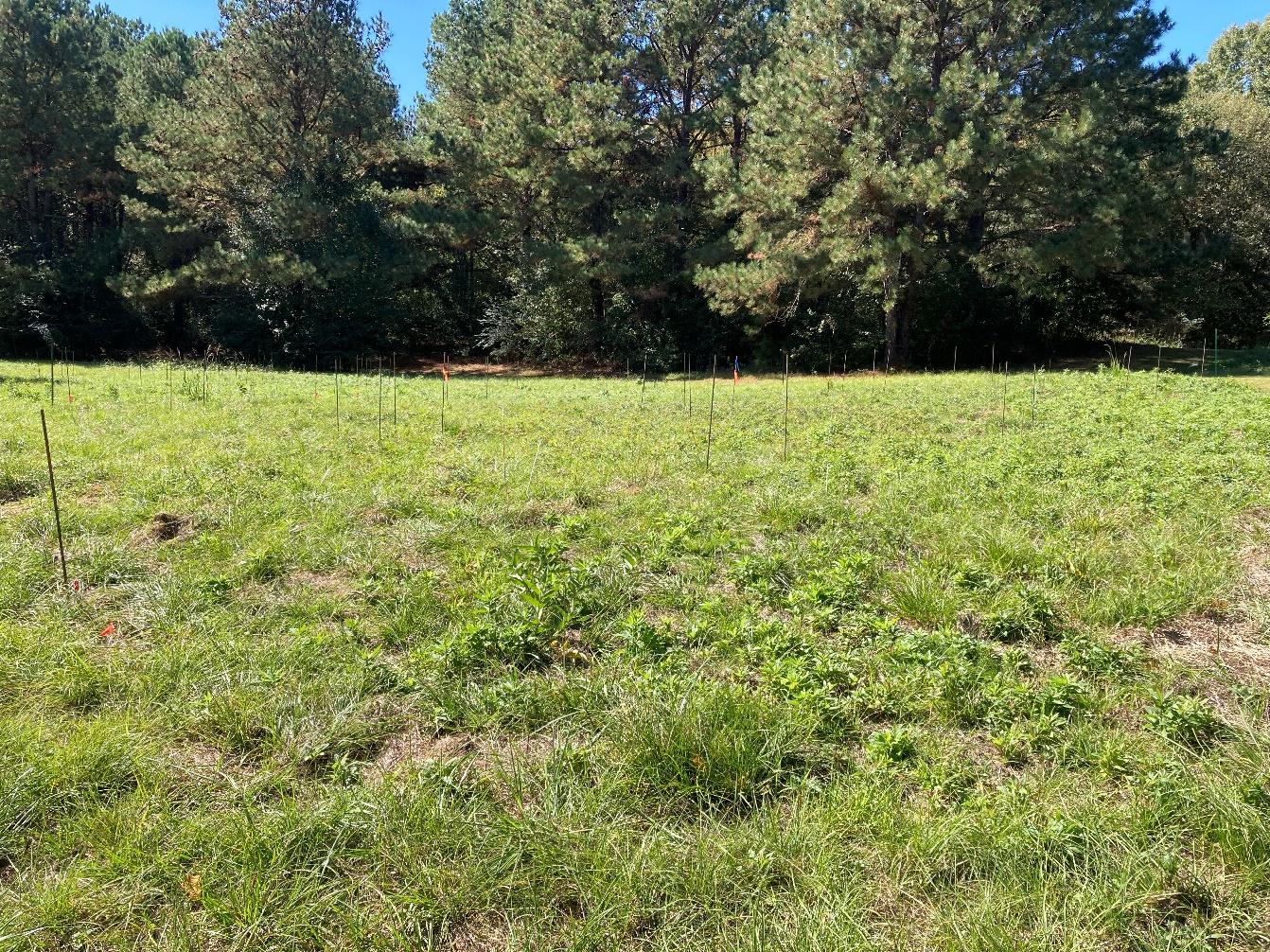


Site Name: USA-AUM


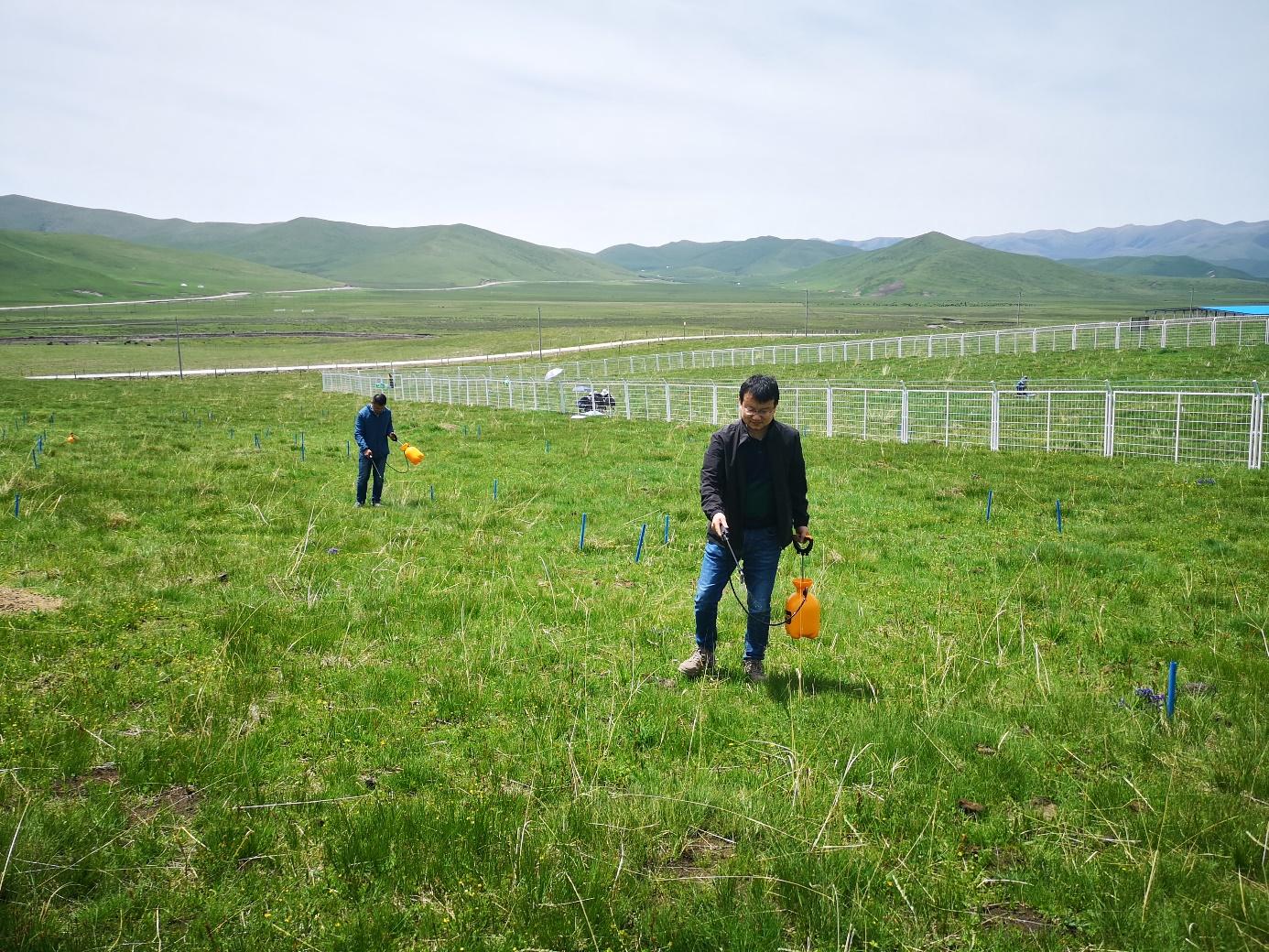


Site Name: CHN - Lanzhou


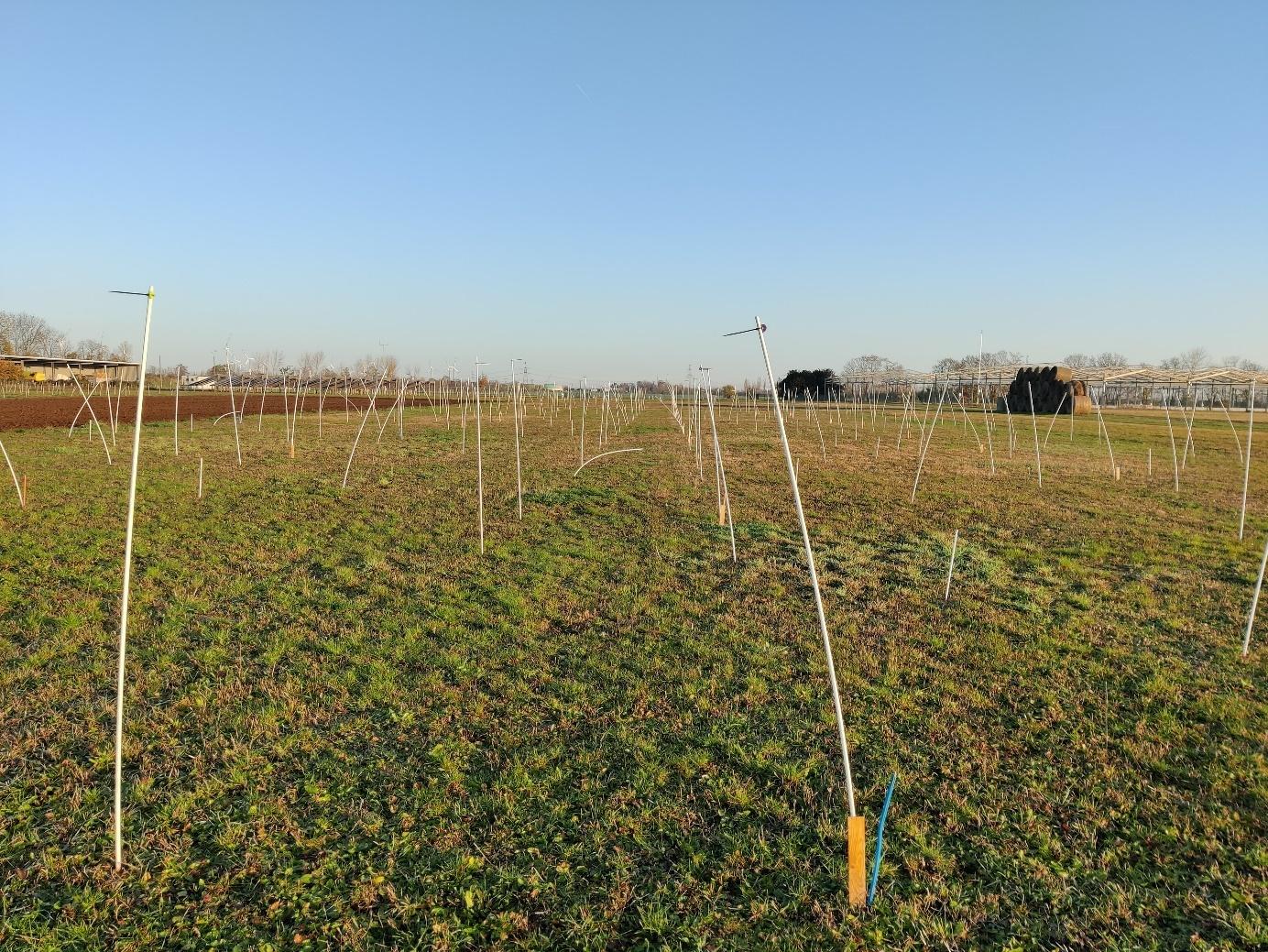


Site Name: DEU - BadBug


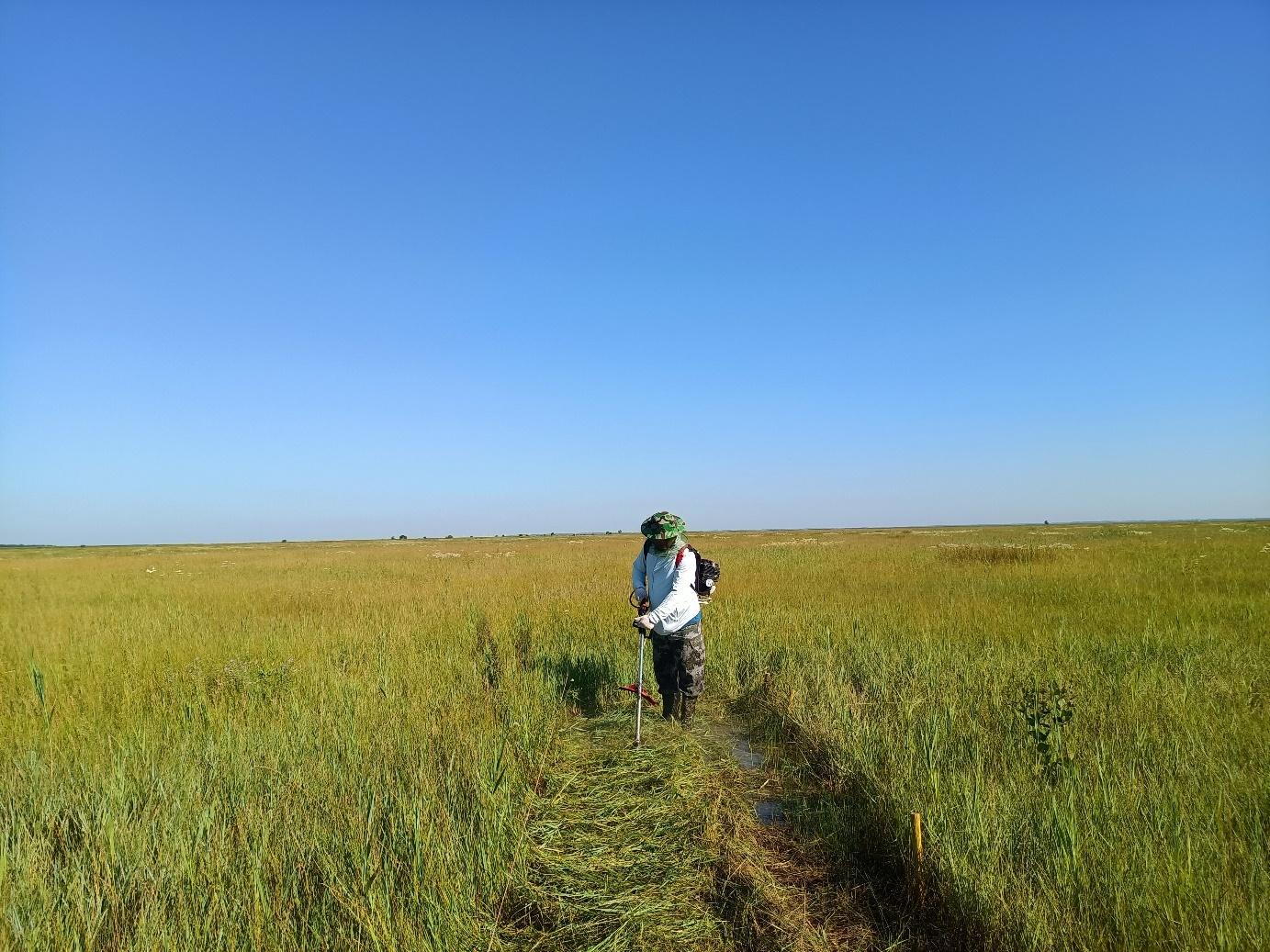


Site Name: CHN - Changling


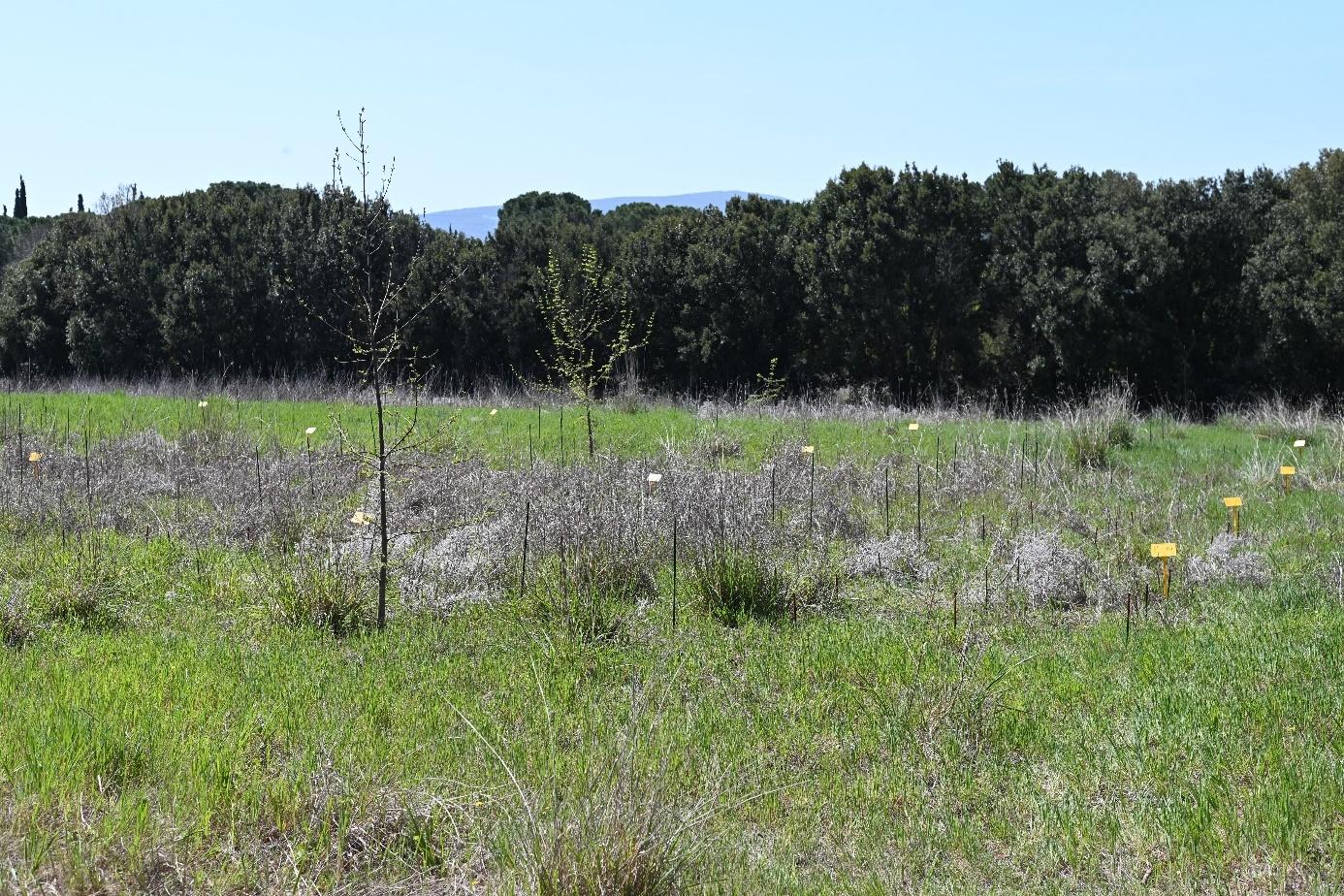


Site Name: GRC - FRI


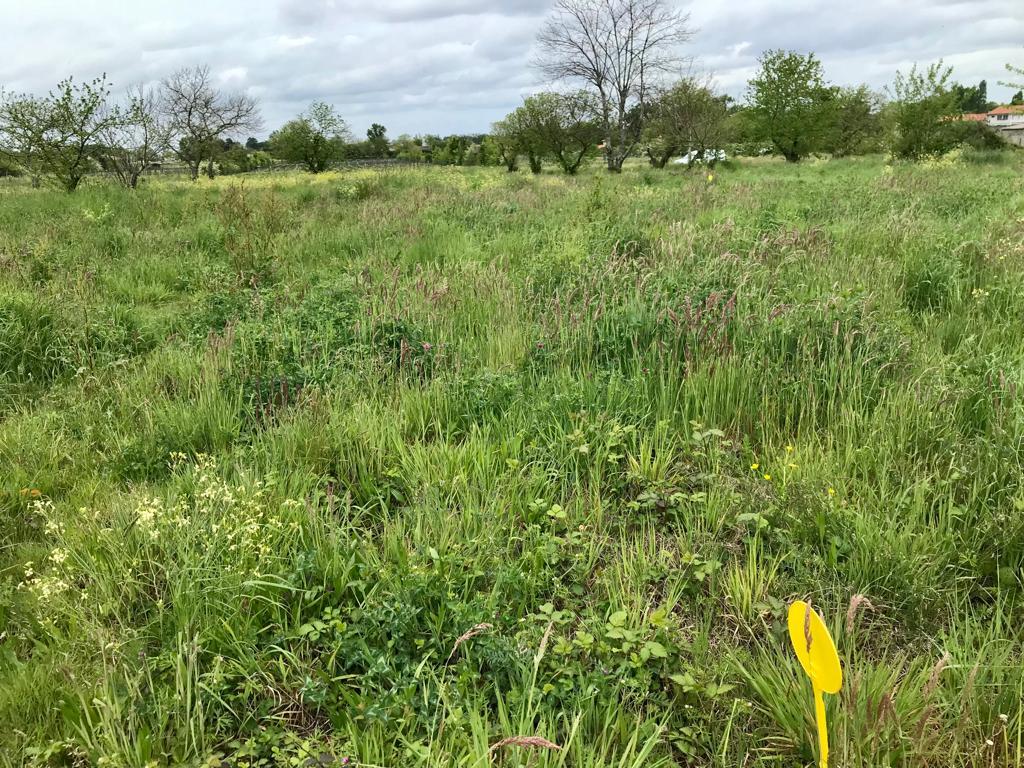


Site Name: FRA - Grande Ferrade


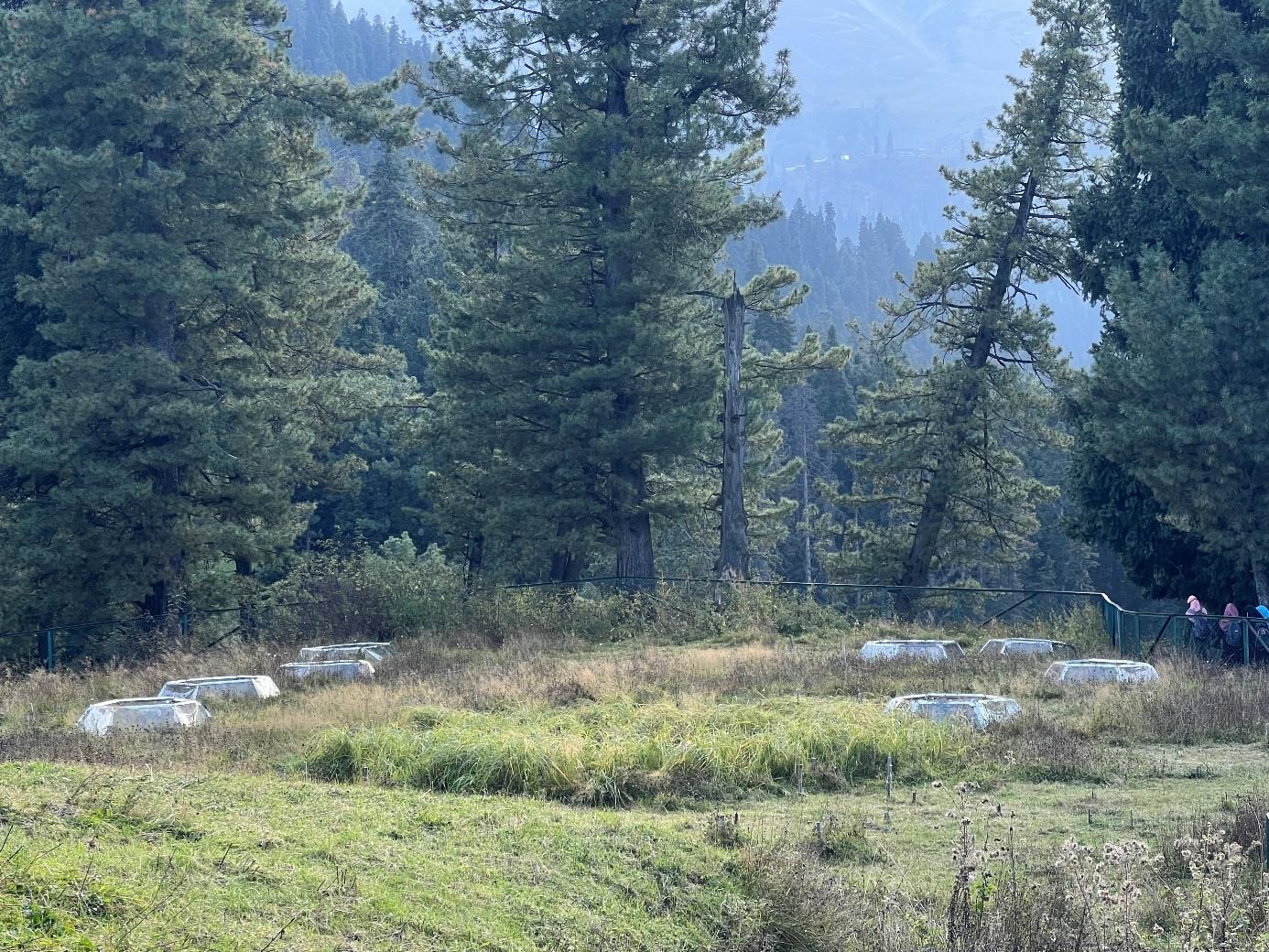


Site Name: IND - Kashmir


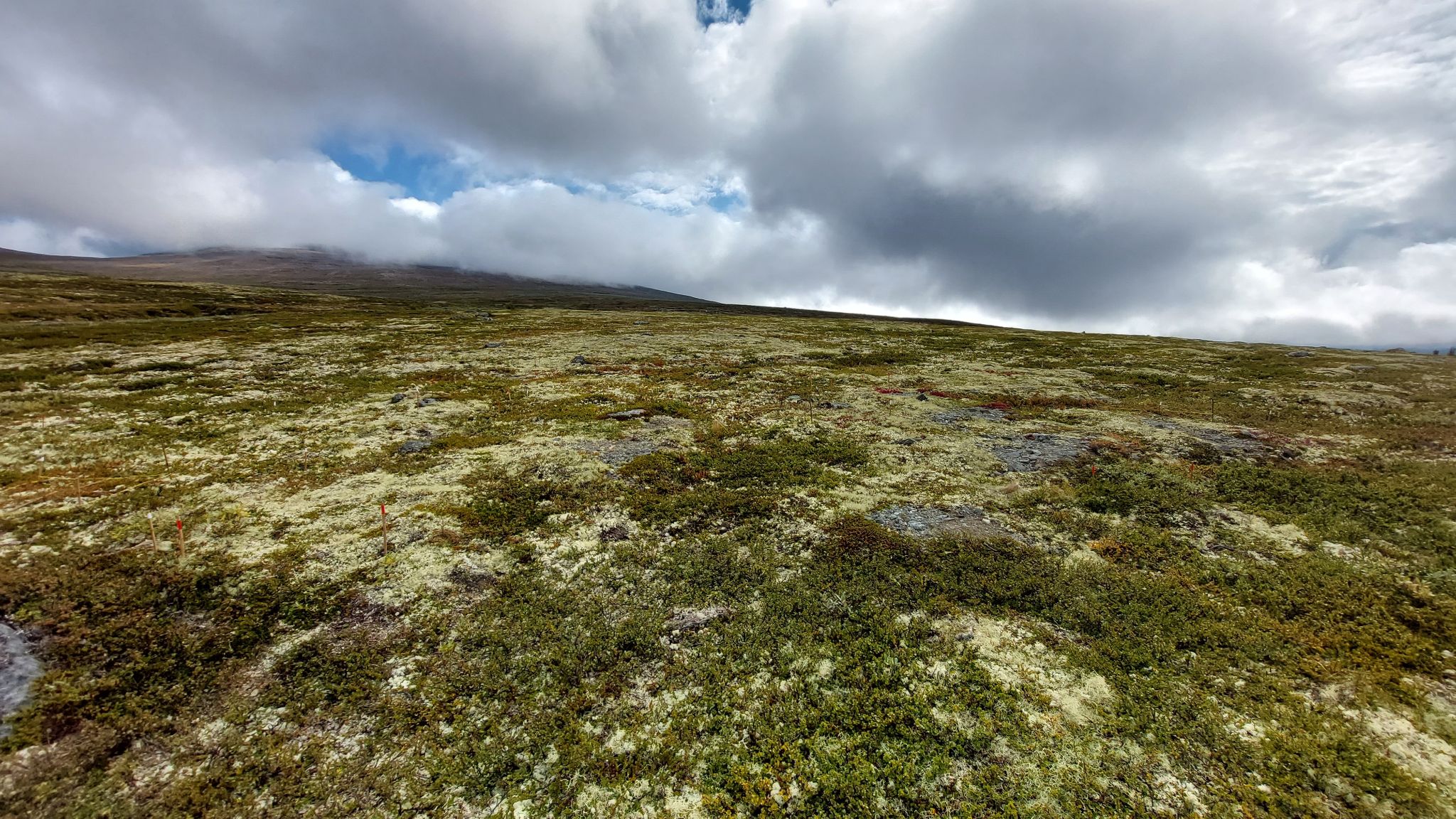


Site Name: NOR – Oppdal low


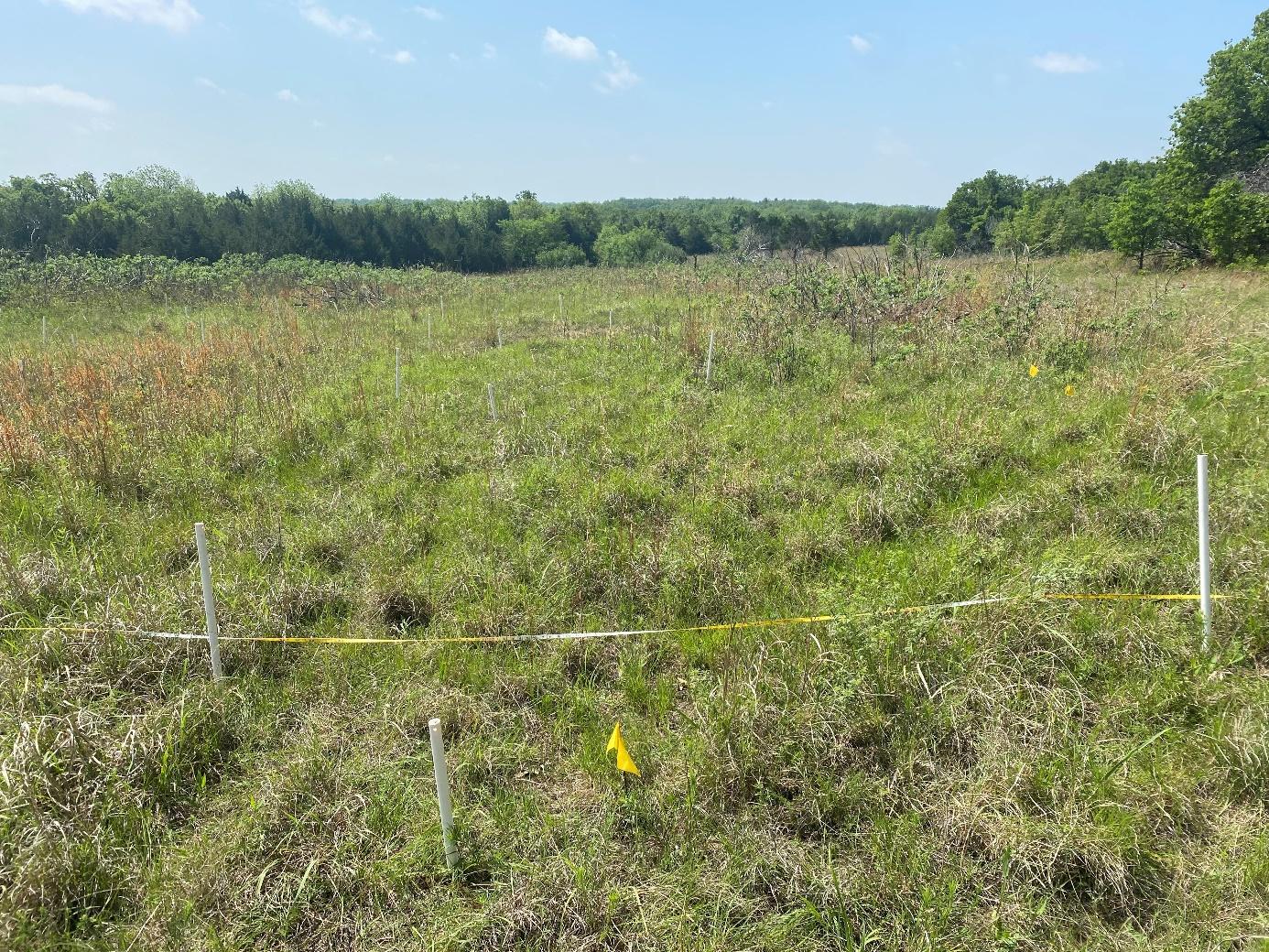


Site Name: USA - McP


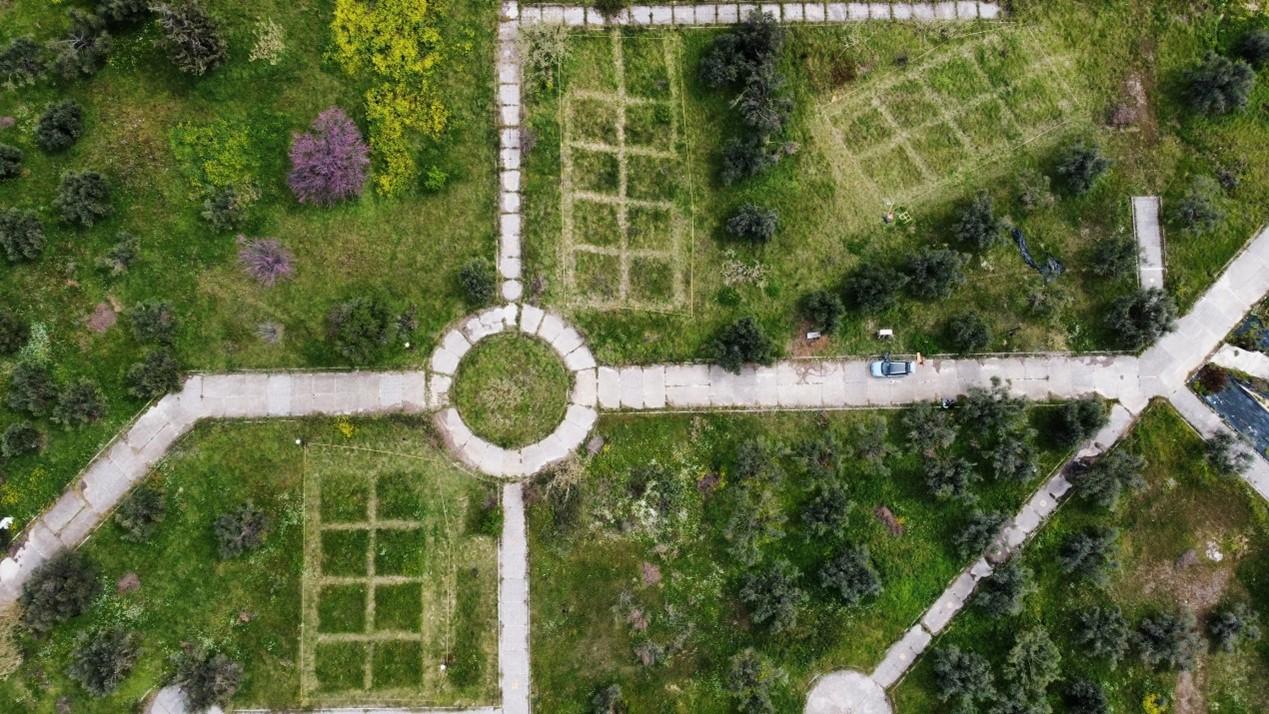


Site Name: GRC - Patras


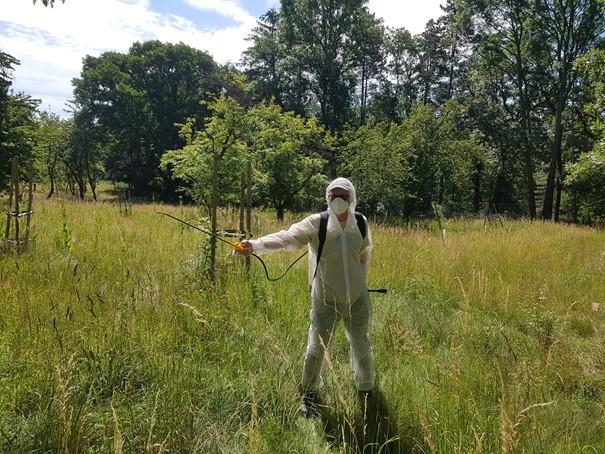


Site Name: CZE - Pruhonice


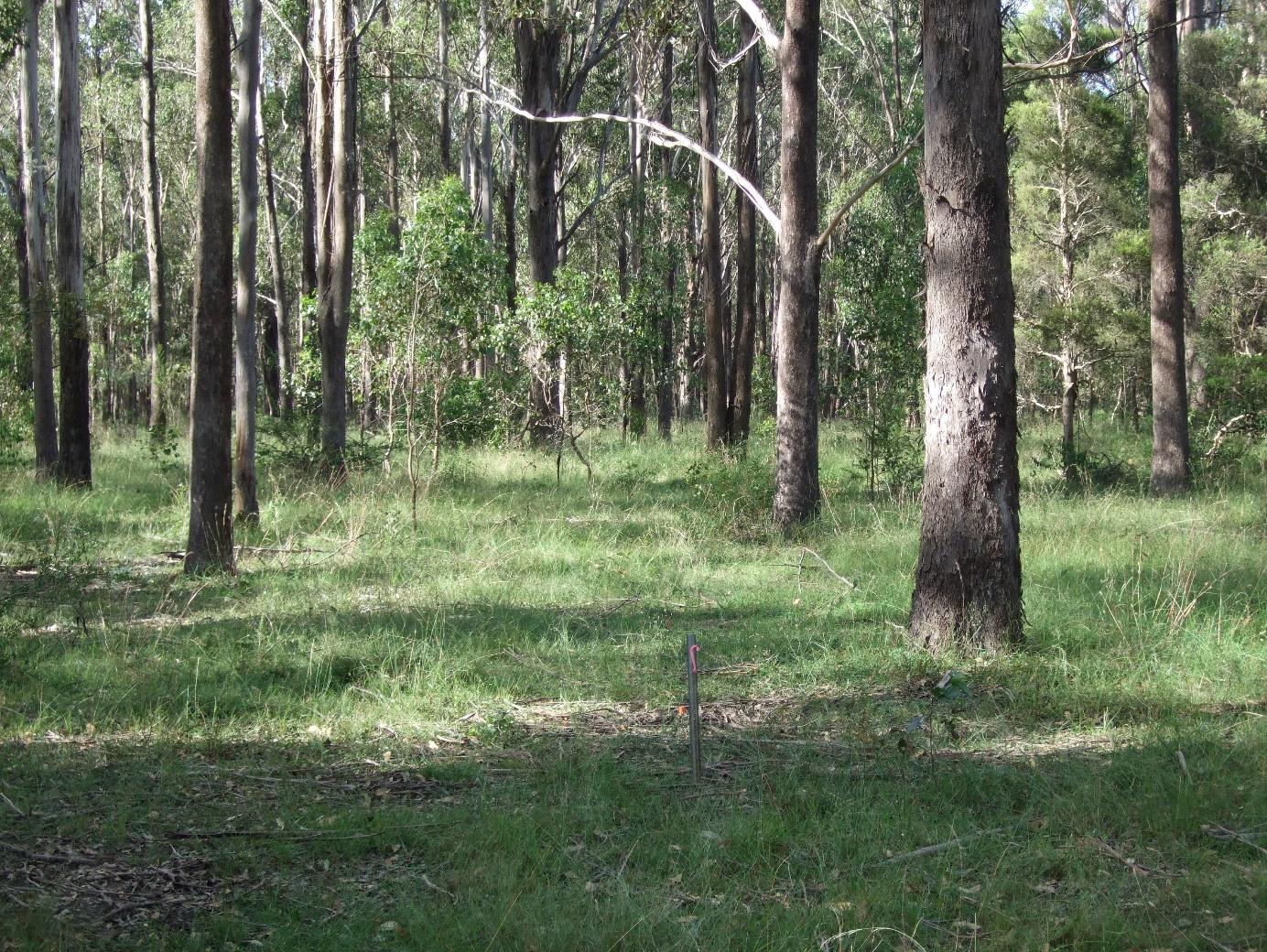


Site Name: AUS - 'Western Sydney


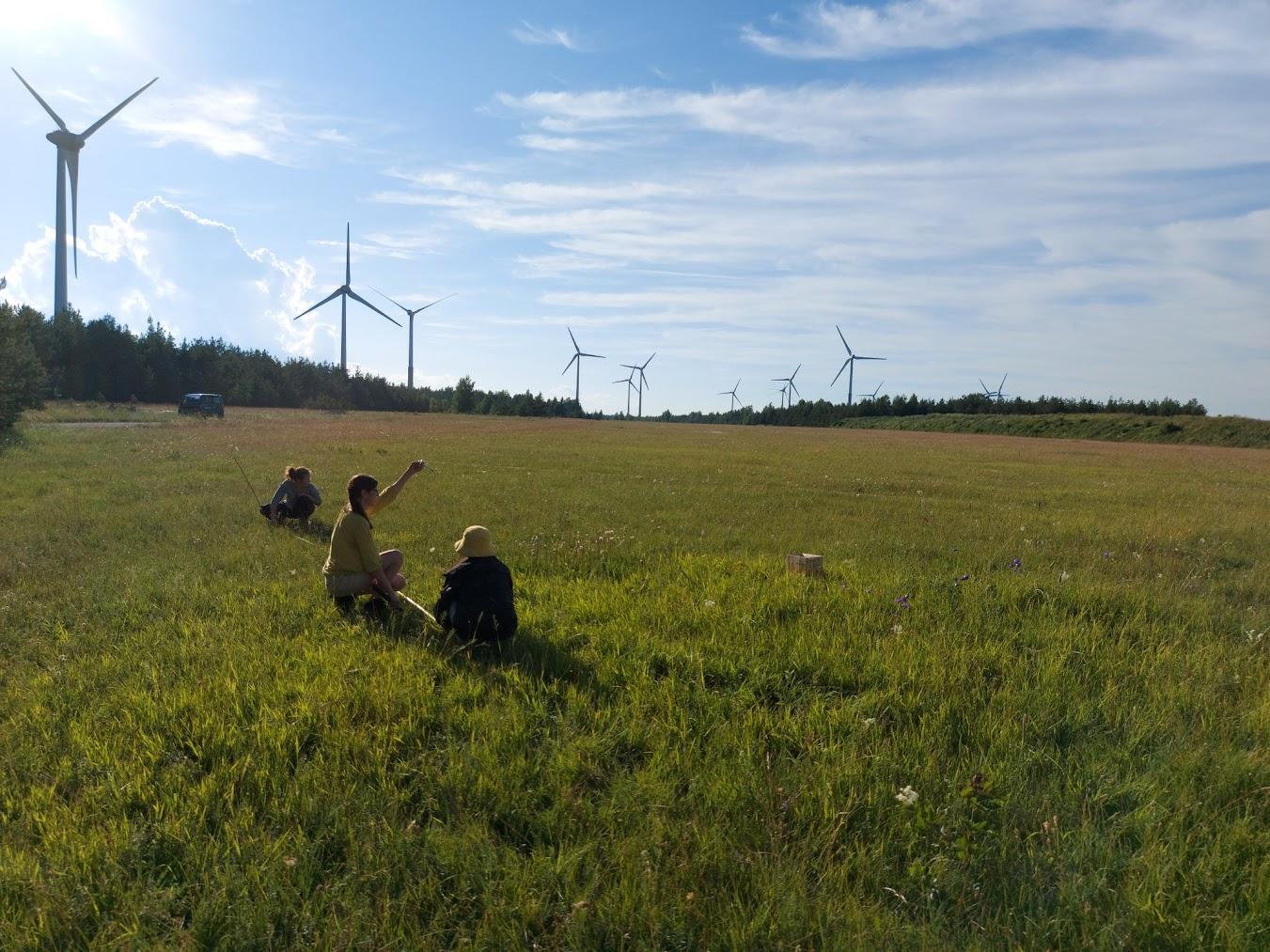


Site Name: EST - Sillukse


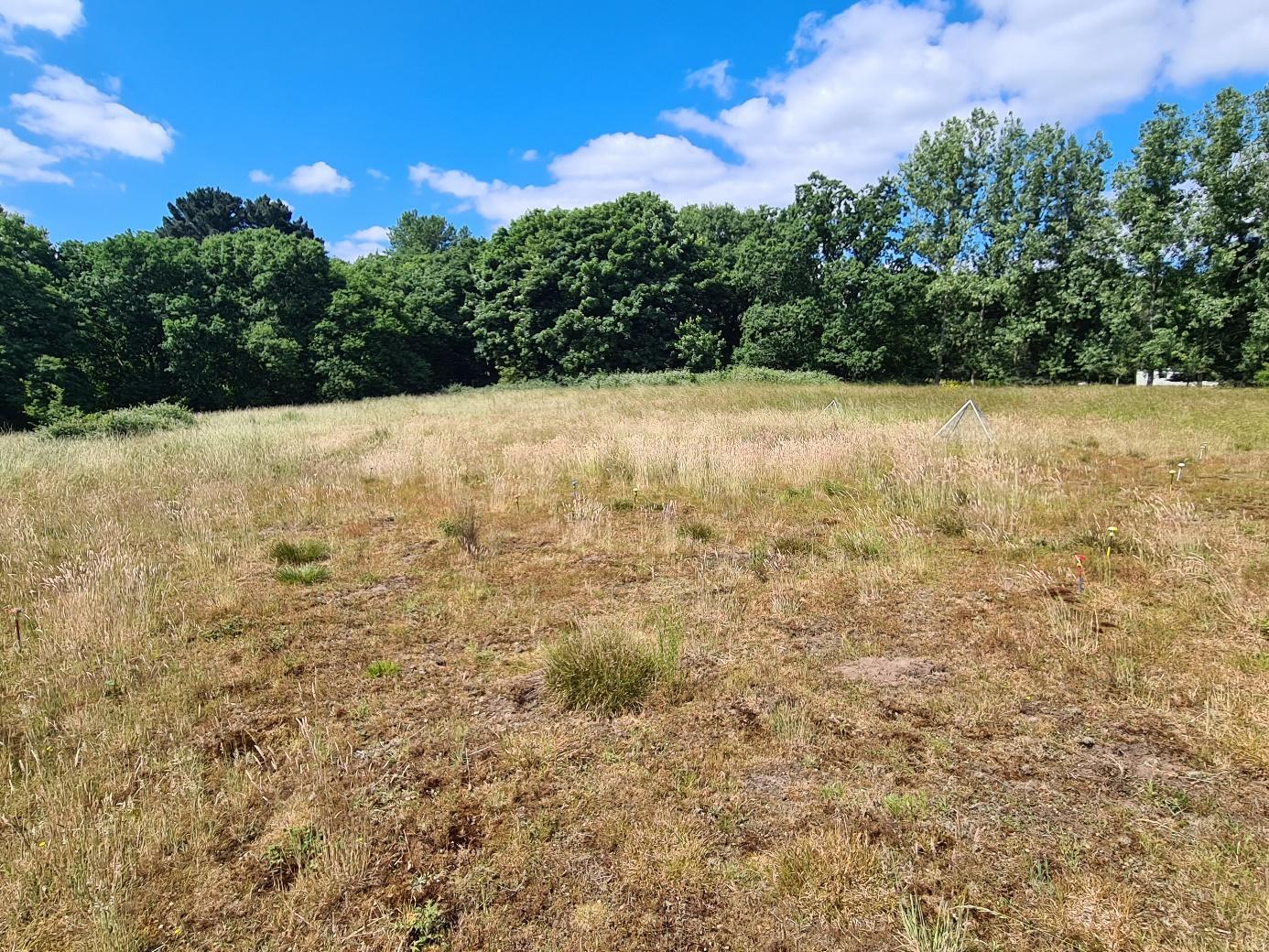


Site Name: UK - Silwood


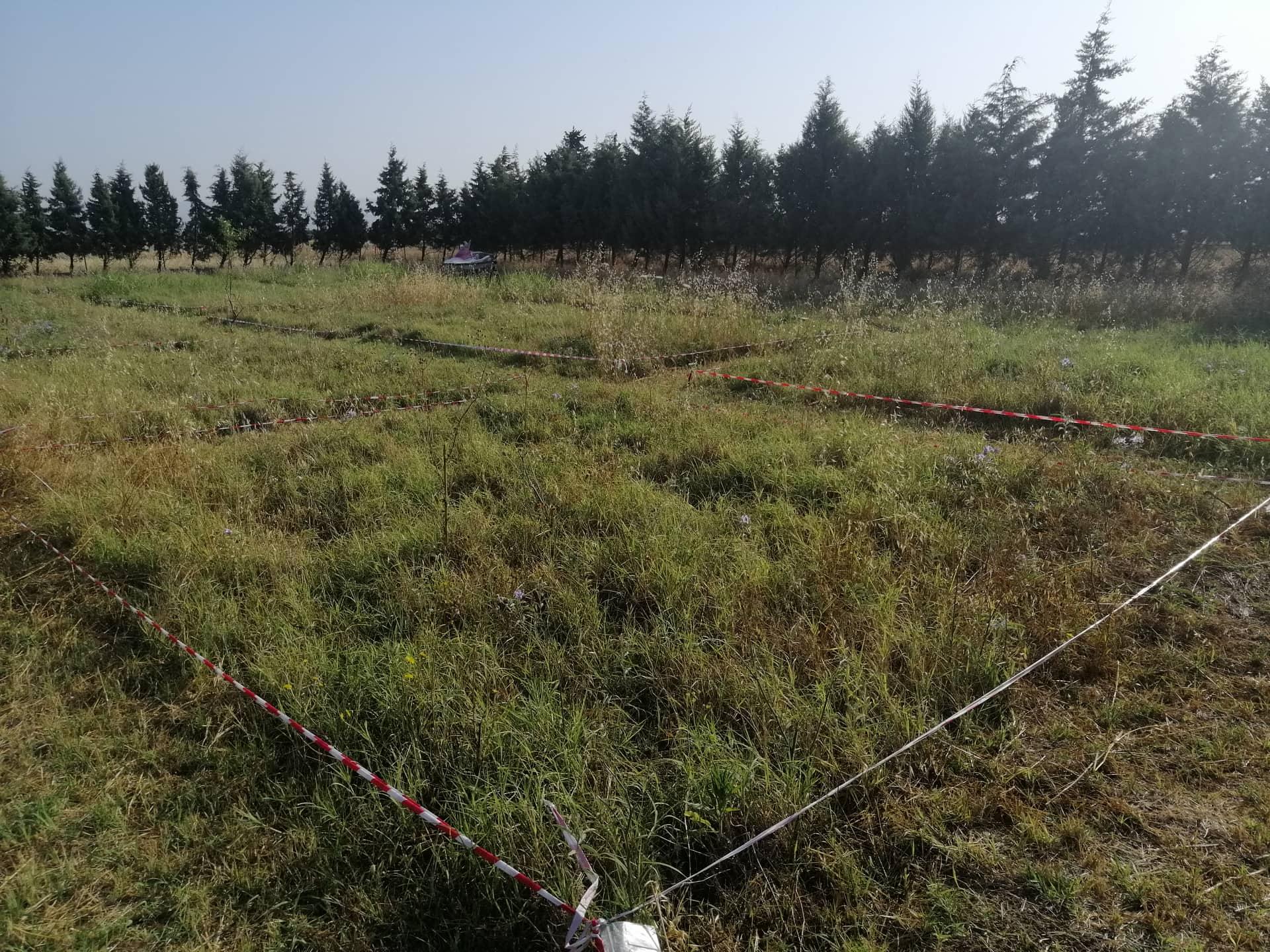


Site Name: GRC - Thessaloniki


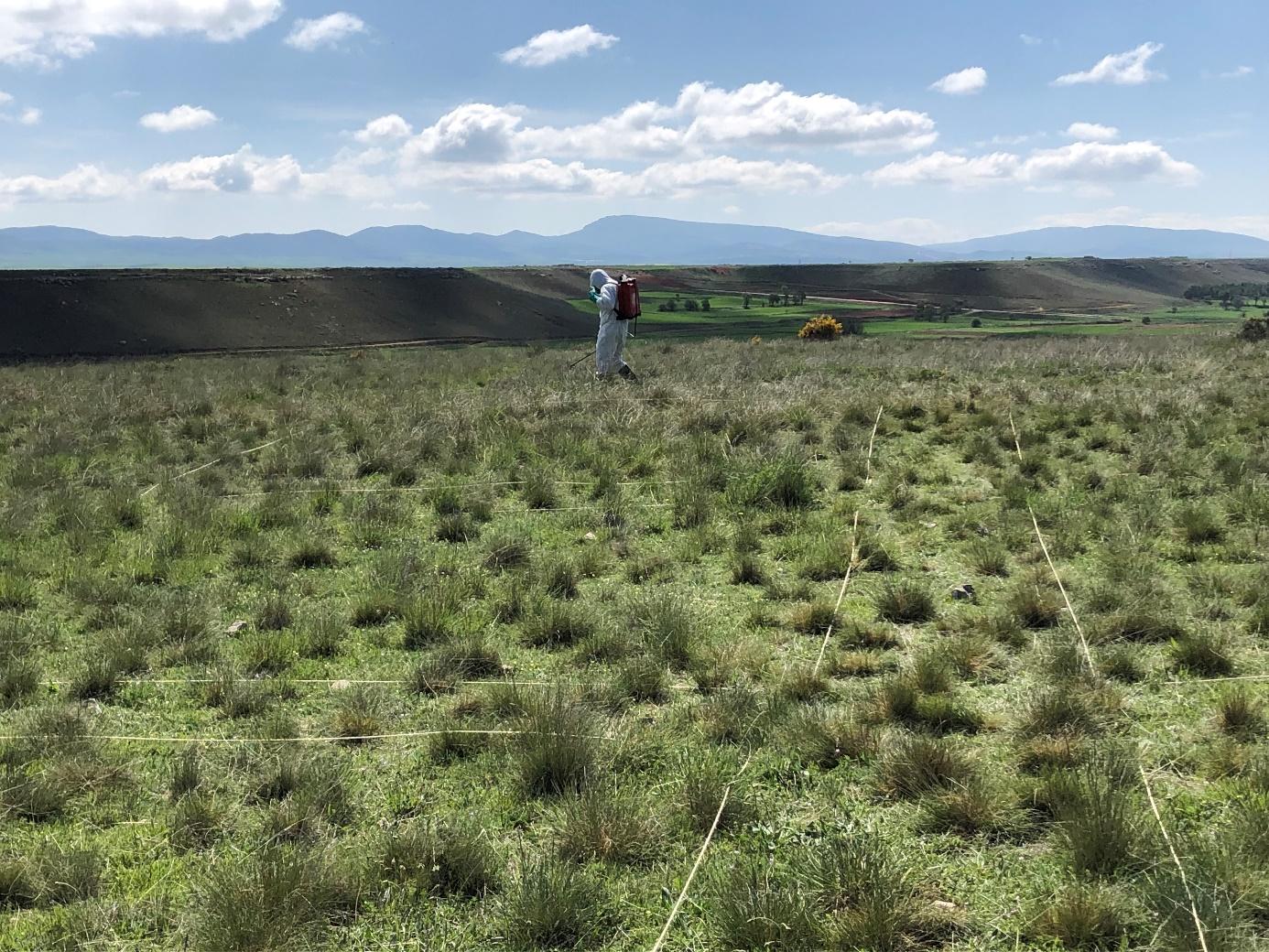


Site Name: ESP - Villaroya


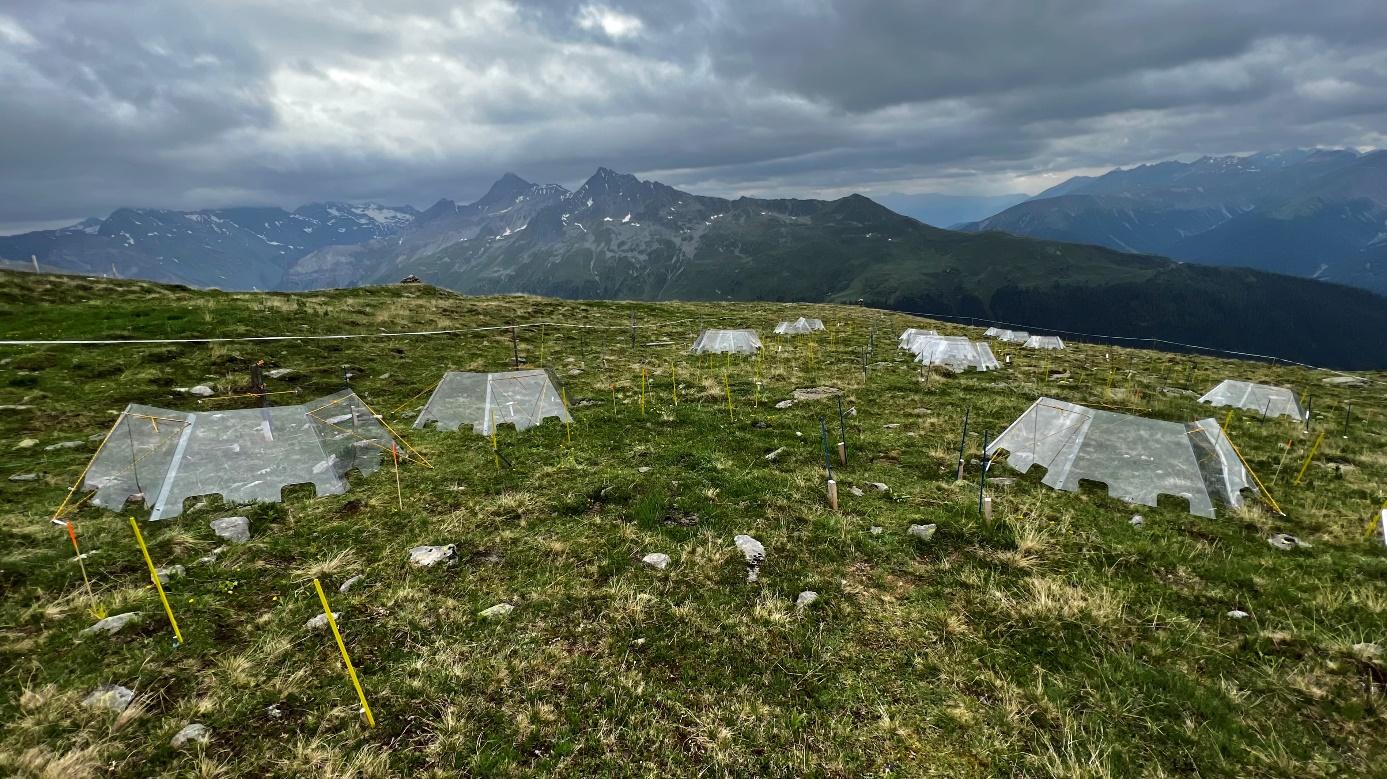


Site Name: CHE – Davos high


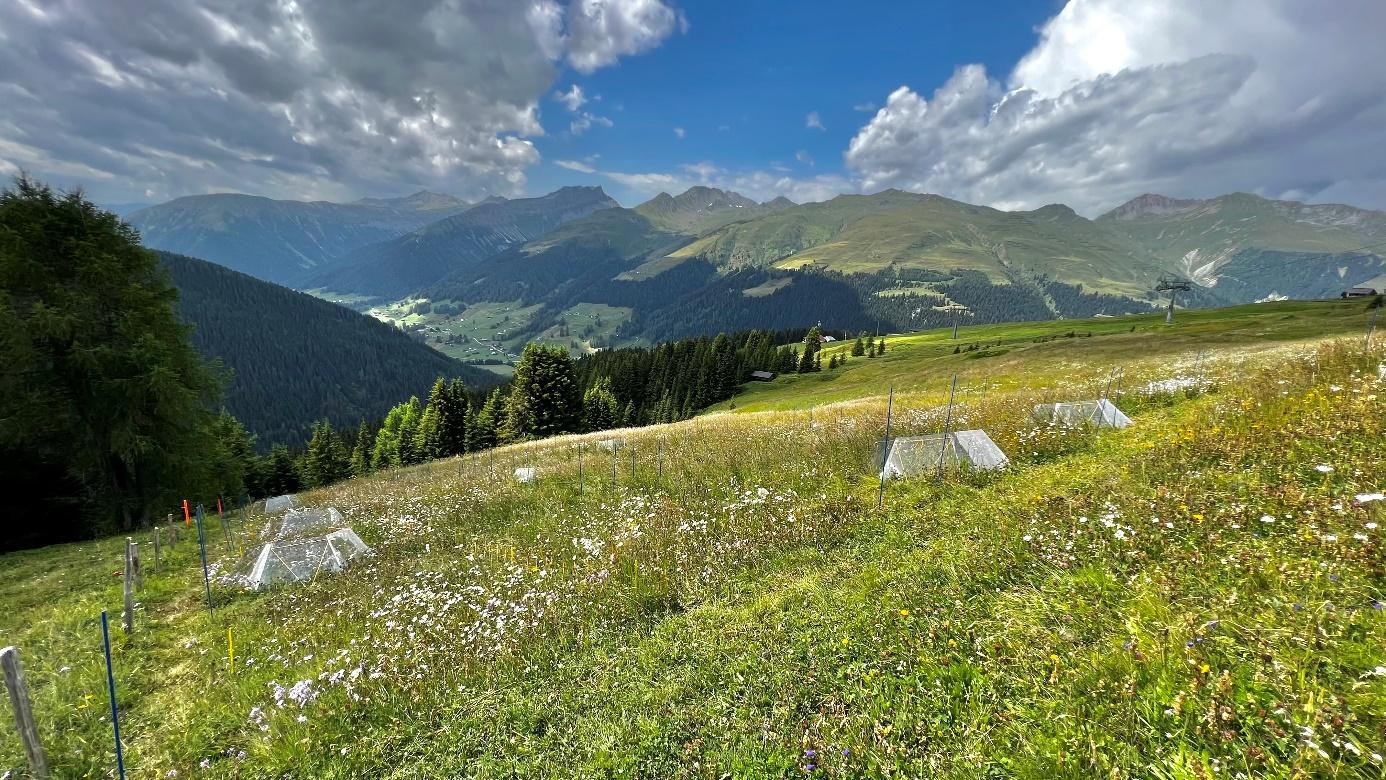


Site Name: CHE – Davos middle


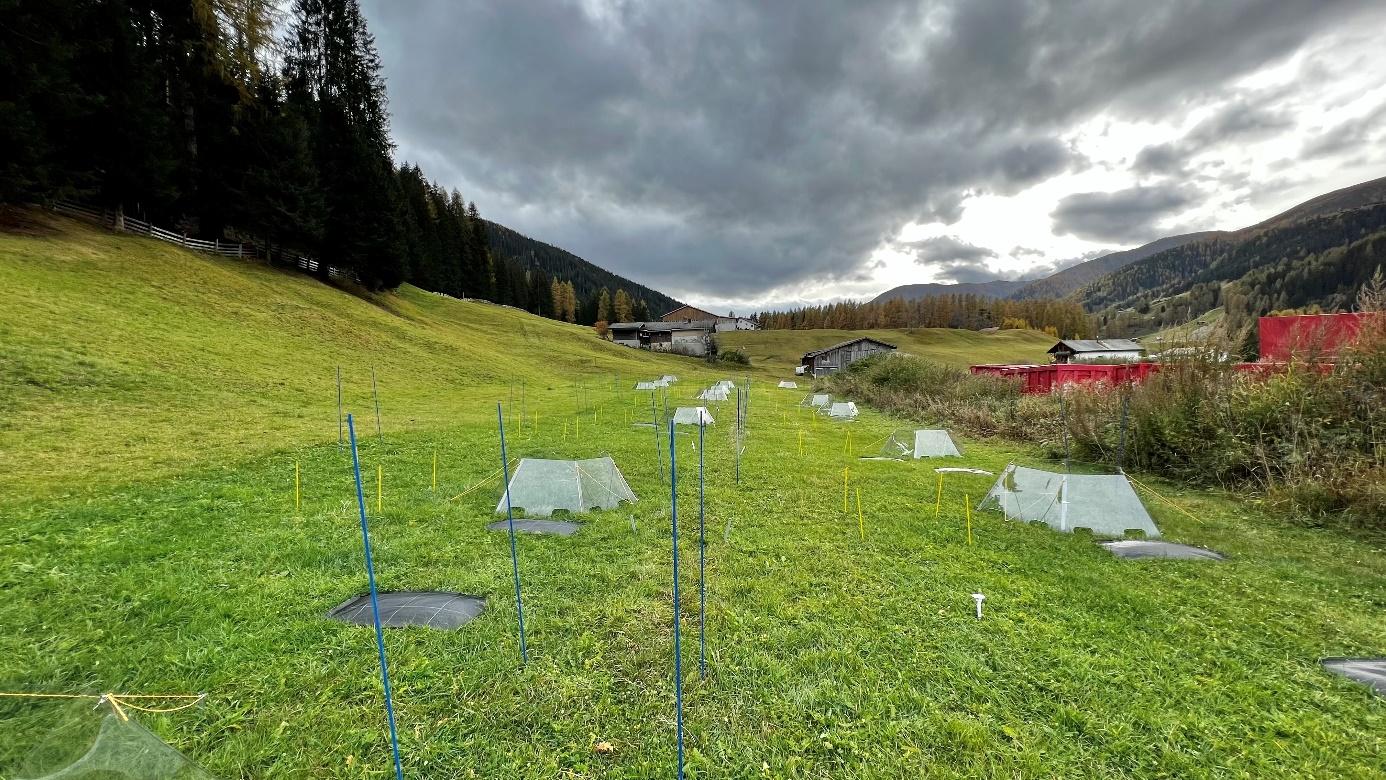


Site Name: CHE – Davos low


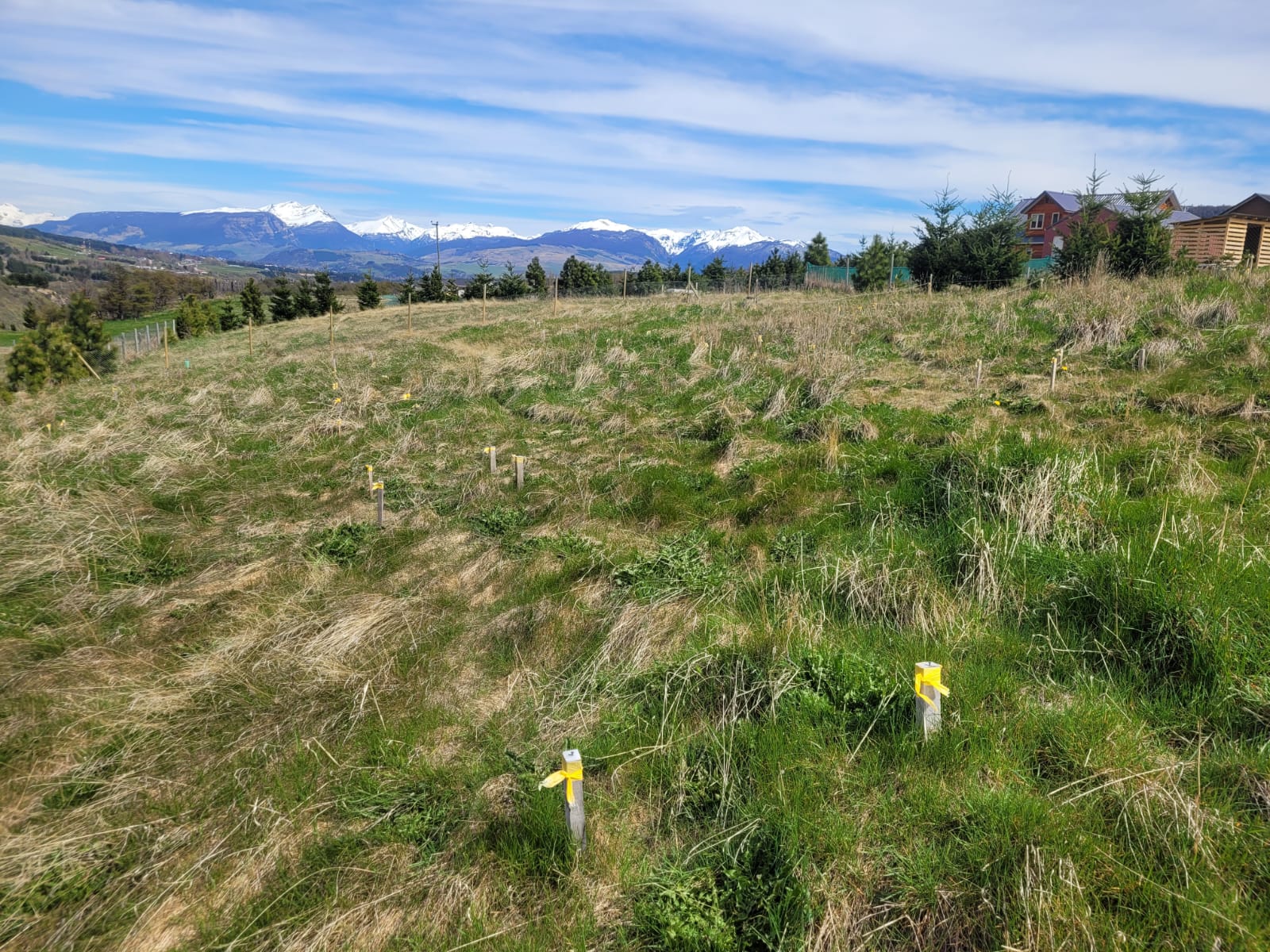


Site Name: CHL – Coyhaique


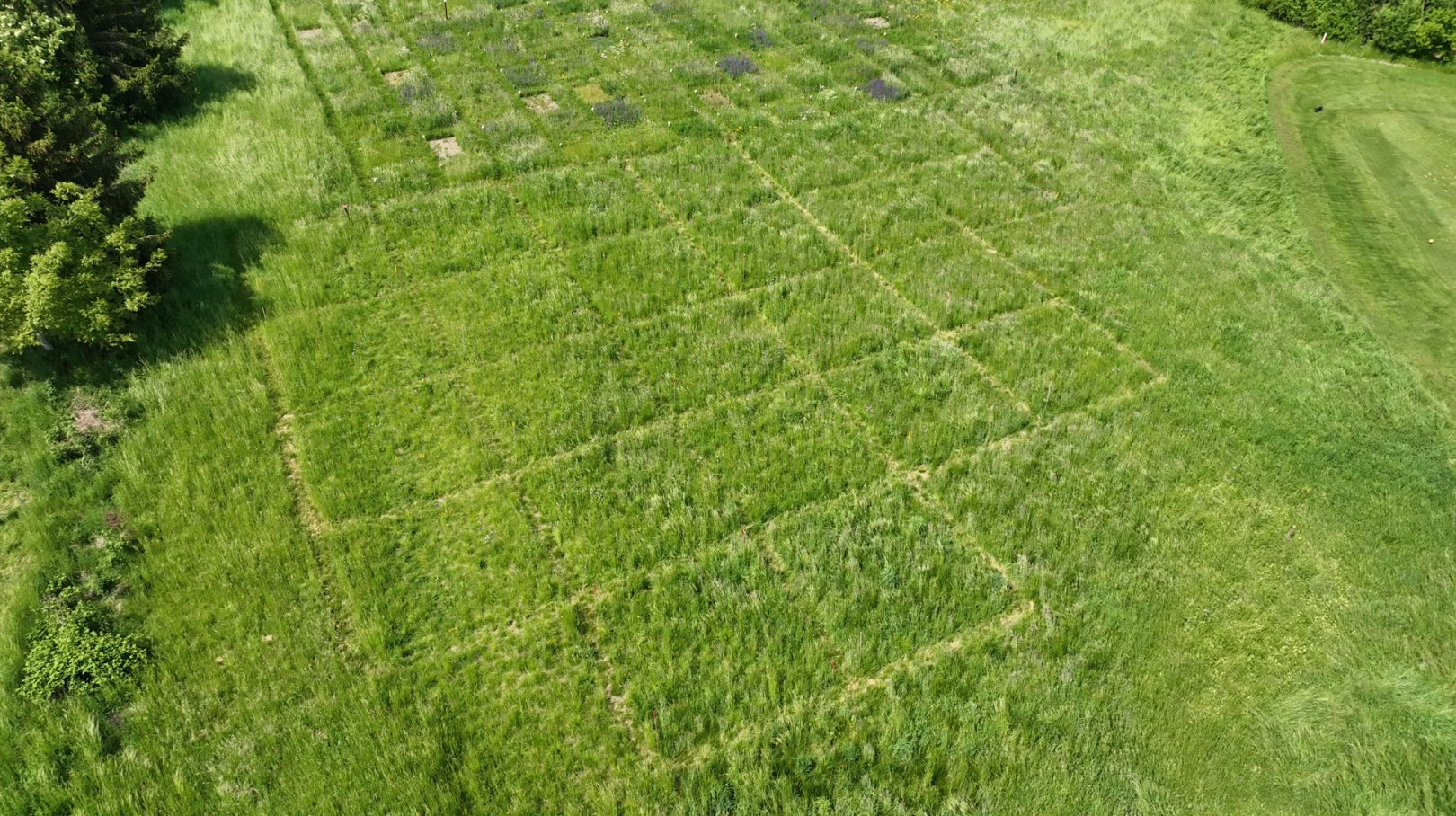


Site Name: CHE – Bern


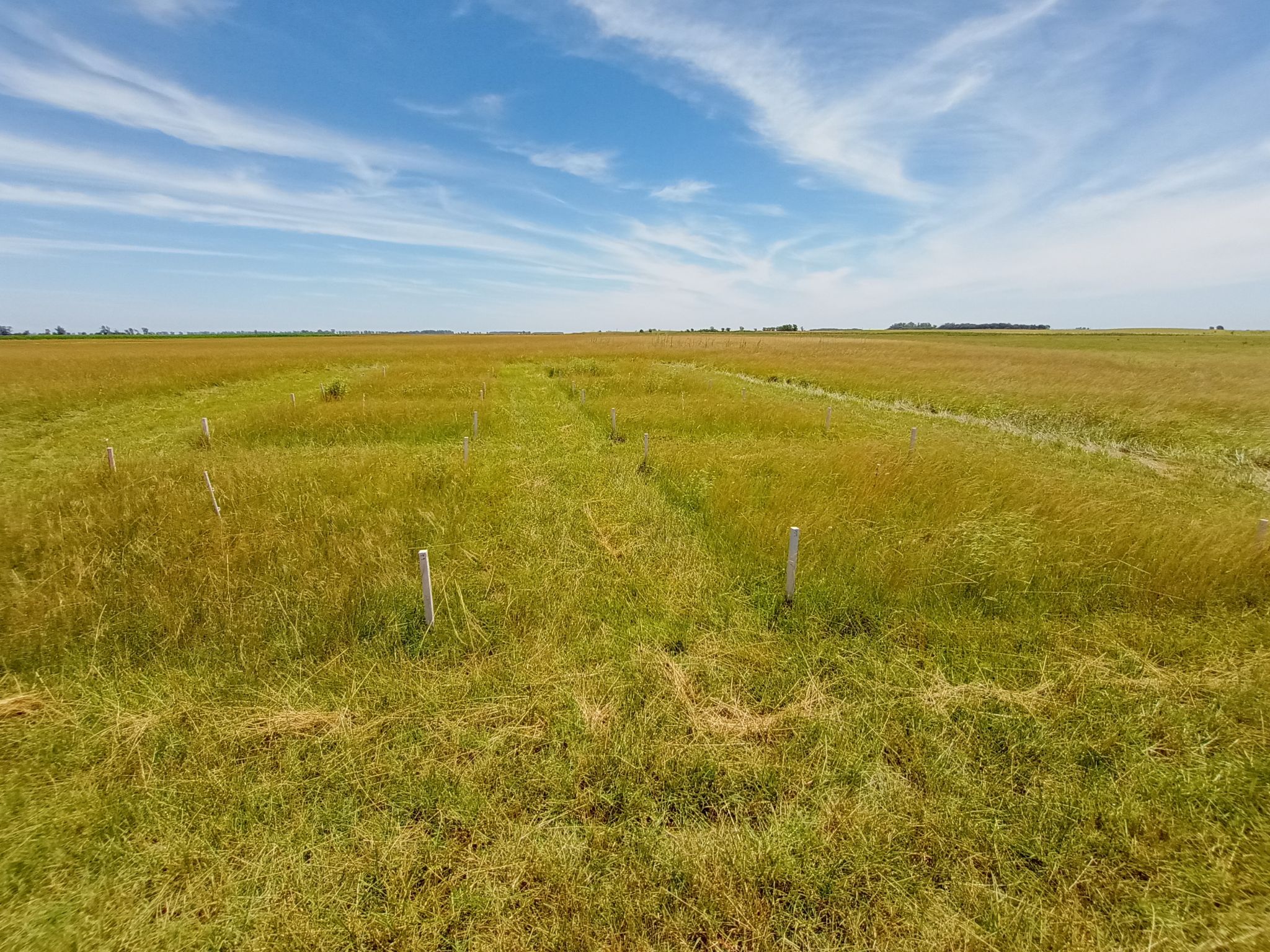


Site Name: ARG - Naredo


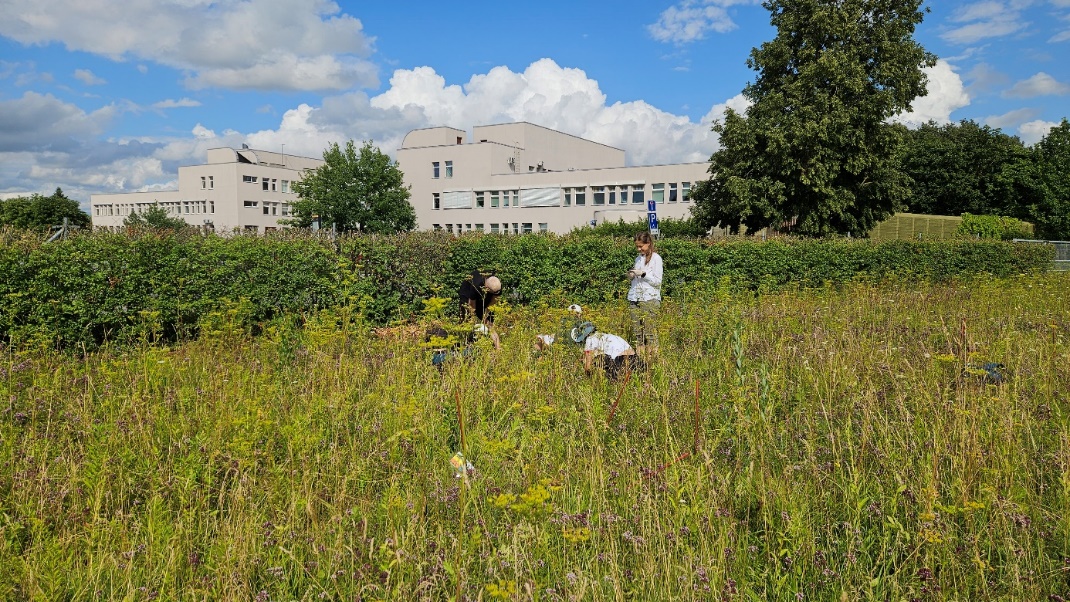


Site Name: EST - Raja


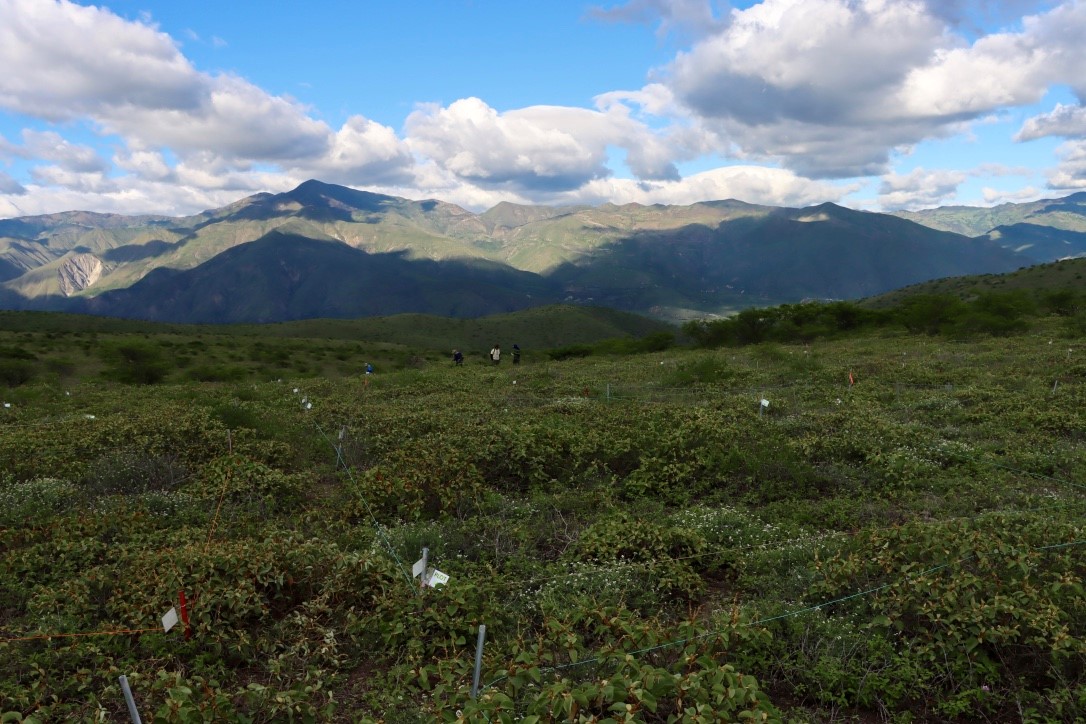


Site Name: ECU - Alama


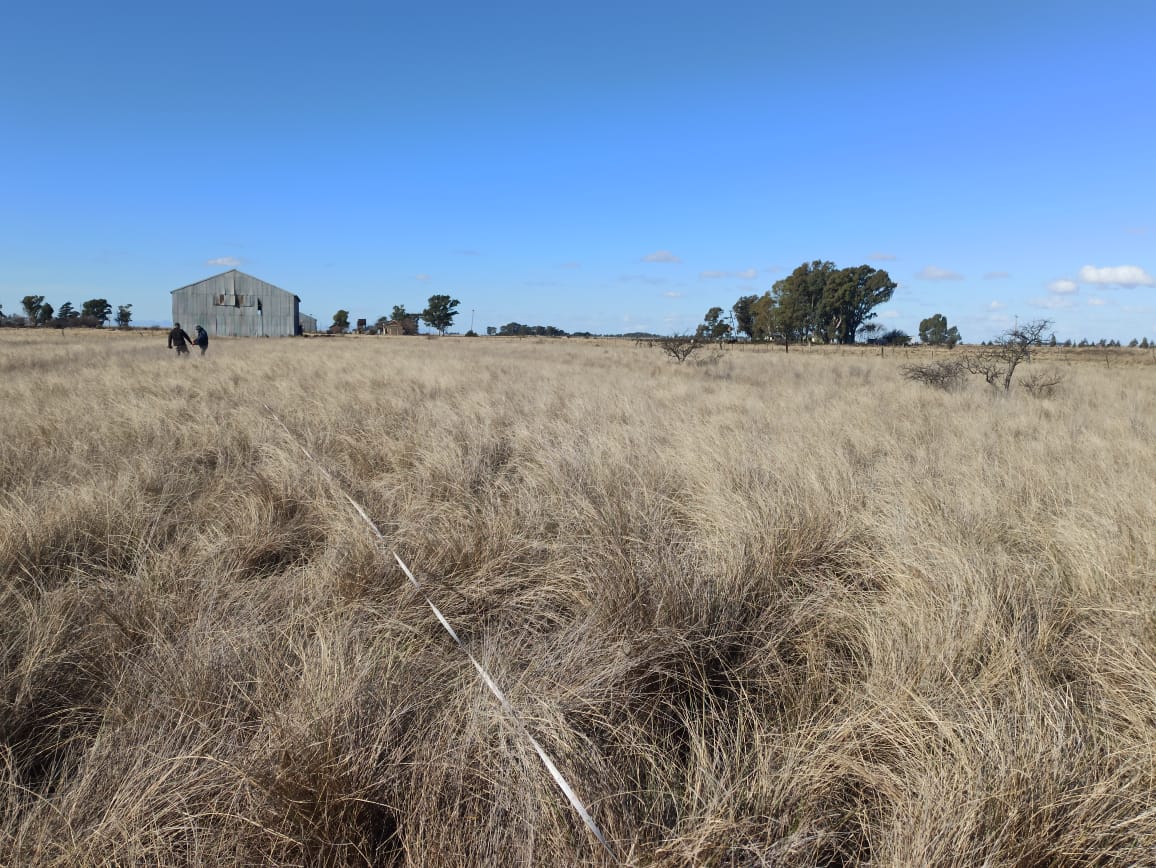


Site Name: ARG - Berraondo


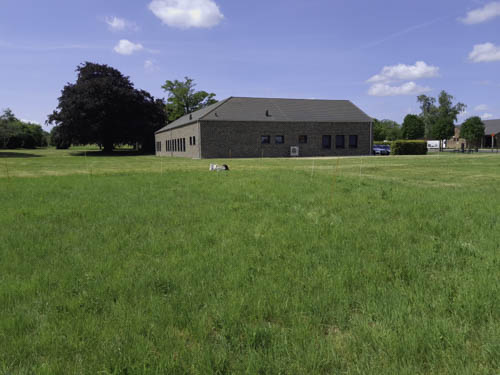


Site Name: GER - Bonn
